# Self‐Amplifying Redox Cycle Triggers Ferroptosis/Cuproptosis Synergy for Enhanced Bacterial Eradication

**DOI:** 10.1002/advs.75101

**Published:** 2026-04-02

**Authors:** Zehui Xiao, Shaolong Qiu, Jiangli Cao, Jifeng Liu, Zhiyong Song, Ting Du, Xinjun Du

**Affiliations:** ^1^ State Key Laboratory of Food Nutrition and Safety College of Food Science and Engineering Tianjin University of Science and Technology Tianjin P. R. China; ^2^ College of Chemistry Huazhong Agricultural University Wuhan P. R. China

**Keywords:** cuproptosis/ferroptosis‐like, Fenton/Fenton‐like reactions, oxidative stress, pneumonia, wound healing

## Abstract

Ferroptosis and cuproptosis, two emerging forms of metal‐dependent cell death, show potential in combating multidrug‐resistant bacterial infections. However, their effectiveness is constrained by insufficient intracellular iron/copper levels, unintended toxicity to healthy cells. Here, we developed a self‐amplifying, targeted nanocomposite (ct@HMCF‐Dex) based on hollow mesoporous copper‐iron sulfide (HMCF), citric acid (ct), and dextran (Dex), which specifically triggers extracellular bacterial cuproptosis/ferroptosis, effectively treating MRSA lung infections and wound infections. Under the bacterial‐infected microenvironment, ct@HMCF‐Dex can release Cu^2+^, Fe^3+^, S^2−^ and citric acid in response to the acidic environment, thereby triggering a series of amplified oxidative stress reactions. During this process, S^2−^ generates H_2_S under acidic conditions, inhibiting the activity of catalase and causing a synergistic effect of local acidification and accumulation of H_2_O_2_. Cu^2+^/Fe^3+^ disrupts redox balance by depleting glutathione while sustaining Cu^+^/Fe^2+^ release via redox cycling. Citric acid chelates Cu^+^/Fe^2+^, prolonging their activity for lipid peroxidation, and activates the tricarboxylic acid (TCA) cycle, synergizing with cysteine depletion to induce cuproptosis/ferroptosis‐like death. This process generates stable hydroxyl radicals (•OH) and effectively clearing free bacteria and disrupting biofilms through a synergistic mechanism. This study presents a precise strategy to combat stubborn infections by triggering targeted bacterial cuproptosis/ferroptosis‐like death.

## Graphical abstract

This study designs a targeted nanocomposite (ct@HMCF‐Dex) that responds to acidic infection microenvironments, releasing components which amplify oxidative stress. It disrupts bacterial redox balance, chelates metals to sustain lipid peroxidation, and synergistically induces cuproptosis/ferroptosis‐like death. This approach effectively eliminates free MRSA and biofilms, offering a precise strategy against resistant infections.

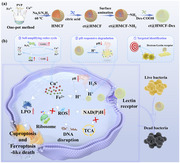

## 1 Introduction

Ferroptosis, an iron‐dependent form of programmed cell death, has emerged as a promising strategy for extracellular and intracellular antimicrobial therapy since iron is a universal and essential component of the life cycle [1, 2]. The prevailing understanding is that ferroptosis occurs when intracellular reactive oxygen species (ROS) drive extensive lipid peroxidation (LPO) of polyunsaturated fatty acids (PUFAs) in the cell membrane through the Fe^2+^ mediated Fenton reaction [3, 4, 5]. While most bacterial membranes primarily consist of saturated or monounsaturated lipids, certain bacteria can either synthesize PUFAs or acquire them from external sources and incorporate them into their membranes [6]. These PUFAs are highly susceptible to oxidation, rendering them potential substrates for ferroptosis signaling. Given that iron metabolism is also inherent to bacteria [7], a promising antibacterial approach involves enhancing the uptake of labile iron ions while promoting LPO through the coordinated delivery of Fe^2+^ and ROS to induce ferroptosis. However, the antimicrobial consequences of disrupting iron homeostasis are not restricted to this pathway. Several studies have reported potent antibacterial effects mediated by iron deprivation or interference that do not primarily depend on LPO or a global ROS burst, such as through the chelation of essential iron or the disruption of iron‐dependent metabolic pathways [8, 9, 10]. These alternative mechanisms may operate independently or in synergy with ferroptosis, together representing an effective means of eradicating bacteria.

The distinct form of bacterial cell death triggered by excessive Cu^2+^ overload has been termed cuproptosis‐like death [11]. In this process, copper‐based nanomaterials provide sustained release of Cu^2+^ and Cu^+^, facilitating bacterial uptake. In the cytoplasm, Cu(II) is reduced to Cu(I). Upon internalization, Cu^+^ binds to lipoylated proteins in the glyoxylate and tricarboxylic acid (TCA) cycles, inducing their abnormal oligomerization and subsequent disruption of essential metabolic pathways [12, 13, 14]. Concurrently, Cu^2+^ can directly impair the oxidative respiratory chain and promotes lipid peroxides accumulation, resulting in fatal oxidative damage to bacterial cells [15]. Li et al. confirmed that sonodynamic‐enhanced Cu(I) overload and hypoxia‐activated therapy inhibited the TCA cycle, leading to bacterial cuproptosis‐like death [13]. Huang et al. pointed out that intracellular copper overload limited the TCA cycle and promoted bacterial cuproptosis‐like death [16]. This unique bactericidal mechanism, which mimics the cuproptosis death observed in eukaryotic cells, is particularly effective against drug‐resistant strains.

Iron‐based nanomaterials have long been integral to biomedical applications due to their exceptional biocompatibility [17]. While iron ions possess inherent antimicrobial properties, their efficacy under physiological conditions remains limited and varies across iron species [18]. Beyond the intrinsic constraints of iron itself, current ferroptosis‐mimicking strategies face further external challenges, including insufficient hydrogen peroxide (H_2_O_2_) supply, suboptimal catalytic pH conditions, and inefficient delivery of Fenton‐active agents to bacterial cells [19, 20, 21]. In parallel, bacteria deploy intrinsic defense systems that counteract ferroptosis‐like oxidative damage. Key among these are the Per (peroxidase/peroxiredoxin) system, which efficiently detoxifies H_2_O_2_ and organic peroxides [22], and the Fur‐regulated iron homeostasis network, which restricts the availability of labile iron and suppresses Fenton‐driven LPO [23]. Together, these endogenous mechanisms significantly limit ROS accumulation and membrane damage, presenting an additional internal barrier to ferroptosis‐based antimicrobial approaches. Furthermore, cuproptosis‐inspired antimicrobial approaches are constrained by high glutathione (GSH) levels, low bioavailability of free Cu^2+^, and poor bacterial targeting specificity [24]. To overcome these limitations, we rationally designed a multifunctional nanoplatform capable of controlled and spatially precise co‐delivery of Fe^2+^ and Cu^2+^ could synergistically activate cuproptosis/ferroptosis‐like pathways, thereby improving bacterial clearance efficiency.

**SCHEME 1:**
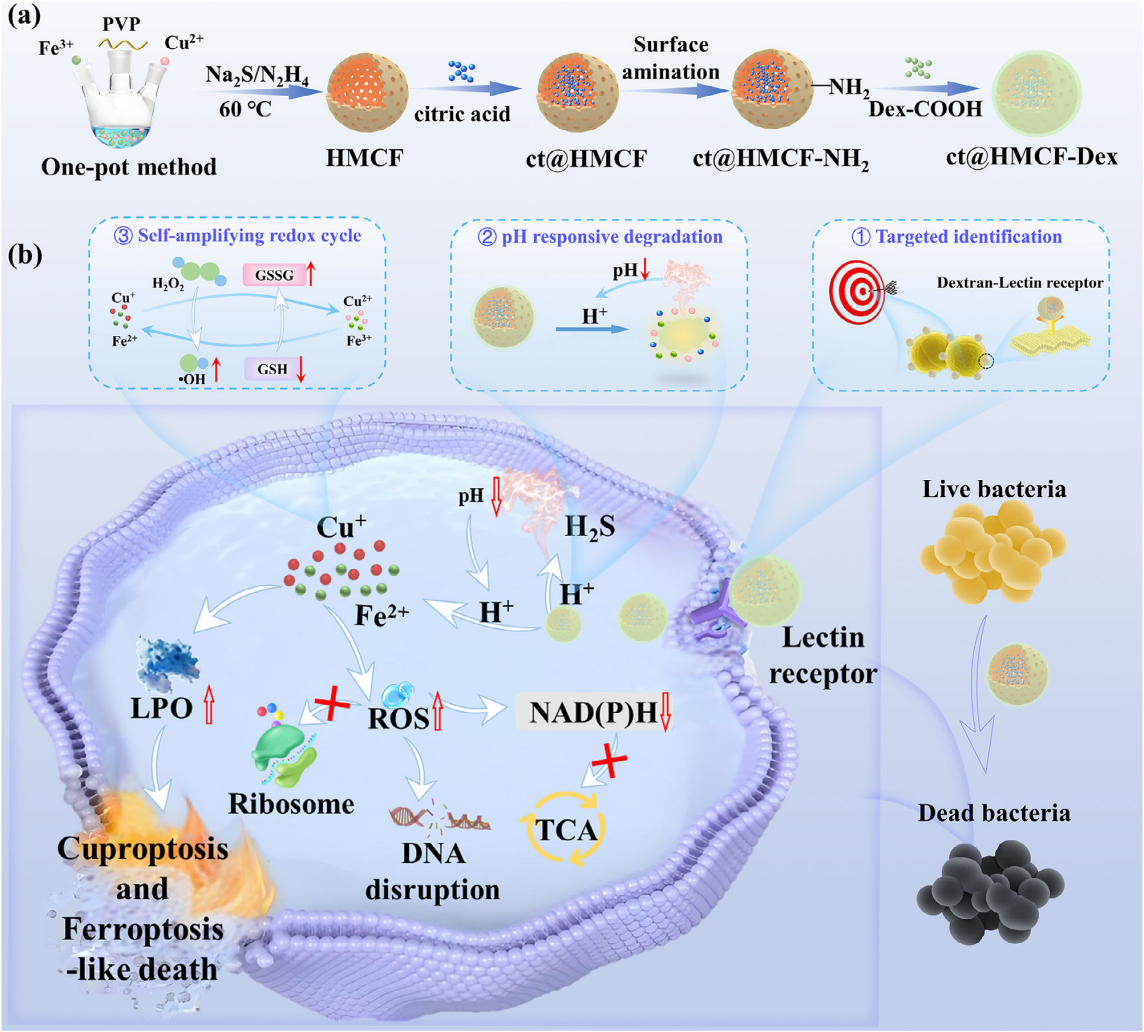
(a) Schematic illustration for the construction of ct@HMCF‐Dex nanoplatforms, (b) the strategy for potent antibacterial therapy through a self‐amplifying redox cycle of ferroptosis/cuproptosis.

Herein, this study presents a novel “pouring fuel on the fire” strategy that establishes an auto‐amplifying symbiotic cycle between iron and copper for highly effective antimicrobial therapy. First, hollow mesoporous copper‐iron sulfide (HMCF) nanoparticles were synthesized via a one‐pot approach based on the Kirkendall diffusion effect. Citric acid (ct) was then incorporated as a synergistic agent into the HMCF core through chelation with copper and iron ions, forming ct@HMCF. To improve stability and enable bacterial targeting, the ct@HMCF nanoparticles were further aminated and surface‑functionalized with carboxylated dextran (Dex‑COOH), yielding the final construct ct@HMCF‑Dex (Scheme 1a). As depicted in Scheme 1b, the ct@HMCF‐Dex nanoplatform can precisely target bacterial cells. In the bacteria‐infection slightly acidic stage, ct@HMCF‐Dex acts as an activated “smart bomb,” releasing citric acid, S^2−^, Cu^2+^, and Fe^2+^. Under acidic conditions, S^2−^ generates H_2_S, which stimulates lactate production and proton accumulation, further acidifying the local bacterial environment and elevating H_2_O_2_ levels [25, 26]. This progressive acidification accelerates the destabilization of ct@HMCF‐Dex, promoting sustained ion release. The released Fe^2+^ and Cu^+^ subsequently engage in Fenton and Fenton‑like reactions with H_2_O_2_, generating highly toxic •OH while being oxidized to Fe^3+^ and Cu^2+^. Furthermore, Fe^3+^ interacts with GSH, converting it to GSSG and regenerating Fe^2+^, while electron exchange between Cu^2+^ and Fe^3+^ produces Cu^+^ and Fe^2+^. Together, these steps establish a self‑sustaining catalytic cycle. In this cycle, the released citrate acts as “fuel,” and its strong chelation with Fe(III/II) and Cu(II/I) further liberates the metal ions, thereby intensifying bacterial oxidative stress and “adding fuel to the fire.” The acidification induced by H_2_S additionally accelerates these Fenton‑like reactions, producing more •OH inside bacterial cells. Concurrently, elevated levels of Fe^3+^ and Cu^2+^ deplete GSH and disrupt bacterial antioxidant defenses, synergistically amplifying ferroptosis‑ and cuproptosis‑like cell death. Through these coupled processes, the nanoplatform is designed to construct a locally confined, self‑amplifying oxidative‑stress microenvironment within bacteria, characterized by sustained acidification, continuous redox cycling, and persistent ROS generation. This integrated design is expected to synergistically disrupt bacterial redox homeostasis, lipid metabolism, and antioxidant defense systems, ultimately leading to potent antibacterial effects via ferroptosis‑ and cuproptosis‑like pathways.

## 2 Results and Discussion

### 2.1 Synthesis and Characterization of ct@HMCF‐Dex Nanoplatforms

**FIGURE 1:**
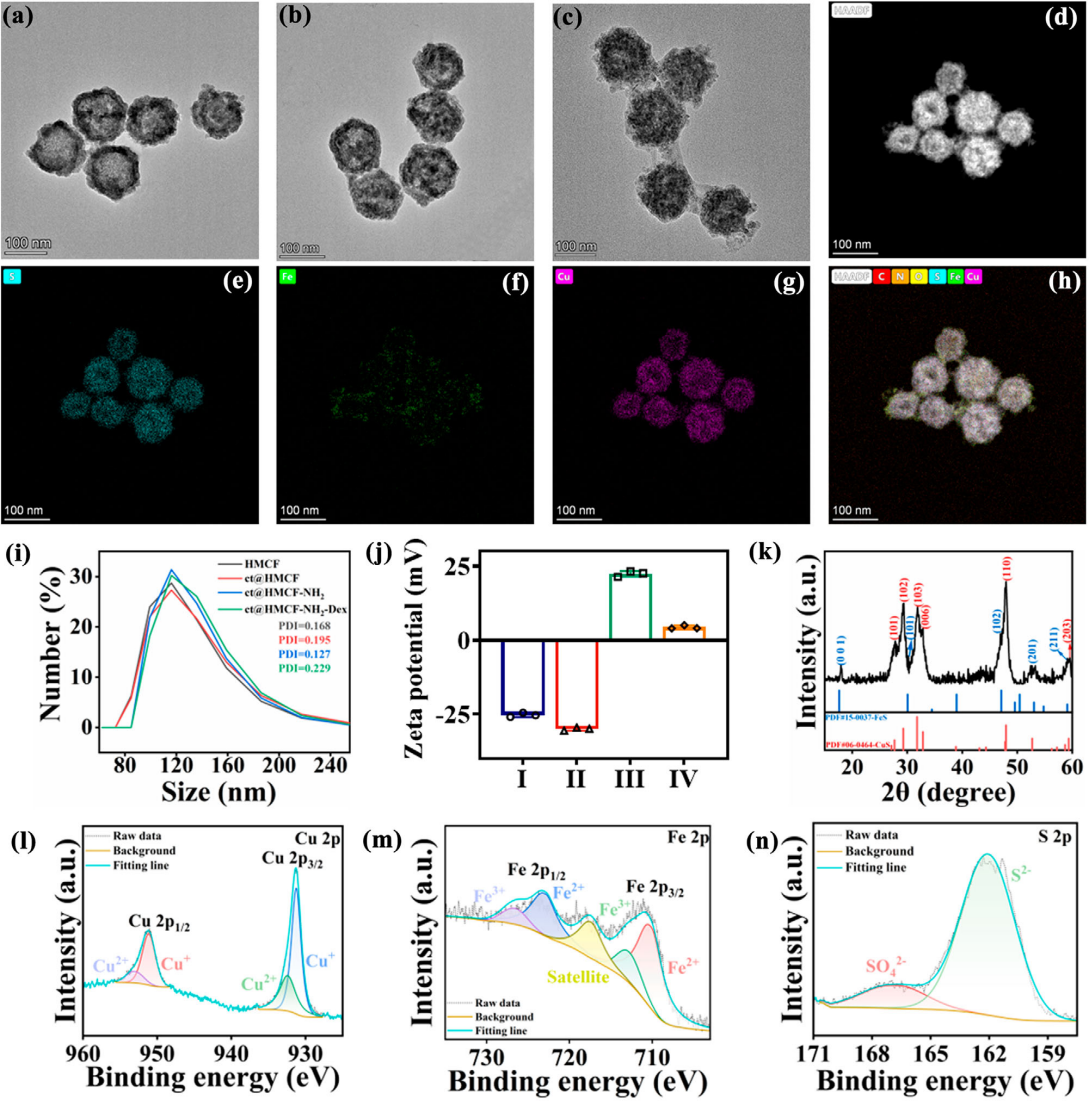
Characterization of ct@HMCF‐Dex. TEM image of HMCF (a), ct@HMCF (b), and ct@HMCF‐Dex (c). (d–h) Element mapping images of ct@HMCF‐Dex. (i) Hydrodynamic size distributions of HMCF, ct@HMCF, and ct@HMCF‐Dex. (j) Zeta potential of HMCF (I), ct@HMCF (II), ct@HMCF‐NH_2_ (III), and ct@HMCF‐Dex (IV) (*n* = 3). Error bars represent means ± SD. (k) XRD pattern of the ct@HMCF‐Dex. Cu 2p (l), Fe 2p (m), and S 2p (n) XPS spectrum of ct@HMCF‐Dex.

Initially, the HMCF nanoparticles were synthesized via a one‐pot method utilizing the Kirkendall diffusion effect [27, 28]. As shown in Figure 1a, the prepared HMCF are uniformly spherical (∼120 nm in diameter) with a hollow structure evident from their translucent cores. Figure S1a further shows that HMCF exhibits a distinct lattice structure with a spacing of 267 pm. Next, citric acid (ct) was encapsulated to form ct@HMCF (Figure 1b). The successful loading of citric acid was confirmed by two independent quantitative methods. Using a commercial assay kit, the citric acid loading capacity in ct@HMCF was determined to be 31.06% ± 2.73% (Figure S2a). This result was corroborated by thermogravimetric analysis (TGA, Figure S2b), which showed progressively increased weight loss from HMCF to ct@HMCF and finally to ct@HMCF‐Dex. The mass losses attributable to citric acid and dextran in ct@HMCF‐Dex were calculated as 31.44% and 16.96%, respectively. Finally, the ct@HMCF nanoparticles underwent surface amination followed by Dex‐COOH conjugation, yielding ct@HMCF‐Dex. As shown in Figure 1b,c, ct@HMCF and ct@HMCF‐Dex maintained their spherical morphology while exhibiting darker contrast due to partial occlusion of their hollow interiors. Importantly, the citric acid content remained largely unchanged after surface modification (30.88% ± 2.57% for ct@HMCF‐Dex), indicating no significant leakage occurred during the dextran functionalization process (Figure S2a). Elemental mapping via high‐angle annular dark‐field scanning transmission electron microscopy (HAADF‐STEM) clearly demonstrated the presence and homogeneous distribution of C (Figure S1b), N (Figure S1c), O (Figure S1d), S (Figure 1e), Fe (Figure 1f), and Cu (Figure 1g) in ct@HMCF‐Dex. These findings were further corroborated by energy‐dispersive X‐ray spectroscopy (EDX) analysis (Figure S1e), thereby confirming the successful fabrication of the ct@HMCF‐Dex nanoparticles.

The size distribution of HMCF, ct@HMCF, and ct@HMCF‐Dex nanoparticles was evaluated using dynamic light scattering (DLS). In Figure 1i, the hydrodynamic diameters of HMCF, ct@HMCF, ct@HMCF‐NH_2_, and ct@HMCF‐Dex were 125.96 nm, 129.54, 130.13, and 133.18 nm, respectively. All samples exhibited excellent monodispersity with polydispersity indices (PDI) below 0.3, indicating highly uniform particle size distributions [29, 30]. Zeta potential measurements (Figure 1j) show the values of HMCF, ct@HMCF, ct@HMCF‐NH_2_, and ct@HMCF‐Dex are approximately −25.25, −30.01, 22.43, and 4.40 mV, respectively. Ultraviolet–visible (UV–vis) spectroscopy analysis (Figure S1f) indicates that these nanoparticles have a wide absorption range within the 600–900 nm range. This prominent absorption within the biological tissue transparency window provides a foundation for their potential application in photothermal therapy (PTT). Furthermore, Fourier‐transform infrared (FT‐IR) spectroscopy was employed to characterize the synthesis of ct@HMCF‐Dex (Figure S1g). The spectrum of ct@HMCF exhibited characteristic carboxylate (‐COO^−^) stretching vibrations at 1590 and 1390 cm^−1^. Successful amination to form ct@HMCF‐NH_2_ was confirmed by the appearance of an N–H bending vibration (δ_N–H) at 1654 cm^−1^. Finally, the ct@HMCF‐Dex spectrum displayed key vibrational bands indicative of dextran conjugation, notably an amide C═O stretch at 1657 cm^−1^ and a C─O stretch at 1019 cm^−1^ [31].

The XRD pattern of ct@HMCF‐Dex nanocomposites exhibits 11 distinct diffraction peaks corresponding to both CuS (indexed to (101), (102), (103), (006), (110), and (203) lattice planes) and FeS ((001), (101), (102), (201), and (211) lattice planes) crystalline phases (Figure 1k). The X‐ray photoelectron spectroscopy (XPS) analysis revealed characteristic peaks corresponding to S 2p, C 1s, N 1s, O 1s, Fe 2p, and Cu 2p in ct@HMCF‐Dex (Figure S3a). These findings are consistent with the aforementioned EDX results. The high‐resolution XPS spectrum of Cu 2p (Figure 1l) displays four prominent peaks located at 931.25 eV (Cu 2p_3/2_) and 951.13 eV (Cu 2p_1/2_), which correspond to Cu^+^, or at 932.38 eV (Cu 2p_3/2_) and 953.02 eV (Cu 2p_1/2_), which are attributed to Cu^2+^ [16]. The Fe 2p peaks of Fe^2+^ were located at 710.11 eV (Fe 2p_3/2_) and 722.91 eV (Fe 2p_1/2_), respectively, while the peaks of Fe^3+^ were located at 712.8 eV (Fe 2p_3/2_) and 726.6 eV (Fe 2p_1/2_), respectively, with their satellite peaks at 718.8 eV (Figure 1m) [32]. These results confirm that both Cu^2+^/Cu^+^ and Fe^3+^/Fe^2+^ coexist in the ct@HMCF‐Dex system. Quantitative analysis revealed that Cu^+^ constitutes approximately 81.52% of total copper, and Fe^2+^ represents about 64.69% of total iron, highlighting the potential of ct@HMCF‐Dex in inducing bacterial cuproptosis/ferroptosis‐like effects. Moreover, the high‐resolution S spectrum (Figure 1n) exhibits binding energies at 166.98 and 162.08 eV, which are attributed to sulfate (SO_4_^2−^) and sulfide (S^2−^), respectively [33]. Furthermore, the high‐resolution spectra of C (Figure S3b), N (Figure S3c), and O (Figure S3d) exhibit binding energies at 284.68, 399.08, and 530.98 eV, indicating the presence of C─C bonds, pyrrole nitrogen, and oxygen‐containing carbon functional groups in ct@HMCF‐Dex [34]. Collectively, these findings confirm the successful synthesis of the ct@HMCF‐Dex nanocomposites. Furthermore, we monitored the changes in hydrodynamic diameter and surface charge of ct@HMCF‐Dex in different environments (containing water, PBS (pH 7.4), DMEM, and RPMI 1640) over a 96 h period using DLS analysis. As shown in Figure S4, the hydrated particle size of ct@HMCF‐Dex remained around 133 nm across various environments and time points, while the surface charge remained consistent.

### 2.2 In Vitro Validation of pH‐Dependent Degradability and ROS Production

**FIGURE 2:**
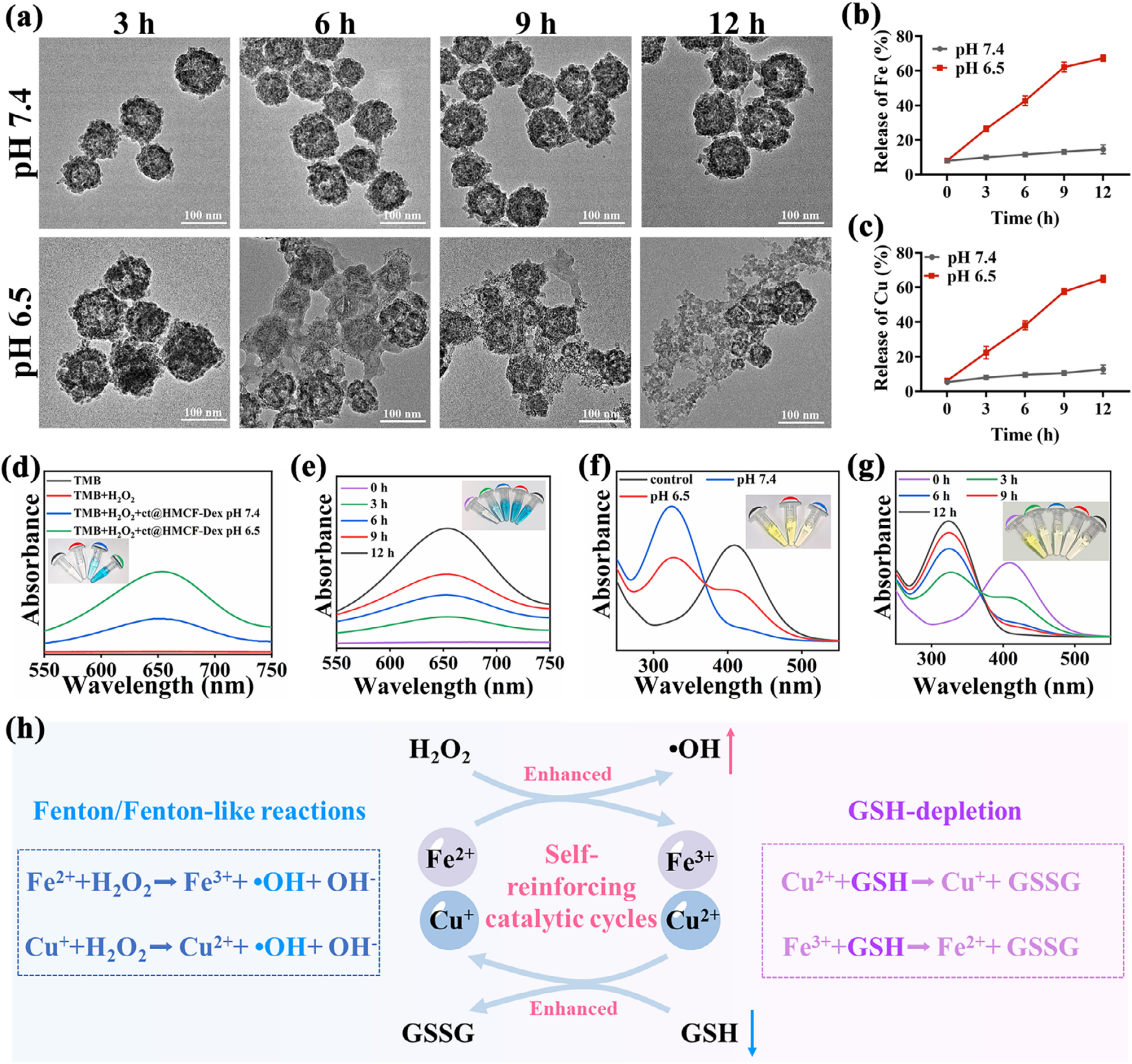
(a) TEM images of the degradation behavior of ct@HMCF‐Dex at different pH values for 3, 6, 9, and 12 h. The cumulative release curves of iron (b) and copper (c) of ct@HMCF‐Dex in PBS under different pH conditions (pH 7.4 and 6.5) (*n* = 3). Error bars represent means ± SD. The pH‐dependent (d) and time‐dependent (e) production of ROS by ct@HMCF‐Dex was determined by the TMB method. The pH‐dependence (f) and time‐dependence (g) ability of ct@HMCF‐Dex nanoparticles to consume GSH was determined by DTNB as an indicator. (h) Schematic representation of the Fenton/Fenton‐like reaction and GSH depletion of the nanoplatform.

The infected wound microenvironment is characterized by local acidosis (pH < 7), a condition resulting from bacterial activity, inflammation, hypoxia, and the build‐up of acidic metabolites [35, 36, 37]. To evaluate the pH‐responsive degradation behavior of ct@HMCF‐Dex, we performed TEM monitoring of nanoparticles incubated in PBS at pH 7.4 and 6.5 for varying durations. The nanoparticles maintained their spherical morphology without significant alteration under neutral conditions (pH 7.4). In contrast, progressive degradation was observed at pH 6.5 with increasing incubation time (Figure 2a), demonstrating the potential of ct@HMCF‐Dex as a degradable nanoplatform designed for the bacterial infection microenvironment. Inspired by this pronounced pH‐dependent biodegradability, we quantitatively analyzed ion release profiles under different pH conditions. As expected, acidic conditions significantly enhanced the release of Fe (Figure 2b) and Cu (Figure 2c) ions from ct@HMCF‐Dex. After 12 h in acidic PBS (pH 6.5), cumulative release reached 67.30% for Fe and 64.92% for Cu, while remaining below 15% for both ions under neutral conditions. These results strongly support that pH‐responsive metal ion release represents a prerequisite and enabling condition for ferroptosis‐ and cuproptosis‐like pathway. The ROS generated by Fe^2+^ and Cu^+^ were assessed using the TMB colorimetric assay. Figure 2d shows that exposure to H_2_O_2_ in a neutral medium lead to an increase in absorbance at 652 nm, and the value observed at pH 6.5 is even higher. This effect is likely due to the acid‐induced degradation of ct@HMCF‐Dex, which releases metal ions that boost Fenton/Fenton‐like reactions. Figure 2e illustrates that the absorbance of oxidized TMB at 652 nm gradually increases over time at pH 6.5. Moreover, electron paramagnetic resonance (EPR) spectroscopy with DMPO as a spin trap was employed to directly detect •OH. Incubation at pH 6.5 resulted in a markedly enhanced EPR signal (Figure S5), confirming •OH generation. Notably, GSH, which is commonly abundant in bacteria, may deplete ROS and protect bacteria from oxidative stress [38]. Therefore, we further examined the GSH depletion capability of copper and iron ions using DTNB as a probe (DTNB can detect thiol groups). As seen in Figure 2f, co‐incubation of ct@HMCF‐Dex with GSH at pH 6.5 led to a significant decrease in DTNB signal, demonstrating efficient GSH depletion by metal ions released from the nanoplatform under acidic conditions. In Figure 2g, the consumption of GSH by ct@HMCF‐Dex is time‐dependent, which may be attributed to the redox interaction between the released Cu^2+^/Fe^3+^ and GSH. Figure 2h illustrates the catalytic mechanism of ct@HMCF‐Dex. Under acidic conditions, pH‐sensitive nanoplatforms composed of mixed‐valence metal elements undergoes degradation, releasing iron ions (Fe^2+^/Fe^3+^) and copper ions (Cu^+^/Cu^2+^). In this system, Fe^2+^ and Cu^+^ mainly drive the Fenton and Fenton‐like reactions to generate •OH, while Cu^2+^ and Fe^3+^ are responsible for depleting endogenous GSH. The reversible valence transitions (Cu^2+^ to Cu^+^ and Fe^2+^ to Fe^3+^) enable the continuous regeneration of these reactive species, thereby sustaining catalytic cycles to amplify oxidative stress and enhance antibacterial efficacy.

### 2.3 Antimicrobial Effect of Ct@HMCF‐Dex In Vitro

**FIGURE 3:**
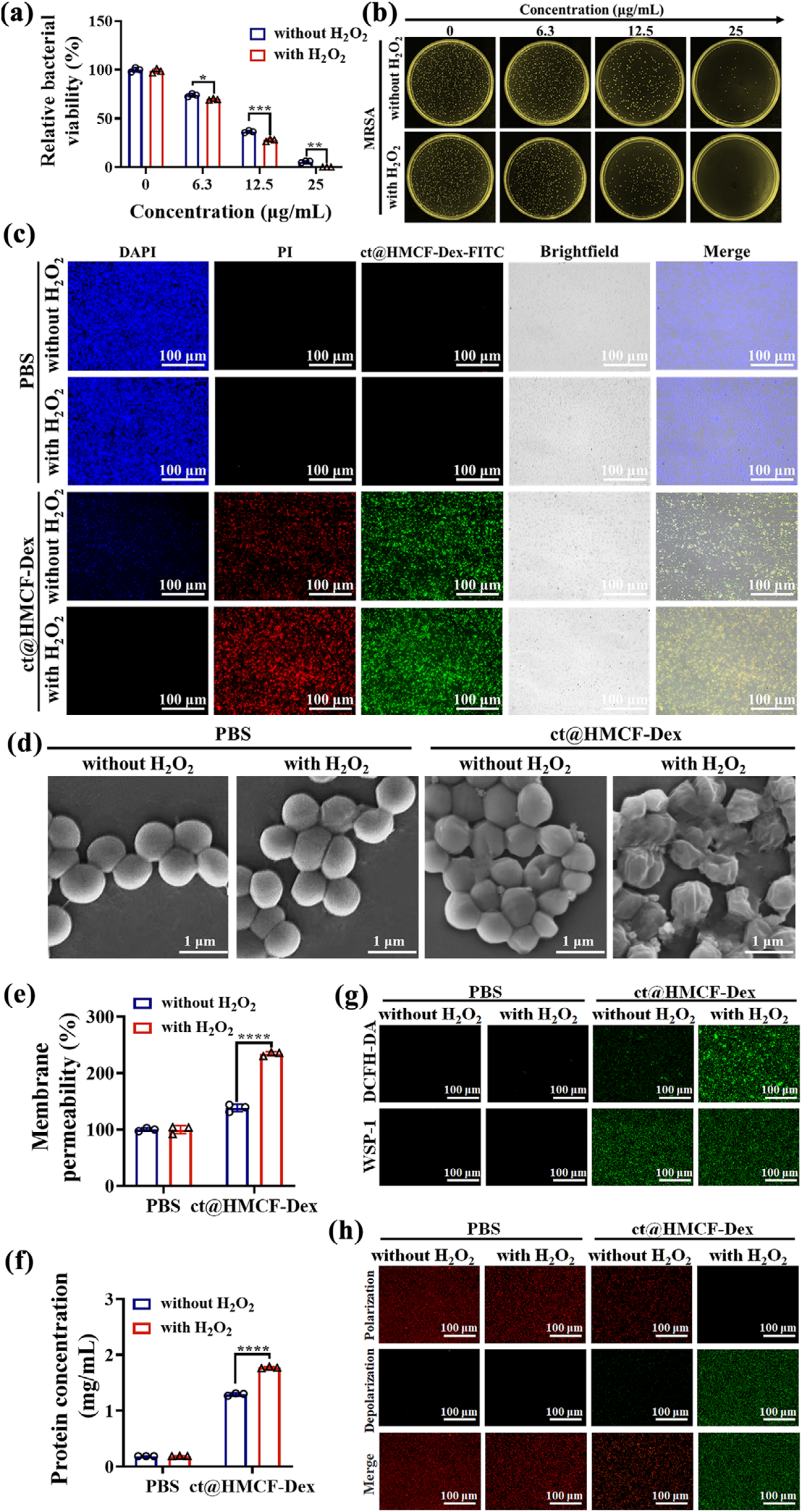
Bacterial survival rate (a) and corresponding bacterial colonies (b) of MRSA treated with ct@HMCF‐Dex (pH = 6.5). (c) Live/dead fluorescence images of MRSA treated with ct@HMCF‐Dex (pH = 6.5). (d) Morphological observation of MRSA after coincubation with ct@HMCF‐Dex. Membrane permeability (e) and protein leakage (f) of MRSA after different treatment (*n* = 3). (g) Fluorescence images of ROS, H_2_S in MRSA after different treatments. (h) The membrane potential of MRSA after different treatments. Error bars represent means ± SD. Differences between groups were tested using one‐way ANOVA followed by Tukey's multiple comparisons test. ^*^ *p* < 0.05, ^**^ *p* < 0.01, ^***^ *p* < 0.001, ^****^ *p* < 0.0001.

The antibacterial performance of ct@HMCF‐Dex against MRSA and *P. aeruginosa* was evaluated using a plate count assay. Under acidic conditions (pH 6.5), the treatment with 200 µM H_2_O_2_ alone showed negligible bactericidal activity (Figure 3b; Figure S6b). In contrast, both ct@HMCF‐Dex and ct@HMCF‐Dex + H_2_O_2_ both exhibited remarkable antibacterial effects, with the antibacterial rates against MRSA being 94.31% and 99.86% (Figure 3a), and against *P. aeruginos*a being 95.32% and 99.89% (Figure S6a), respectively. Under neutral conditions (pH 7.4), the antibacterial efficiency of the ct@HMCF‐Dex and ct@HMCF‐Dex + H_2_O_2_ treatment groups decreased, with the antibacterial rates against MRSA being 75.36% and 90.49% (Figure S7a), and against *P. aeruginosa* being 90.78% and 96.11% (Figure S8a). These findings indicated that the nanoplatform was more effective in acidic environments, which may be due to its accelerated release of more metal ions and the enhancement of Fenton/Fenton‐like reactions. Additionally, ct@HMCF‐Dex exhibited photothermal properties. As demonstrated in Figure S9a, ct@HMCF‐Dex (25 µg/mL) reached 44.3°C after 10 min of near infrared (NIR) irradiation (0.5 W/cm^2^), which was sufficient for low‐temperature photothermal antimicrobial therapy [39]. When combined with NIR irradiation, the antibacterial efficacy of ct@HMCF‐Dex + H_2_O_2_ achieved 99.99% inhibition rates against both MRSA and *P. aeruginosa* (Figure S10), demonstrating synergistic effects between the nanoplatform and PTT.

Further comparisons of the antibacterial effects of HMCF, ct@HMCF, and ct@HMCF‐Dex at the same concentration (25 µg/mL) under acidic conditions in the presence of H_2_O_2_ were conducted (Figure S11). The results showed that ct@HMCF‐Dex hardly formed colonies, while the antibacterial activity of ct@HMCF was 86.34%, and that of HMCF was the poorest at 36.47%. Bacteria widely express non‐enzymatic lectins, a class of proteins capable of specifically recognizing and binding carbohydrates [40]. Therefore, carbohydrates exhibiting high affinity for these bacterial lectins can serve as ideal targeting moieties [41]. Dextran, a non‐toxic polysaccharide, has been shown to display high affinity for lectins on bacterial surfaces [42, 43]. Building upon this concept, we modified the material by grafting dextran to enhance its interaction with bacteria. Concurrently, the loaded citric acid facilitates the release of metal ions. The synergistic action of these two features is believed to contribute to the superior bactericidal performance of ct@HMCF and ct@HMCF‐Dex. To verify this efficacy, live/dead staining assays were performed on MRSA and *P. aeruginosa*. As shown in Figure 3c and Figure S6c, the control and H_2_O_2_‐only treatment groups primarily exhibited blue fluorescence. In contrast, treatment with ct@HMCF‐Dex, particularly in combination with H_2_O_2_, resulted in prominent red fluorescence, indicating extensive bacterial death.

Furthermore, to confirm the targeting capability conferred by the dextran modification, FITC was conjugated to ct@HMCF‐Dex for a fluorescence tracking experiment. Both the Live/Dead staining assays and flow cytometry analysis results revealed distinct green fluorescence in both the ct@HMCF‐Dex group and the ct@HMCF‐Dex + H_2_O_2_ group, confirming a specific interaction between the material and the bacteria (Figure 3c; Figures S6c and S12). Additionally, we further evaluated the bacterial targeting of Dex‐modified ct@HMCF (ct@HMCF‐Dex) by SEM after co‐incubation with bacteria. Compared to unmodified ct@HMCF, clear adhesion to bacterial surfaces was observed in the ct@HMCF‐Dex group (Figure S13), confirming its ability to target and bind bacteria.

The surface morphology of MRSA and *P. aeruginosa* under different treatment conditions was observed by SEM. As shown in Figure 3d and Figure S14a, the bacteria exposed only to H_2_O_2_ maintained a complete and smooth surface, similar to the control group, indicating that H_2_O_2_ (200 µM) did not produce a significant antibacterial effect. In contrast, the bacteria treated with ct@HMCF‐Dex or ct@HMCF‐Dex + H_2_O_2_ showed varying degrees of damage, among which the ct@HMCF‐Dex + H_2_O_2_ treatment caused severe disruption of the cell structure. The morphological deterioration was further confirmed by membrane permeability assays. After treatment with ct@HMCF‐Dex + H_2_O_2_, the membrane permeability of MRSA (Figure 3e) and P*. aeruginosa* (Figure S14b) increased to 2.34‐ and 3.49‐fold that of the control group, respectively. Additionally, the integrity of the membrane structure was assessed by measuring the leakage of intracellular proteins using a bicinchoninic acid (BCA) assay. As shown in Figure 3f and Figure S14c, when ct@HMCF‐Dex was treated alone, only slight protein leakage occurred. However, ct@HMCF‐Dex + H_2_O_2_ increased protein release by approximately 1.37‐fold in MRSA and 1.67‐fold in *P. aeruginosa*. The generation of ROS was monitored using 2′,7'‐Dichlorodihydrofluorescein diacetate (DCFH‐DA) fluorescence staining. Both ct@HMCF‐Dex and ct@HMCF‐Dex + H_2_O_2_ treatments induced strong green fluorescence, with the ct@HMCF‐Dex + H_2_O_2_ group exhibiting relatively stronger green fluorescence (Figure 3g; Figure S14d). This suggests that the intrinsic ROS‐generating capacity of ct@HMCF‐Dex through Fenton/Fenton‐like reactions. Moreover, the H_2_S‐releasing capacity of ct@HMCF‐Dex was quantitatively assessed using the Washington State Probe (WSP‐1). Under acidic conditions (pH 6.5), obvious green fluorescence signals were observed in both ct@HMCF‐Dex and ct@HMCF‐Dex+H_2_O_2_ treatment groups (Figure 3g; Figure S14d), confirming pH‐dependent H_2_S generation. No detectable signal occurred at neutral pH (Figure S15), demonstrating the ct@HMCF‐Dex functions as an effective H_2_S donor through acid‐triggered decomposition. The released H_2_S establishes a self‐reinforcing cycle. First, it further acidifies the local microenvironment, and then accelerates the Fenton‐like/Fenton‐like reaction mediated by Fe^2+^/Cu^2+^, and reacts with H_2_O_2_ to generate additional •OH, thereby promoting bacterial death [20]. Membrane integrity disruption leads to leakage of intracellular ions and small molecules, collapsing the electrochemical gradient. This membrane depolarization was quantified using the potentiometric dye 3,3'‐diethyloxacarbocyanine iodide (DiOC_2_(3)) [44]. As depicted in Figure 3h and Figure S14e, both the control and H_2_O_2_‐treated groups showed pronounced red fluorescence, indicating that the cell membranes remained in a polarized state. In contrast, bacteria exposed to ct@HMCF‐Dex + H_2_O_2_ emitted strong green fluorescence, suggesting that the transmembrane potential was reduced and the cell membranes were severely damaged.

### 2.4 Antibacterial Mechanism of ct@HMCF‐Dex

**FIGURE 4:**
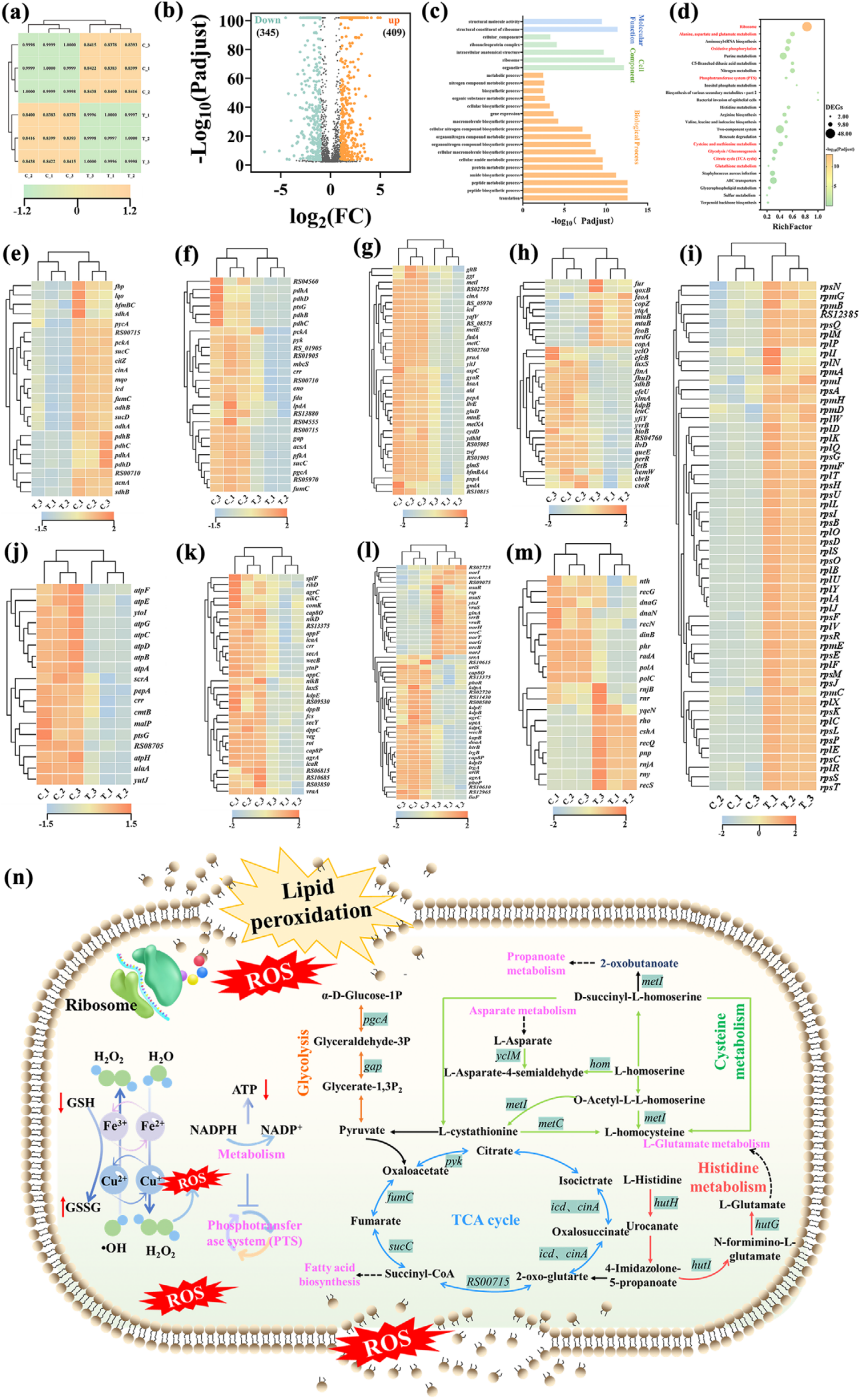
(a) Correlation analysis of blank LB groups and ct@HMCF‐Dex groups (*n* = 3). (b) Volcano plot analyses of total DEGs in MRSA after treatment with blank LB or Cu‐ ct@HMCF‐Dex (*n* = 3). (c) GO enrichment analysis of DEGs (*n* = 3). (d) KEGG enrichment analysis of DEGs (*n* = 3). Heat map of genes associated with (e) TCA cycle, (f) glycolysis, (g) amino acid metabolism, (h) iron and copper transport, (i) ribosome, (j) oxidative phosphorylation, (k) QS, (l) two‐component systems, (m) DNA replication and repair and RNA degradation (*n* = 3). (n) Schematic illustration of anti‐extracellular bacterial mechanisms of ct@HMCF‐Dex.

To elucidate the antimicrobial mechanisms of ct@HMCF‐Dex at the molecular level, we conducted transcriptome sequencing (RNA‐seq) of MRSA following treatment. The correlation analysis of Figure 4a and Figure S16 indicated the reliability of the sequencing results. Figure 4b and Figure S17 showed that 754 differentially expressed genes (DEGs) were identified in the gene expression profile of MRSA. Among them, 409 genes were up‐regulated, and 345 genes were down‐regulated. Gene Ontology (GO) enrichment analysis showed that the largest changes in genes quantity were associated with translation, peptide anabolism, and ribosomes. These results indicated that ct@HMCF‐Dex could significantly affect the oxidative stress of MRSA and had a significant effect on the molecular function of MRSA (Figure 4c). The Kyoto Encyclopedia of Genes and Genomes (KEGG) enrichment analysis showed that ct@HMCF‐Dex mainly affected bacterial metabolism, including ribosomes, amino acid metabolism, oxidative phosphorylation, phosphotransferase system, and TCA cycle (Figure 4d).

Subsequently, we further analyzed the specific functions affected by ct@HMCF‐Dex using heat maps. The DEGs related to ribosomes were significantly upregulated in Figure 4i. According to previous studies, ROS‐induced oxidative stress can impair membrane‐associated protein transporters and compromise membrane integrity, which may facilitate the intracellular accumulation of harmful substances and indirectly disrupt ribosomal function [13, 45]. This aligns with our transcriptomic data, which show a significant enrichment of DEGs related to ribosomes, suggesting that ROS likely interferes with ribosomal processes. Excessive copper and iron ions in bacterial cells also impede TCA cycling and induce the accumulation of lipid peroxides, thereby promoting the occurrence of cuproptosis/ferroptosis‐like death in bacteria [46]. Notably, the heatmaps in Figure 4e,f show that a large number of genes related to TCA cycling and glycolysis are downregulated, leading to the occurrence of cuproptosis/ferroptosis‐like death in bacteria. The significant dysregulation of multiple genes related to membrane transport and transmembrane transporters (including those involved in ATP‐binding cassette (ABC) transporters (Figure S18) and ion transporters (Figure 4h). These changes indicate substantial disruption of the bacterial transport machinery. The translocation of copper and iron ions led to copper and iron accumulation through a series of signaling pathways, which in turn promoted bacterial cuproptosis/ferroptosis‐like death [47]. The heatmap in Figure 4h showed that copper ion export regulator genes (such as *copZ* and *copA*) were significantly up‐regulated, the iron homeostasis regulators (e.g., *Fur*) were activated to limit free iron accumulation, while Fe‐S cluster cofactors (*mtaB*, etc.) were significantly down‐regulated. This indicates that there may be a large amount of Fe and Cu ions on the bacterial surface cells, thereby accelerating the occurrence of cuproptosis/ferroptosis‐like death in the bacteria. Methionine is produced by conversion to S‐adenosylmethionine, followed by homocysteine and ultimately cysteine by cystathionine β‐synthase and cystathionine γ‐cleavage enzymes. Figure 4g shows that the genes related to methionine, cysteine, and GSH metabolism are significantly downregulated, resulting in blockage of GSH synthesis, inability to scavenge lipid peroxides, thereby causing the accumulation of lipid peroxides, and subsequently leading to bacterial death. Quorum sensing (QS) is a cell‐to‐cell communication system among bacteria, referring to the growth process of microbial communities. During this process, a small number of bacteria exhibit characteristics that they do not possess originally, such as the ability to adapt to environmental changes (such as the production of virulence factors and the formation of biofilms due to increased population density) [48]. The heatmap in Figure 4k showed that genes related to QS were downregulated, suggesting that ct@HMCF‐Dex may inhibit the QS system of MRSA, thereby weakening the environmental adaptation of MRSA. The two‐component signaling system regulates various physiological activities of bacteria and helps them to adapt to their environment [49]. As expected, most genes encoding virulence factors (such as *agrA* and *agrC)* and genes related to adhesion, autolysis, multi‐drug resistance, and virulence regulation (such as *arlS* and *arlR*) have been significantly downregulated. Figure 4m demonstrated the downregulated expression of genes associated with deoxyribonucleic acid (DNA) replication and repair, along with upregulated expression of ribonucleic acid (RNA) degradation‐related genes. This phenomenon can be attributed to substantial ROS generation, which, under conditions of LPO‐induced oxidative stress, leads to both DNA damage and RNA degradation. The electron transport chain facilitates ATP production through oxidative phosphorylation via a series of electron transfer reactions [50]. The heatmap in Figure 4j showed that genes related to oxidative phosphorylation were downregulated, suggesting that ct@HMCF‐Dex may disrupt the homeostasis of bacterial cells, leading to reduced ATP synthesis and disrupting the energy metabolism of bacteria. The bacterial two‐component system is a signal transduction system consisting of a response regulator that regulates gene expression to modulate the response and a corresponding histidine kinase that detects specific signals [7]. The accumulation of ct@HMCF‐Dex can inhibit the defensive functions of the two‐component system and disrupt iron homeostasis, leading to iron‐induced toxicity in pathogens (Figure 4l). Excessive extracellular ferrous ions (Fe(II)) can inhibit the defense efficacy of the two‐component system, disrupt iron metabolism balance, and ultimately trigger ferroptosis [51].

In summary, we proposed the antibacterial mechanism of ct@HMCF‐Dex (Figure 4n). First, ct@HMCF‐Dex targets bacteria through dextran‐mediated binding. Under acidic conditions, it releases Fe^3+^ and Cu^2+^, which deplete intracellular GSH and catalyze the generation of •OH from H_2_O_2_. This process disrupts the bacterial outer membrane and internal structures, leading to cytoplasmic leakage and GSH deficiency. The accumulation of ROS, Fe^2+^, and Cu^+^ within the bacteria induces severe LPO, ultimately causing irreversible cuproptosis/ferroptosis‐like cell death. The metabolic pathways involving glucose, lipids, and cysteine, as well as genetic processes, were disrupted, indicating systemic bacterial dysfunction. These results demonstrate that ct@HMCF‐Dex efficiently and rapidly induces bacterial death, exhibiting features resembling cuproptosis and ferroptosis. Additionally, ct@HMCF‐Dex suppressed the bacterial QS system, further impairing energy metabolism and reproduction.

**FIGURE 5:**
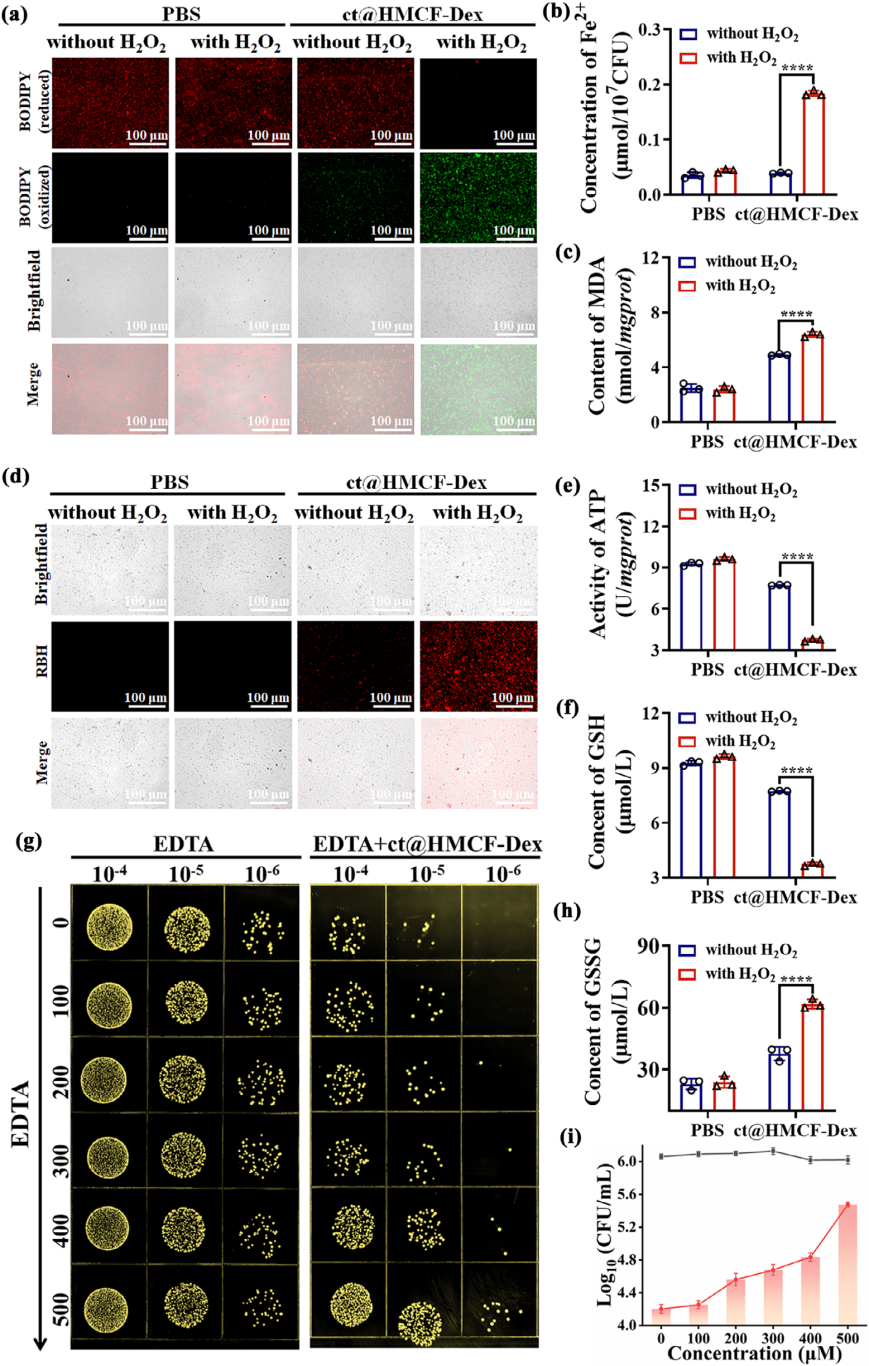
(a) Fluorescence images of LPO levels after different treatments of MRSA. Changes of Fe^2+^ (b), MDA (c), ATP (e), GSH (f), and GSSG (h) contents in MRSA after different treatments (*n* = 3). (d) Fluorescence images of copper ion levels after different treatments of MRSA. Colonization (g) and corresponding survival (i) of MRSA after co‐incubation of different concentrations of EDTA with ct@HMCF‐Dex (*n* = 3). Error bars represent means ± SD. Differences between groups were tested using one‐way ANOVA followed by Tukey's multiple comparisons test. ^****^ *p* < 0.0001.

To validate the transcriptome sequencing results, we investigated whether ct@HMCF‐Dex could induce cuproptosis/ferroptosis‐like bacterial death in vitro. As shown in Figure 5a and Figure S19a, MRSA and *P. aeruginosa* treated with ct@HMCF‐Dex + H_2_O_2_ exhibited the most severe LPO, whereas H_2_O_2_ alone or ct@HMCF‐Dex alone caused minimal effects. Furthermore, the combination of ct@HMCF‐Dex and H_2_O_2_ significantly increased intracellular Fe^2+^ (Figure 5b; Figure S19b) and copper ion (Figure 5d; Figure S19d) accumulation. A hallmark of both ferroptosis‐like and cuproptosis‐like cell death is the accumulation of LPO products [2, 13]. To quantify this effect, we measured malondialdehyde (MDA), a key byproduct of LPO, in bacteria treated with ct@HMCF‐Dex. As shown in Figure 5c and Figure S19c, MRSA and *P. aeruginosa* exposed to ct@HMCF‐Dex + H_2_O_2_ exhibited 1.30‐ and 1.77‐fold higher MDA levels, respectively, compared to treatment with ct@HMCF‐Dex alone. Concurrently, GSH levels in MRSA were significantly depleted (Figure 5f; Figure S19f), while the oxidized glutathione (GSSG)/GSH ratio increased markedly (Figure 5h; Figure S19h), indicating severe redox imbalance. Furthermore, ATP production was substantially reduced (Figure 5e; Figure S19e). Taken together, these results demonstrate that ct@HMCF‐Dex + H_2_O_2_ induces ferroptosis/cuproptosis‐like bacterial death through GSH depletion, exacerbated LPO, and disrupted energy metabolism.

To further validate these findings, we employed ethylenediaminetetraacetic acid (EDTA), an inhibitor of both ferroptosis‐like and cuproptosis‐like cell death [52]. Chelation of iron and copper ions by EDTA significantly attenuated the antibacterial efficacy of ct@HMCF‐Dex + H_2_O_2_, demonstrating that free iron and copper ions are essential for its bactericidal activity (Figure 5g,i; Figure S19g,i). Additionally, other metal ion chelators, such as L‐glutamate (L‐Glu), adenosine diphosphate (ADP), and adenosine triphosphate (ATP), were found to be able to regulate the antibacterial efficacy of ct@HMCF‐Dex + H_2_O_2_ (Figures S20–S22). Similarly, antioxidants including GSH, vitamin C, Ferrostatin‐1, and tetrathiomolybdate (TTM) also impeded the antibacterial performance of ct@HMCF‐Dex + H_2_O_2_ (Figures S23–S26). These results demonstrate that: (1) metal ion sequestration directly diminishes antibacterial efficacy by restricting iron and copper availability, and (2) exogenous antioxidants confer bacterial protection by counteracting oxidative processes characteristic of ferroptosis/cuproptosis‐like death pathways. Collectively, these findings provide additional evidence highlighting the essential involvement of unbound iron and copper ions in triggering ferroptosis/cuproptosis‐like death pathways in bacteria.

### 2.5 In Vitro Antibiofilm Activity

**FIGURE 6:**
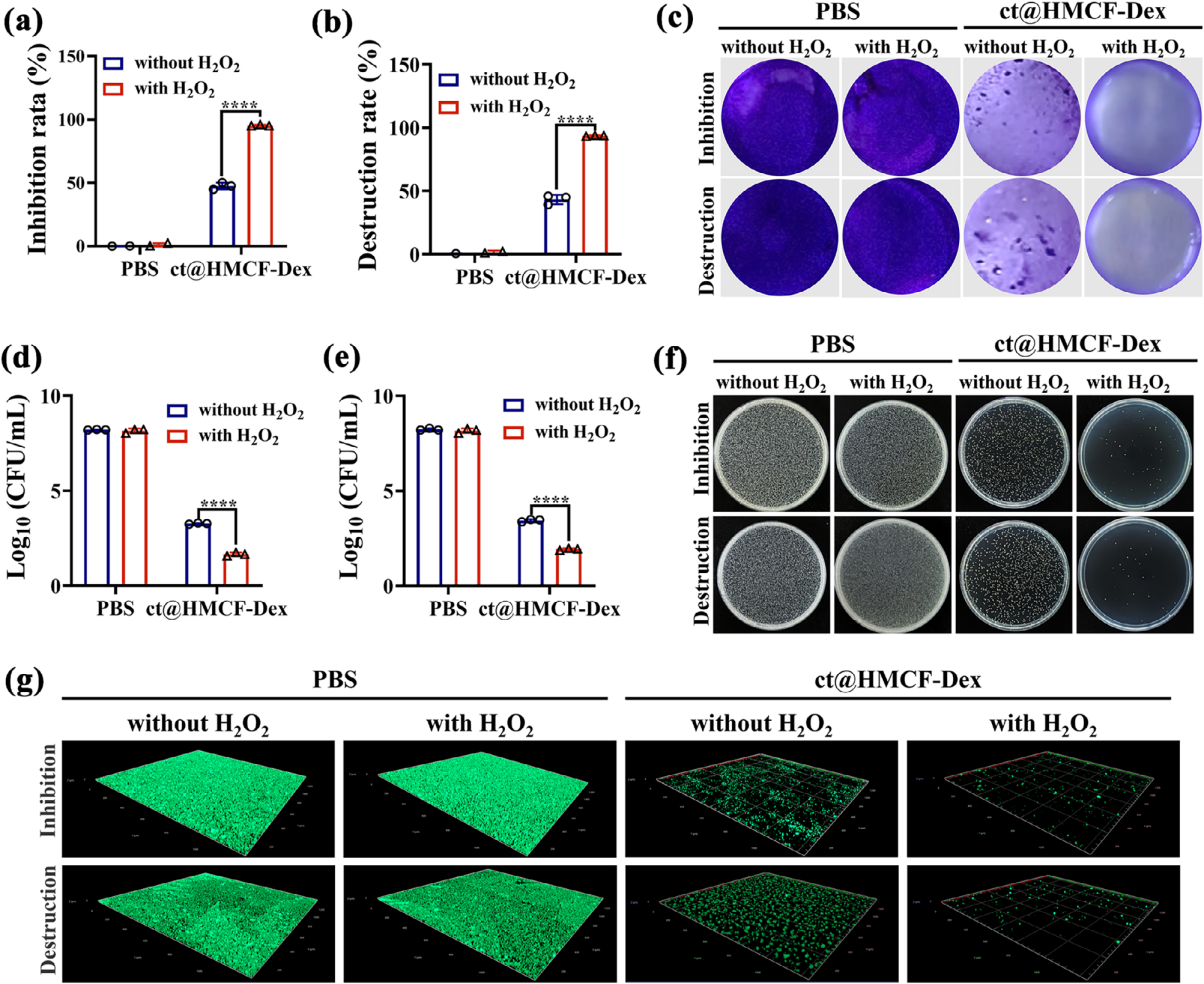
Inhibition rate (a) and destruction rate (b) of ct@HMCF‐Dex against MRSA biofilms and corresponding crystal violet‐stained photographs (c) (*n* = 3). CFU counts of MRSA biofilm inhibition (d) and disruption (e) after different treatments (*n* = 3). Bacterial colonies (f) and fluorescence images (g) of MRSA biofilm inhibition and disruption after different treatments. Error bars represent means ± SD. Differences between groups were tested using one‐way ANOVA followed by Tukey's multiple comparisons test. ^****^ *p* < 0.0001.

When bacteria secrete extracellular polymeric substances (EPS, including polysaccharides, proteins, and DNA), bacterial biofilms are formed [53]. These biofilms can severely impede wound healing and delay the recovery process in bacterial infectious pneumonia. Based on the aforementioned transcriptomic results, we know that ct@HMCF‐Dex inhibits the bacterial QS system, which is crucial for regulating biofilm formation and maintaining biofilm structure. To evaluate whether ct@HMCF‐Dex has the ability to disrupt biofilms and prevent their formation, mature biofilms of MRSA and *P. aeruginosa* were treated with PBS (control), H_2_O_2_, ct@HMCF‐Dex, or ct@HMCF‐Dex + H_2_O_2_, followed by crystal violet staining to quantify biofilm destruction. As shown in Figure 6c and Figure S27c, the crystal violet intensity markedly diminished after treatment with ct@HMCF‐Dex, and the effect of ct@HMCF‐Dex + H_2_O_2_ was even better. Compared to the PBS (control), the disruption rates of MRSA biofilms treated with H_2_O_2_, ct@HMCF‐Dex, and ct@HMCF‐Dex + H_2_O_2_ were 0.20%, 43.35%, and 93.61%, respectively (Figure 6b). A similar trend was observed for *P. aeruginosa* biofilms, with disruption rates of 0.95%, 50.22%, and 93.39%, respectively (Figure S27b).

In parallel, the inhibition of biofilm formation was evaluated by incubating planktonic bacteria with the same treatment groups. For MRSA, biofilm formation was inhibited by 0.86% with H_2_O_2_, 47.58% with ct@HMCF‐Dex, and 95.31% with ct@HMCF‐Dex + H_2_O_2_ (Figure 6a). Similarly, *P. aeruginosa* biofilm formation was reduced by 12.06%, 61.27%, and 95.30% in the respective treatment groups (Figure S27a). These results were further corroborated by fluorescence imaging using SYBR Green I, where the staining patterns clearly correlated with the extent of biofilm disruption and inhibition (Figure 6g; Figure S27g). Additionally, quantitative spread plate assays (Figure 6d–f; Figure S27d–f) confirmed that the ct@HMCF‐Dex + H_2_O_2_ treatment not only eliminated bacteria within mature biofilms but also suppressed bacterial proliferation in early‐stage biofilms. Collectively, these findings demonstrate that ct@HMCF‐Dex + H_2_O_2_ effectively disrupts biofilm integrity, enhances bactericidal activity, and consequently promotes wound healing and accelerates recovery from bacterial infectious pneumonia.

### 2.6 Biocompatibility and Targeting of ct@HMCF‐Dex

**FIGURE 7:**
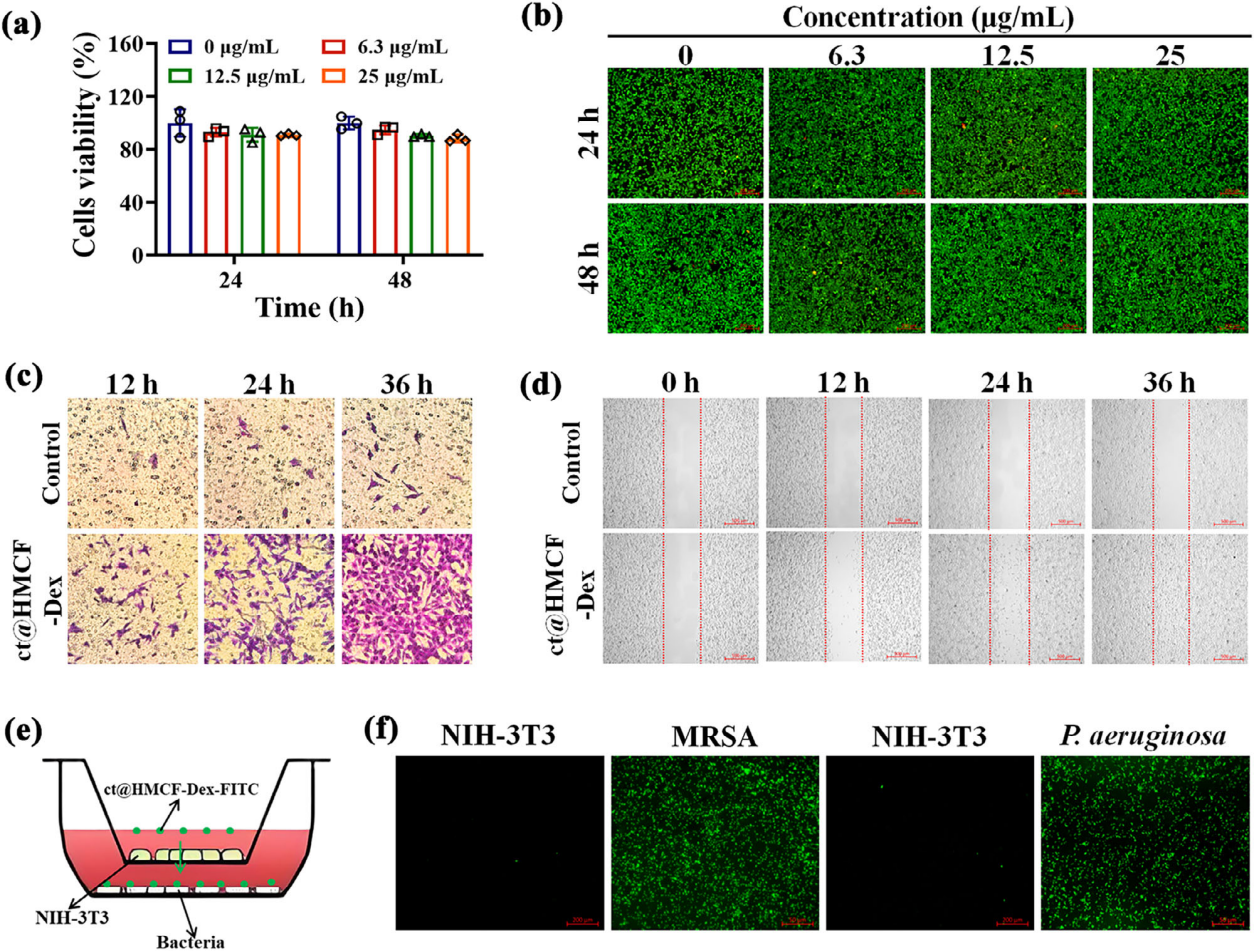
Cytotoxicity (a) of different concentrations of ct@HMCF‐Dex on NIH/3T3 and corresponding cell viability staining (b) (*n* = 3). Error bars represent means ± SD. Migration of different groups of NIH/3T3 cells in Transwell assay (c) and cell scratch assay (d) at different times. (e) Schematic of the Transwell assay for bacterial targeting capacity testing. (f) Inverted fluorescence microscope image by the Transwell experiment.

An ideal antibacterial agent should selectively eradicate bacteria while preserving normal cell function. To evaluate the biocompatibility of ct@HMCF‐Dex, we conducted 3‐(4,5)‐dimethylthiahiazo (‐z‐y1)‐3,5‐diphenytetrazoliumromide (MTT) assays on mouse embryonic fibroblasts (NIH/3T3) after various treatments. As depicted in Figure 7a and Figure S28a, NIH/3T3 and BEAS‐2B cells exposed to ct@HMCF‐Dex (6.3–25 µg/mL) maintained viability over 85%, confirming its excellent cytocompatibility. This result was further supported by live/dead cell staining (Figure 7b; Figure S28b). A concentration of 25 µg/mL was chosen to study the effect of laser irradiation duration. Under low power density (0.5 W/cm^2^), the PBS control group showed negligible changes in cell viability over time, whereas the ct@HMCF‐Dex group experienced a gradual decline, yet still preserved 80% viability after 10 min of exposure (Figure S29). Additionally, cytotoxicity assessments at 24 and 48 h post‐irradiation (10 min) revealed that NIH/3T3 cells survival remained above 85% (Figure S30a,d), further reinforcing the biosafety of ct@HMCF‐Dex. In vivo safety assessment in healthy mice further confirmed the biocompatibility of ct@HMCF‐Dex. As illustrated in Figure S31, while the positive control (pure water) and negative control (PBS) showed expected hemolysis rates of 100% and 0%, respectively, ct@HMCF‐Dex demonstrated exceptional blood compatibility with hemolysis rates consistently below 1% across the tested concentration range (6.3–25 µg/mL). Importantly, these values were significantly lower than the 5% safety threshold established by both the International Organization for Standardization (ISO) and the American Society for Testing and Materials (ASTM), thereby validating its suitability for subsequent in vivo therapeutic applications. Cell migration plays a vital role in wound repair. We investigated the effect of ct@HMCF‐Dex on the migration of NIH/3T3 cells through scratch assays and Transwell migration assays. As illustrated in Figure 7c,d and Figure S32, ct@HMCF‐Dex treatment significantly enhanced cell migration compared to the control, suggesting its potential to facilitate wound healing. To characterize the target selectivity of ct@HMCF‐Dex in physiologically relevant conditions, we used Transwell chambers to mimic an infection microenvironment. NIH/3T3 cells were seeded in the upper chamber while bacteria occupied the lower chamber. FITC‐labeled ct@HMCF‐Dex was introduced into the upper well, and pictures were taken using a fluorescence microscope at the end of the incubation (Figure 7e). As shown in Figure 7f, fluorescence imaging showed obvious green fluorescence aggregation in the bacteria, while no similar phenomenon was observed in the NIH/3T3 cells. These results confirmed that ct@HMCF‐Dex preferentially binds to bacteria, which may be due to the dextran coating on the surface of ct@HMCF‐Dex, which may interact with lectins on the bacterial outer membrane through its glycan units [54].

### 2.7 Antibacterial Effects of ct@HMCF‐Dex In Vivo

Based on the excellent in vitro antibacterial activity and clarified mechanism, we developed two MRSA infection models to validate ct@HMCF‐Dex' in vivo efficacy, beginning with an acute pulmonary infection model established through tracheal instillation MRSA. In severe lower respiratory tract infections, the lungs of patients typically form a thick layer of mucus barrier. Additionally, bacteria secrete EPS composed of lipopolysaccharides, nucleic acids, and proteins. This EPS creates an intricate, 3D matrix that obstructs the penetration of nanomaterials into lung tissue, ultimately reducing their bactericidal effectiveness [55, 56]. To maximize the antibacterial effect, the nanomaterials must rapidly penetrate the mucus barrier. We examined the penetration capacity of ct@HMCF‐Dex in an artificially simulated mucus environment. As depicted in Figure S33a, ct@HMCF‐Dex can rapidly penetrate the mucus layer. The degree of penetration is quantified by measuring the absorbance of the gelatin layer at 595 nm after 3 h. The results show that the penetration speed of ct@HMCF‐Dex is comparable to that of PBS (Figure S33b).

Furthermore, we evaluated the binding interaction between ct@HMCF‐Dex and mucin through a mucin aggregation assay. Using a mucin concentration ranging from 0.1% to 0.5% by mass/volume to simulate the mucin concentration range from normal lung mucus to severe pneumonia. The binding of mucin with ct@HMCF‐Dex was quantified using the fluorescent marker FITC. The results showed that at a mucin concentration of 0.3% and 0.5%, approximately 22% of the mucin was bound to the nanoparticles (Figure S33c). Additionally, an in vitro mucus model was established in a Transwell system, where artificial mucus was cultured in the upper chamber, and ct@HMCF‐Dex‐FITC was added. After various incubation periods, the fluorescence intensity measured in the receiver compartment confirmed that the nanoparticle successfully penetrated the mucus layer within 10 h (Figure S33d). The penetration rate index (Papp) was calculated. Figure S33e shows that the Papp of ct@HMCF‐Dex‐FITC reached 116.91 × 10^−1^ cm/s after 1 h. We speculate that the nearly neutral zeta potential of ct@HMCF‐Dex minimized the interactions with mucin, allowing the nanoparticles to diffuse rapidly through the mucus network [57]. These characteristics facilitate the particles in effectively penetrating mucus after atomization inhalation into the lungs and reaching the surface of biological membranes to exert their antibacterial effects.

**FIGURE 8:**
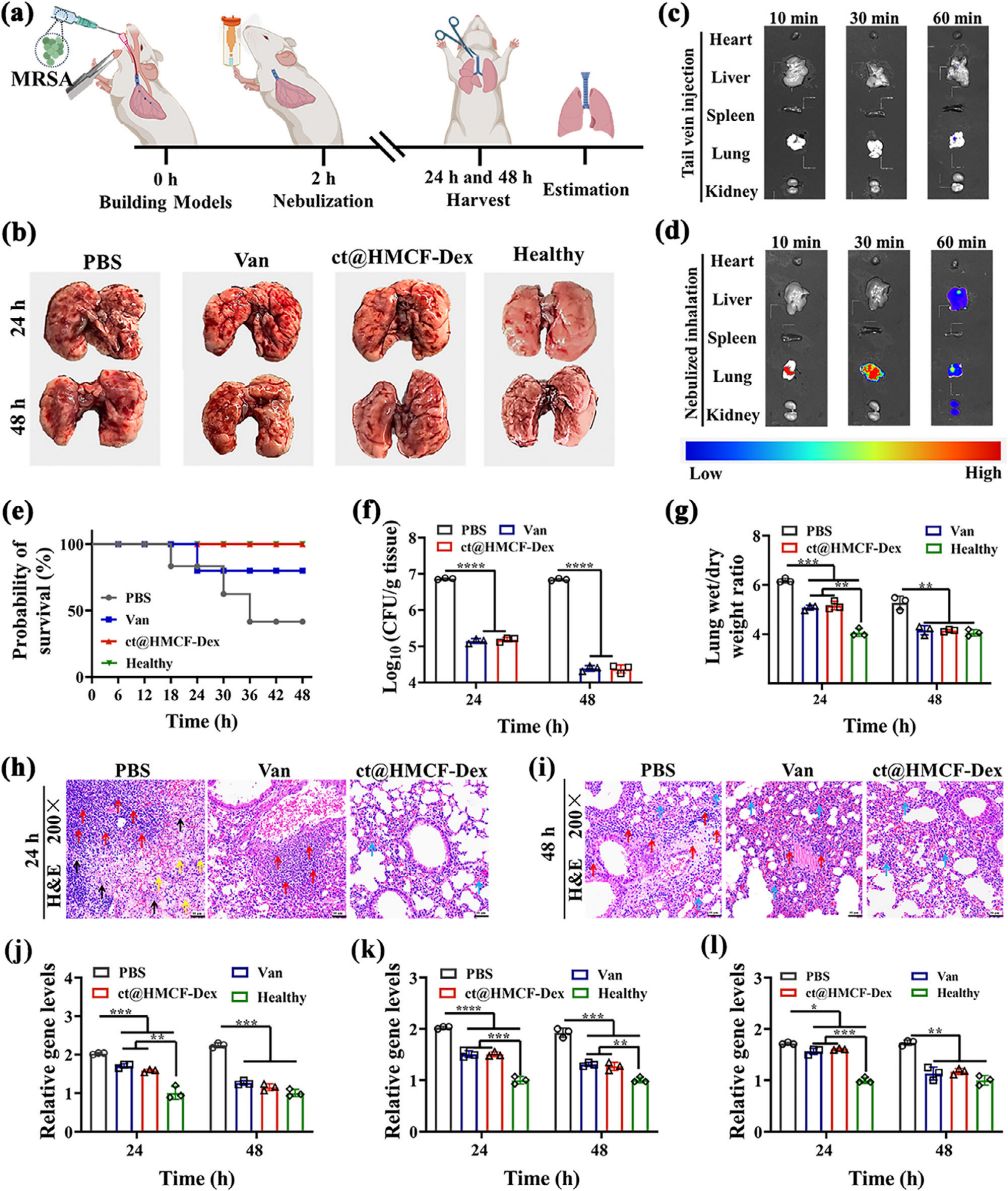
(a) Schematic diagram of the process of constructing and treating MRSA lung infections. (b) Photographs of mouse lungs after different treatments. Fluorescence distribution in vivo at different times after intravenous injection (c) and nebulized inhalation (d). (e) Survival of mice after different treatments. (f) Bacterial CFU counts in the lung tissue of mice after different treatments (*n* = 3). (g) Wet/dry ratios of mouse lung tissues after 24 and 48 h of different treatments (*n* = 3). H&E staining images of mouse lung tissue after 24 h (h) and 48 h (i) of different treatments. Relative expression of IL‐6 (j), TNF‐α (k), and IL‐1β (l) in mouse lung tissues after 24 and 48 h of different treatments (*n* = 3). Error bars represent means ± SD. Differences between groups were tested using one‐way ANOVA followed by Tukey's multiple comparisons test. ^*^ *p* < 0.05, ^**^ *p* < 0.01, ^***^ *p* < 0.001, ^****^ *p* < 0.0001.

Intravenous and inhalation administration are commonly used in the clinical management of lung infections. For intravenous drug delivery, the drug can only reach the infected site through systemic circulation, and this way of drug delivery has the problems of slow drug delivery and high toxicity. However, inhalation drug delivery can reach the lungs through the respiratory system, which not only can reach the infected site quickly but also has lower drug loss and toxicity [4]. To verify whether the inhaled drug can reach the lungs quickly, real‐time fluorescence imaging was performed to characterize the treated mice. The results in Figure 8c,d showed that some of the ct@HMCF‐Dex‐Cy5.5 reaches the lungs within 10 min by inhalation and continued to accumulate over time. In contrast, when the drug was administered by intravenously, ct@HMCF‐Dex did not reach the infected site without circulation after 1 h, and treatment was not possible in the short term. These results suggested that the inhalation‐based treatment method can quickly and efficiently deliver the material to the lungs.

Building upon the demonstrated mucus‐penetrating capability, we systematically evaluated the antimicrobial performance of ct@HMCF‐Dex in a murine pulmonary infection model. Mice were infected with MRSA via tracheal instillation. Two hours later, ct@HMCF‐Dex was delivered using nebulization therapy. Lung tissues were harvested at 24 and 48 h post‐treatment (Figure 8a). Tissue homogenates were prepared and the bacterial burden was quantified by the plate spreading method. As depicted in Figure 8f and S34, the bactericidal effect of ct@HMCF‐Dex was comparable to that of the vancomycin (Van) positive control group. Moreover, lung images revealed that mice in the PBS negative treatment group exhibited marked hemorrhage and edema, while those in the ct@HMCF‐Dex group showed minimal pathological changes (Figure 8b). Survival analysis further highlighted the efficacy of this therapy. 58.33% of MRSA‐infected mice in the PBS group succumbed, while the ct@HMCF‐Dex‐treated group achieved a 100% survival rate (Figure 8e). Additionally, the body weight of mice in the PBS group decreased from 20.37 to 18.46 g, and their core body temperature decreased from 37.57°C to 32.53°C, whereas those treated with ct@HMCF‐Dex maintained higher levels (averaging 19.70 g) and a higher body temperature (36.47°C) after 48 h (Figure S35a,b). To investigate the targeting of ct@HMCF‐Dex against bacteria in vivo, the metabolism and distribution of Cy5.5‐labeled ct@HMCF‐Dex in the lungs of mice with pneumonia were systematically analyzed using small animal in vivo imaging. Both the MRSA‐induced pneumonia mouse and the healthy control mouse were injected with the same dose of ct@HMCF‐Dex‐Cy5.5 via the tail vein. Based on the time‐dependent biodistribution of the nanoparticles, the mice were executed at 4, 8, 12, and 24 h post‐injection, and the major organs were harvested for ex vivo fluorescence imaging. As shown in Figure S36, nanoparticle fluorescence accumulation was significantly higher in the inflamed lungs compared to healthy lung tissues, demonstrating the nanoparticles' ability to specifically target pneumonia in vivo. Furthermore, ex vivo imaging of healthy mice revealed that ct@HMCF‐Dex primarily accumulated in the liver and kidneys, with minimal presence in the lungs. The fluorescence intensity gradually weakened over time, and the same phenomenon occurred in the liver and kidney after 24 h (Figure S36), which may be a result of nanoparticle metabolism and excretion through the liver and kidney.

To further evaluate treatment efficacy, we analyzed lung tissue morphology using H&E staining. At 24 h post‐treatment, PBS‐treated mice exhibited severe pulmonary damage, characterized by protein‐rich mucus accumulation in alveolar spaces (yellow arrows), widespread inflammatory cell infiltration (red arrows), and necrotic regions with nuclear fragmentation (black arrows). In contrast, the ct@HMCF‐Dex group showed partial alveolar atrophy, thickened septal walls, and hyperplasia of alveolar epithelial cells (blue arrows). The Van group displayed indistinct alveolar boundaries, collapsed airspaces (blue arrows), residual proteinaceous exudate (yellow arrows), and persistent inflammatory infiltration (red arrows) (Figure 8h). At 48 h post‐treatment, the pathological state in the treatment groups showed improvement compared to the PBS control. In the PBS group, persistent pulmonary damage was evident, including partial alveolar atrophy, markedly thickened septal walls, and pronounced hyperplasia of alveolar epithelial cells (blue arrows), accompanied by sustained, widespread inflammatory infiltration (red arrows). The ct@HMCF‐Dex group displayed similar structural changes, such as partial alveolar collapse and thickened alveolar walls with epithelial hyperplasia (blue arrows). However, bronchial epithelial cells remained well‐organized, and no inflammatory infiltration was detected. Similarly, the Van group showed alveolar atrophy and wall thickening with epithelial hyperplasia (blue arrows), but bronchial architecture was preserved, and only minimal inflammatory cells were present (red arrows) (Figure 8i). To further evaluate therapeutic efficacy, we observed that ct@HMCF‐Dex‐treated mice exhibited the lowest lung wet/dry weight ratio at 48 h, suggesting only mild pulmonary edema (Figure 8g). To assess immunomodulatory effects, we quantified inflammatory cytokines in lung tissue homogenates. As shown in Figure 8j–l, pro‐inflammatory cytokines (interleukin‐6 (IL‐6), tumor necrosis factor‐α (TNF‐α), and interleukin‐1β (IL‐1β)) were markedly elevated in the model group compared to healthy controls, whereas ct@HMCF‐Dex treatment significantly attenuated these inflammatory markers. At 48 h post‐treatment, leukocyte subsets (including lymphocytes, monocytes, and neutrophils) remained elevated in PBS‐treated mice but returned to near‐baseline levels in the ct@HMCF‐Dex group (Figure S37). Notably, no significant alterations were detected in erythrocyte counts, hemoglobin levels, or other hematological parameters (Figure S37). Furthermore, biochemical indicators of liver and kidney function in mice (including alanine aminotransferase (ALT), aspartate aminotransferase (AST), blood urea nitrogen (BUN), and creatinine (CR)) showed no significant changes (Figure S38). H&E‐stained sections of major organs (heart, liver, spleen, and kidneys) revealed no pathological abnormalities in ct@HMCF‐Dex treated mice, comparable to healthy controls (Figure S39). These findings collectively demonstrate the in vivo safety of ct@HMCF‐Dex.

**FIGURE 9:**
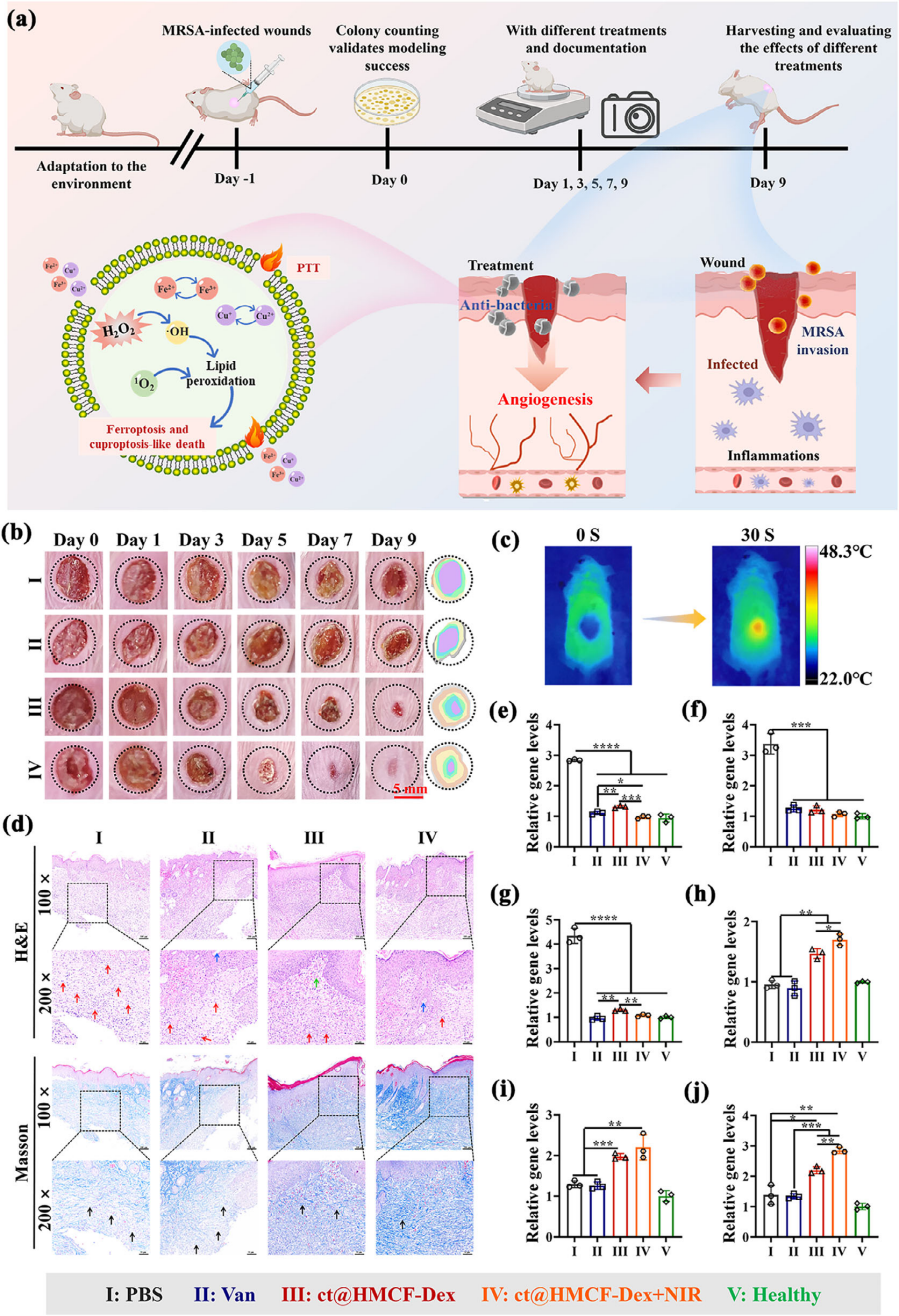
(a) Schematic diagram of the process of constructing and treating MRSA wound infections and in vivo sterilization. (b) Photographs of wound changes in mice after different treatments for different days. (c) Thermogram of mice with the treatment of ct@HMCF‐Dex+NIR. (d) Images of H&E staining and Masson staining of mouse skin tissue after 9 days of different treatments. Relative expression of IL‐6 (e), TNF‐α (f), IL‐1β (g), bFGF (h), COL‐III (i), and VEGF (j) in mouse skin tissues after 9 days of different treatments (*n* = 3). Error bars represent means ± SD. Differences between groups were tested using one‐way ANOVA followed by Tukey's multiple comparisons test. ^*^ *p* < 0.05, ^**^ *p* < 0.01, ^***^ *p* < 0.001, ^****^ *p* < 0.0001.

To evaluate the therapeutic efficacy of ct@HMCF‐Dex on wound healing, a skin wound model infected with MRSA was established (Figure 9a). We examined the in vivo photothermal performance of ct@HMCF‐Dex under 808 nm laser irradiation. Remarkably, within just 30 s of NIR exposure, the wound temperature in the ct@HMCF‐Dex + NIR group rapidly reached 45.6°C (Figure 9c), while the PBS control showed negligible temperature change (Figure S40). The treatment outcomes demonstrated superior wound closure, with the ct@HMCF‐Dex + NIR group achieving near‐complete healing by day 9 (Figure 9b). Quantitative analysis revealed wound closure rates of 54.05% (PBS), 55.36% (Van), 94.34% (ct@HMCF‐Dex alone), and 98.85% (ct@HMCF‐Dex + NIR) (Figure S41d). Correspondingly, bacterial viability on day 9 was dramatically reduced to 65.94% (PBS), 28.47% (Van), 26.37% (ct@HMCF‐Dex alone), and only 1.69% (ct@HMCF‐Dex + NIR) (Figure S41a,b). Body weight measurements showed no significant differences among the treatment groups (Figure S41c), indicating no systemic toxicity. Blood analyses and demonstrated that levels of white blood cells, lymphocytes, monocytes, and neutrophils in mice treated with Van, ct@HMCF‐Dex, or ct@HMCF‐Dex + NIR all returned to normal range (Figure S42).

Additionally, RNA was extracted from mouse wound skin tissue and reversed into cDNA to evaluate the expression of inflammatory factors (IL‐6, TNF‐α, and IL‐1β). As shown in Figure 9e–g, the levels of IL‐6 (Figure 9e), TNF‐α (Figure 9f), and IL‐1b (Figure 9g) were significantly downregulated in the Van group, ct@HMCF‐Dex, and ct@HMCF‐Dex + NIR groups. Furthermore, wound recovery factors, including bFGF (Figure 9h), COL‐III (Figure 9i), and VEGF (Figure 9j), were all significantly upregulated in the ct@HMCF‐Dex and ct@HMCF‐Dex + NIR groups. In contrast, the Van group showed no significant difference compared to the PBS group, suggesting that while Van exerts antimicrobial and anti‐inflammatory effects, it does not actively promote wound recovery. The wound tissue recovery was further evaluated using H&E and Masson staining. In the PBS group (Figure 9d), the dermal collagen fibers showed a loose, disorganized arrangement with reduced density (black arrows), accompanied by significant inflammatory cell infiltration (red arrows). The Van group displayed localized reductions in collagen fiber content (black arrows) along with moderate inflammatory responses (red arrows). In contrast, the ct@HMCF‐Dex group maintained an intact epidermal structure, though the dermal collagen fibers remained sparsely distributed (black arrowheads), with focal areas of hemorrhage (green arrowheads) and minimal inflammatory infiltration (red arrowheads). Hair follicles (blue arrows) were visible, alongside occasional hemorrhagic regions (green arrows). Most strikingly, the ct@HMCF‐Dex + NIR group demonstrated a well‐preserved epidermal layer and densely organized dermal collagen fibers, with only mild inflammatory cell presence (red arrows) and clearly distinguishable hair follicles (blue arrows). This group exhibited the most favorable tissue regeneration outcomes among all treatments. These histological findings are consistent with the wound closure results (Figure 9b) and the expression patterns of inflammatory and healing‐related markers (Figure 9e–j), indicating that ct@HMCF‐Dex not only exerts antibacterial and anti‐inflammatory effects but also actively enhances tissue regeneration. This accelerated healing may be attributed to the pro‐angiogenic and pro‐migratory effects of copper ions, which stimulate endothelial cells, fibroblasts, and keratinocytes, thereby promoting angiogenesis, granulation tissue formation, collagen deposition, and re‐epithelialization. Collectively, these mechanisms contribute to faster and more efficient wound repair in acute injuries [58, 59]. Additionally, consistent with the in vivo biosafety observed in the pneumonia model, no significant differences were found in red blood cell counts, hemoglobin levels, or other hematological parameters between healthy mice and those treated with either ct@HMCF‐Dex alone or ct@HMCF‐Dex + NIR (Figure S42). There were no significant changes in the biochemical indicators of liver and kidney function in mice (Figure S43). Furthermore, histological examination (H&E staining) of major organs (heart, liver, spleen, lungs, and kidneys) revealed no pathological changes, further confirming the excellent in vivo safety of ct@HMCF‐Dex (Figure S44).

## 3 Conclusions

This study developed a novel “pouring fuel on the fire” strategy by constructing an auto‐amplifying iron‐copper symbiotic cycle for potent antimicrobial therapy. Hollow mesoporous copper‐iron sulfide nanoparticles (HMCF) were synthesized via a one‐pot method, loaded with citric acid (ct@HMCF), and coated with carboxylated dextran (ct@HMCF‐Dex) for stability and bacterial targeting. In acidic infection sites, ct@HMCF‐Dex releases citric acid, S^2−^, Cu^2+^, and Fe^3+^. S^2−^ produces H_2_S, increasing acidity and H_2_O_2_ levels, which accelerates nanoparticle breakdown and sustained ion release. Fe^2+^ and Cu^+^ undergo Fenton reactions with H_2_O_2_, generating toxic •OH while cycling between Fe^3+^/Fe^2+^ and Cu^2+^/Cu^+^ via GSH depletion and metal ion exchange. Citrate enhances this cycle, intensifying oxidative stress. The resulting ROS, Fe^2+^, and Cu^+^ accumulation disrupts bacterial metabolism, causing LPO and cuproptosis/ferroptosis‐like death. Additionally, quorum sensing is inhibited. Both in vitro and in vivo tests demonstrate potent antibacterial and anti‐biofilm efficacy, showcasing a “fuel‐on‐fire” strategy for synergistic metal‐ion therapy.

## 4 Experimental Section

### 4.1 Materials

Poly(vinylpyrrolidone) (PVP30, Mw = 40 kDa), copper (II) chloride dihydrate (CuCl_2_·2H_2_O), hydrazine (N_2_H_4_, 1.0 M in ethanol), citric acid (ct), sodium hydroxide (NaOH), and hydrogen peroxide (H_2_O_2_, 30%) were purchased from Sigma–Aldrich (USA). Sodium sulfide nonahydrate (Na_2_S·9H_2_O, 99.9%), glutathione (GSH, 98.0%), 5,5′‐dithiobis‐2‐(nitrobenzoicacid) (DTNB), and 3,3′,5,5′‐Tetramethylbenzidine (TMB) were bought from Shanghai Macklin Biochemical Co., Ltd. H_2_S probe (WSP‐1) was purchased from Beijing Solarbio Biological Technology Co., Ltd.

### 4.2 Synthesis of HMCF Nanoparticles

HMCF nanoparticles were synthesized through a one‐pot approach. In brief, 1200 mg of polyvinylpyrrolidone (PVP) was dissolved in 125 mL of an aqueous solution containing copper(II) chloride (2.0 mM) and iron (III) chloride (0.5 mM) under continuous magnetic stirring. Subsequently, 125 mL of sodium hydroxide (0.2 mM) was gradually introduced, followed by the addition of 1.0 mL of hydrazine hydrate (1.0 M). After 20 min of stirring, the sodium sulfide nonahydrate (320 mg/mL) aqueous stock solution was incorporated. The reaction proceeded in an oil bath at 60°C for 2.5 h under constant stirring. The resulting nanoparticles were harvested via centrifugation (20,000 × g, 15 min) and purified by multiple washes with deionized water and anhydrous ethanol to eliminate residual impurities.

### 4.3 Synthesis of Ct@HMCF Nanoparticles

1.0 mL citric acid solution (10 mg/mL) was adjusted to pH 7.4 and combined with an equal volume of HMCF dispersion. After vortexing for 30 s, the mixture was incubated overnight at room temperature to promote conjugation. The resulting ct@HMCF composite was isolated by centrifugation at 20 000 × g for 10 min and washed three times with deionized water to remove any unbound citric acid. After completion of the loading process, the suspension was centrifuged at 12 000 rpm for 10 min at 4°C, and the supernatant was collected for analysis. The loading amount of citric acid was quantified using a commercial citric acid assay kit (Nanjing Jiancheng Bioengineering Institute, China, A128‐1‐1) according to the manufacturer's instructions. Briefly, An aliquot (100 µL) of the supernatant was mixed with the corresponding assay reagents provided in the kit and incubated at room temperature for 30 min. The absorbance was measured at 545 nm using a UV–vis spectrophotometer. The amount of citric acid loaded into the nanoplatform was determined by subtracting the residual citric acid in the supernatant from the total amount initially added. All measurements were performed in triplicate.

$$\mathrm{Citric}\;\mathrm{acid}\;\mathrm{content}\left(\mu \mathrm{mol}/L\right)=2.5\times \frac{A_{\mathrm{Measurement}}-A_{\mathrm{Blank}}}{A_{\mathrm{Standard}}-A_{\mathrm{Blank}}}$$

### 4.4 Synthesis of Ct@HMCF‐Dex Nanoparticles

10 mg of ct@HMCF was aminated by stirring with mercaptoethylamine for 4 h, yielding ct@HMCF‐NH_2_, which was then washed and dispersed (Solution A). Carboxylated dextran (Dex‐COOH) was activated in PBS using ethyldimethylaminopropyl carbodiimide (EDC)/N‐hydroxy succinimide(NHS) (1:1:1 molar ratio) for 1 h (Solution B). Solution A was then added dropwise to Solution B and reacted for 24 h. The final product, ct@HMCF‐Dex, was purified via centrifugation.

### 4.5 Characterization

Nanoparticle morphology and corresponding energy dispersive X‐ray spectroscopy (EDS) spectra were analyzed using a transmission electron microscope (TEM) (Talos G2 200X). Particle size and zeta potential were determined via dynamic light scattering (DLS) using a BeNano 90 Zeta analyzer. UV–vis absorption spectra were recorded with a UV‐2700i spectrophotometer. Fourier transform infrared (FT‐IR) spectra were obtained using a Nicolet IS50 spectrometer, while X‐ray photoelectron spectroscopy (XPS) measurements were conducted with a Thermo Scientific ESCALAB 250XI. X‐ray diffraction (XRD) phase analysis was performed using a Japan Science SmartLab 9 kW diffractometer.

### 4.6 In Vitro Antimicrobial

The antibacterial activity of ct@HMCF‐Dex against methicillin‐resistant *Staphylococcus aureus* (MRSA) and *Pseudomonas aeruginosa* (*P. aeruginosa*) was assessed through plate colony counting and live/dead bacterial staining under different conditions. Additionally, bacterial morphology post‐treatment was characterized using scanning electron microscopy (SEM). Membrane permeability was evaluated using 2‐Nitrophenyl β‐D‐galactopyranoside (ONPG), while protein leakage levels were quantified with a BCA kit. Changes in reactive oxygen species (ROS), hydrogen sulfide (H_2_S), and membrane potential were analyzed using fluorescent probes DCFH‐DA, WSP‐1, and DiOC_2_(3), respectively.

Lipid peroxidation levels were assessed using BODIPY 581/591 C11 (Beyotime), whereas malondialdehyde (MDA) content, ATP levels, glutathione (GSH) concentration, and oxidized glutathione (GSSG) levels were quantified using commercial assay kits (Nanjing Jiancheng). Additionally, intracellular ferrous ion and copper ion concentrations were determined using a ferrous ion content kit (Solarbio) and a copper ion fluorescence probe (Solarbio), respectively.

### 4.7 In Vitro Antibiofilm Capacity

The bacterial suspension (1 × 10^7^ CFU/mL) was introduced into 24‐well plates and incubated at 37°C for 24 h to allow mature biofilms to develop. In the biofilm disruption assay, these biofilms were treated with PBS, PBS + H_2_O_2_, ct@HMCF‐Dex, or ct@HMCF‐Dex + H_2_O_2_. Following treatment, residual biofilms were gently rinsed three times with PBS. The extent of biofilm degradation was assessed through crystal violet staining and viable bacterial counting. Additionally, biofilms were formed on glass slides and subjected to the same treatments. After staining with SYBR Green I for 30 min, their 3D structures were visualized using laser confocal microscopy.

For the biofilm inhibition assay, bacterial suspensions (1 × 10^7^ CFU/mL) were pretreated with PBS, PBS + H_2_O_2_, ct@HMCF‐Dex, or ct@HMCF‐Dex + H_2_O_2_. The resulting biofilm formation was then evaluated using crystal violet staining, viable bacterial counting, and 3D structural imaging, following the same procedures as described above.

### 4.8 Therapeutic Effects of Ct@HMCF‐Dex In Vivo

To evaluate the in vivo therapeutic efficacy of ct@HMCF‐Dex, two animal models were established: a bacterial‐infected pneumonia model and a bacterial‐infected wound model. In the pneumonia model, mice were intratracheally inoculated with 50 µL of MRSA (10^7^ CFU/mL) resuspended in PBS using the tracheal drip method. To ensure uniform bacterial distribution across both lungs, the mice were gently shaken after administration. After 2 h, they received different nebulization treatments. At 24 and 48 h post‐treatment, the mice were euthanized, and their lungs were excised, photographed, and homogenized using a high‐throughput tissue milling instrument. Bacterial counts in the lung homogenates were measured to assess the antimicrobial efficacy of each treatment. Additionally, total RNA was extracted from lung tissue, reverse transcribed into cDNA, and analyzed by reverse transcription‐polymerase chain reaction (RT‐PCR) to quantify IL‐1β, IL‐6, and TNF‐α levels, providing insights into the anti‐inflammatory response. Lung tissue sections were further examined via H&E staining to assess inflammatory cell infiltration at 24 and 48 h.

For the wound infection model, a 1.0 cm diameter circular wound was excised on each mouse and inoculated with MRSA (10^7^ CFU/mL). After 24 h, mice received designated treatments. Wound closure was tracked by photographic documentation on days 1, 3, 5, 7, and 9, and bacterial counts in wound tissues were quantified to assess antimicrobial activity. Concurrently, RNA extracted from skin tissue was reverse‐transcribed into cDNA, and RT‐qPCR was performed to analyze the expression of IL‐1β, IL‐6, TNF‐α, VEGF, and other healing‐related markers, evaluating both anti‐inflammatory effects and pro‐healing activity. On day 9, wound tissues were harvested for histopathological analysis using H&E and Masson staining to assess tissue regeneration and collagen deposition.

All animal experiments in this study followed the principles established by the Animal Ethics Committee of Tianjin University of Science & Technology (20240910).

### 4.9 Statistical Analysis

Statistical significance between two groups was assessed using one‐way analysis of variance (ANOVA). Data are presented as mean ± standard deviation, with *p* < 0.05 considered statistically significant (^*^ *p* < 0.05, ^**^ *p* < 0.01, ^***^ *p* < 0.001, ^****^ *p* < 0.0001).

## Author Contributions

Z.X. contributed to conception, data curation, formal analysis, investigation, methodology, and drafted and revised the manuscript. S.Q. contributed to data acquisition, formal analysis, and investigation. J.C. contributed to data curation, formal analysis, and investigation. J.L. contributed to data analysis and methodology. Z.S. contributed to data analysis and methodology. T.D. contributed to conceptualization, funding acquisition, supervision, validation, and revised the manuscript. X.D. contributed to funding acquisition, methodology, supervision, and revised the manuscript.

## Conflicts of Interest

The authors declare no conflicts of interest.

## Supporting information




**Supporting File**: advs75101‐sup‐0001‐SuppMat.docx.

## Data Availability

The data that support the findings of this study are available from the corresponding author upon reasonable request.
