# Supplementary material for: Self‐Amplifying Redox Cycle Triggers Ferroptosis/Cuproptosis Synergy for Enhanced Bacterial Eradication
Source: Adv Sci (Weinh). 2026 Apr 2;13(34):e75101. doi: 10.1002/advs.75101 (PMC13285168; doi:10.1002/advs.75101)
Supplement: Supplementary file 1 — Supporting File: advs75101‐sup‐0001‐SuppMat.docx. [file ADVS-13-e75101-s001.docx]

**Self-Amplifying Redox Cycle Triggers Ferroptosis/Cuproptosis Synergy for Enhanced Bacterial Eradication**

Zehui Xiao^[[1]](#footnote-1)^, Shaolong Qiu^1^, Jiangli Cao^1^, Jifeng Liu^1^, Zhiyong Song^2^, Ting Du^1,^* and Xinjun Du^1,^*

**Results**


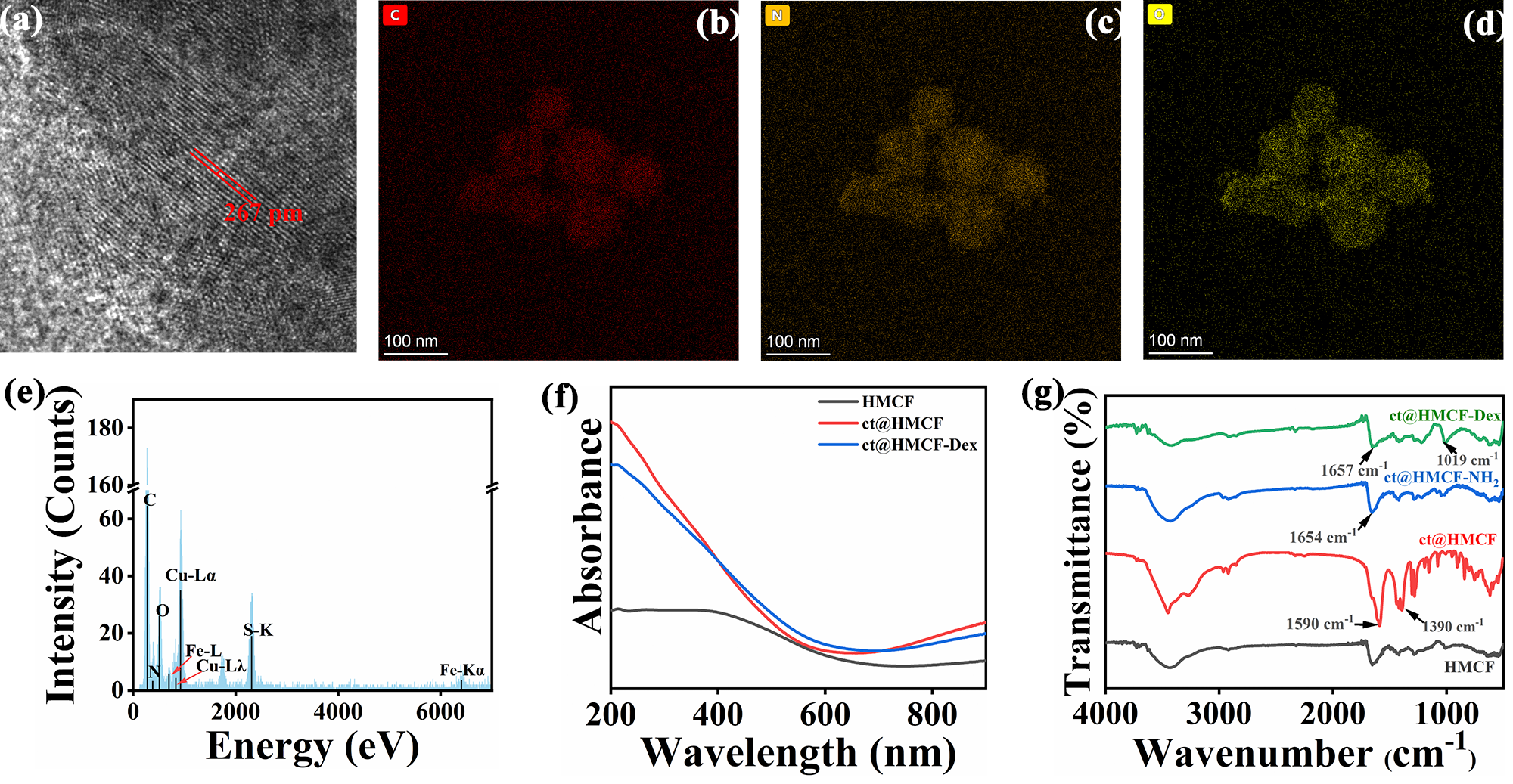


**Figure S1.** (a) The lattice structure of HMCF. C (b), N (c) and O (d) elements mapping images of ct@HMCF-Dex. (e) EDS analysis of ct@HMCF-Dex. (f) UV–vis spectra of HMCF, ct@HMCF, and ct@HMCF-Dex. (g) FTIR of HMCF, ct@HMCF, ct@HMCF-NH_2_ and ct@HMCF-Dex.


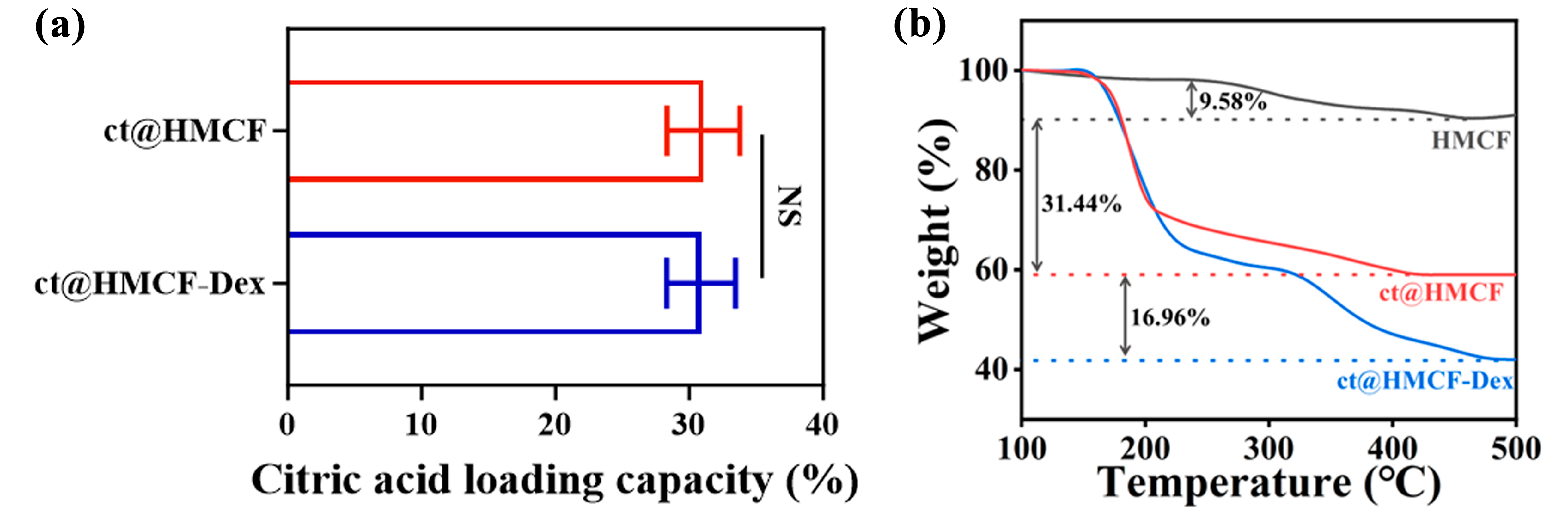


**Figure S2**. (a) The citric acid loading amounts before and after dextran modification (*n*=3). (b) TGA spectrum of HMCF, ct@HMCF and ct@HMCF-Dex. Error bars represent means ± SD. Differences between groups were tested using one-way ANOVA followed by Tukey's multiple comparisons test.


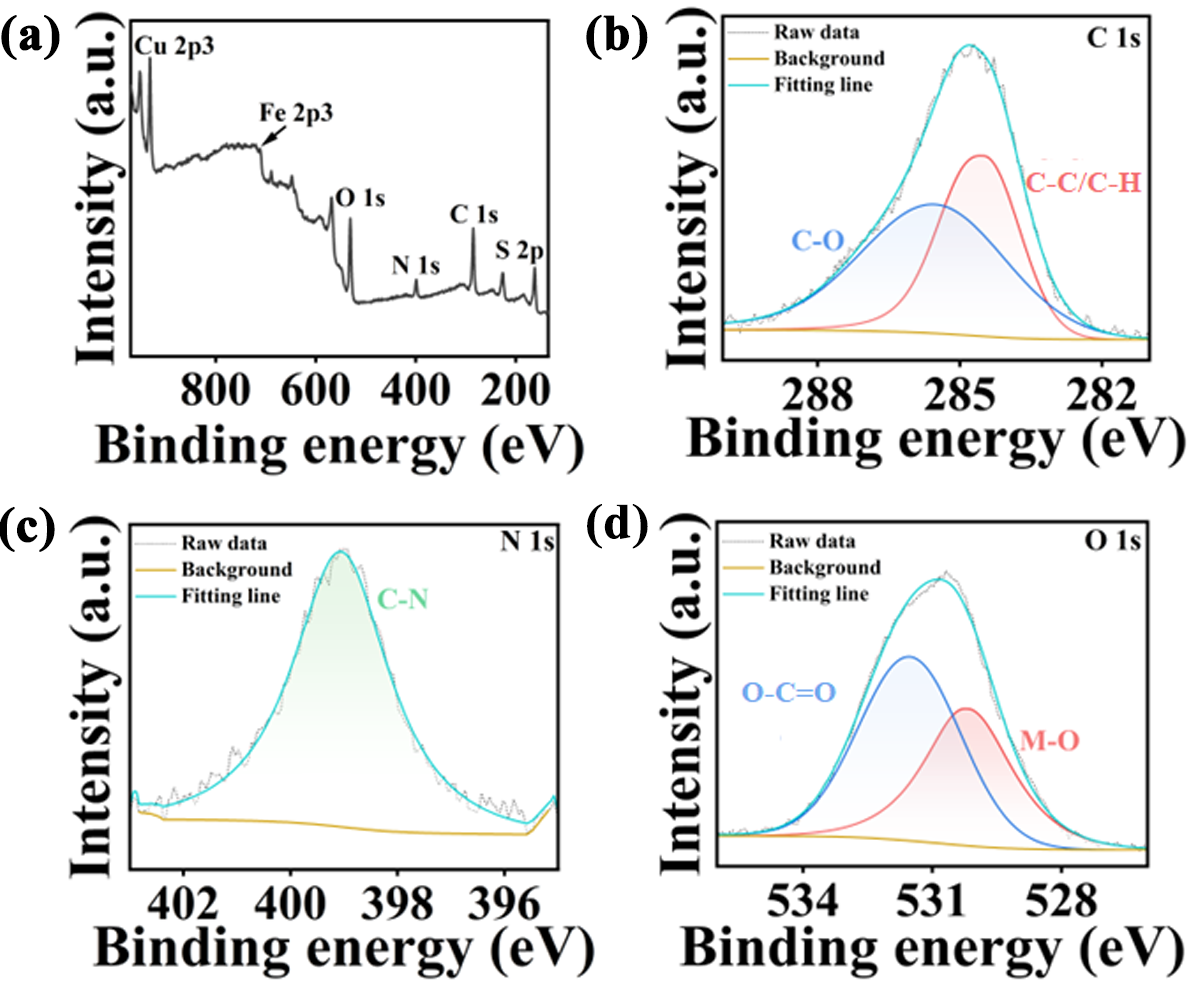


**Figure S3.** (a) XPS analysis of the ct@HMCF-Dex. High-resolution XPS spectra of C 1s (b) N 1s (c) and O 1s (d) orbitals.
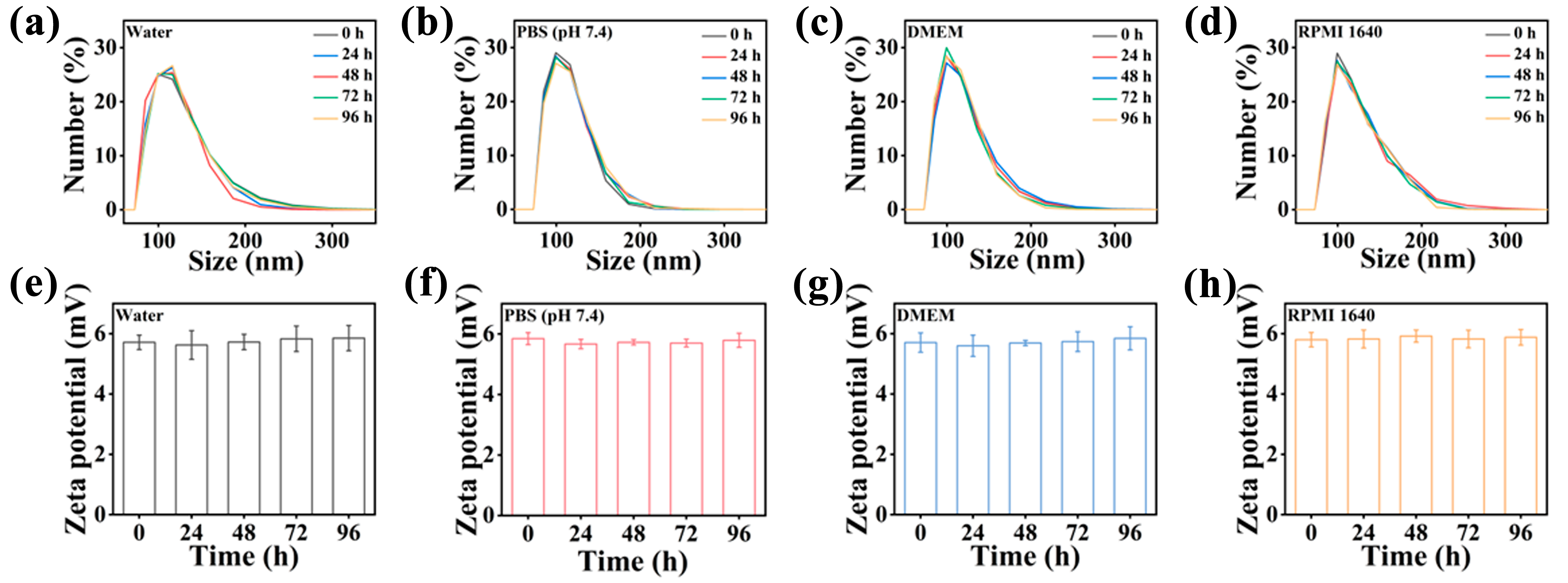


**Figure S4.** The particle size distribution of ct@HMCF-Dex in water (a), PBS (pH 7.4) (b), DMEM (c), and RPMI 1640 (d) after different incubation time. Zeta potential of ct@HMCF-Dex in water (e), PBS (pH 7.4) (f), DMEM (g), and RPMI 1640 (h) after different incubation time (*n*=3). Error bars represent means ± SD.


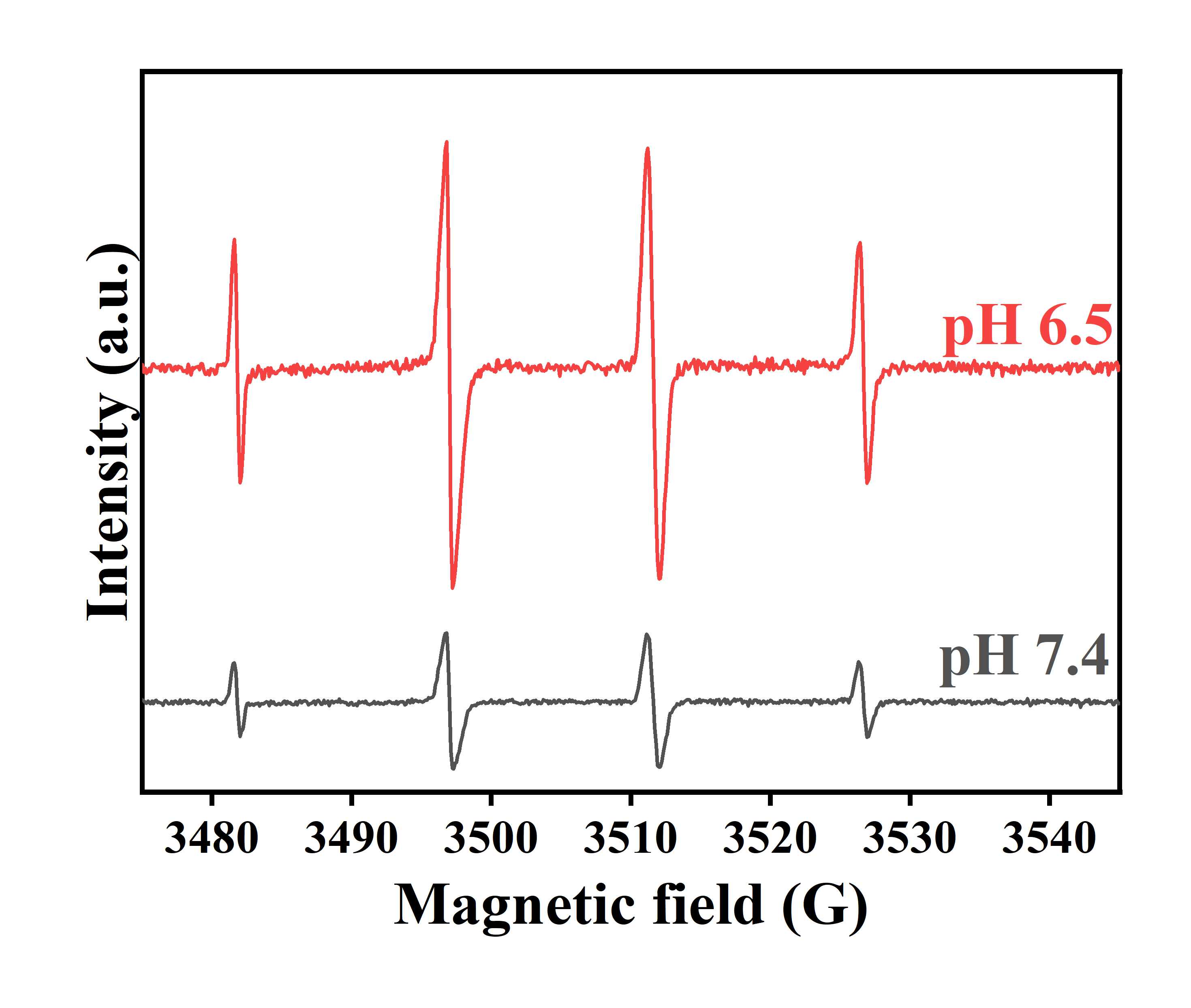


**Figure S5.** ESR measurements of DMPO-•OH for ct@HMCF-Dex under buffer conditions of pH 6.5 or pH 7.4.


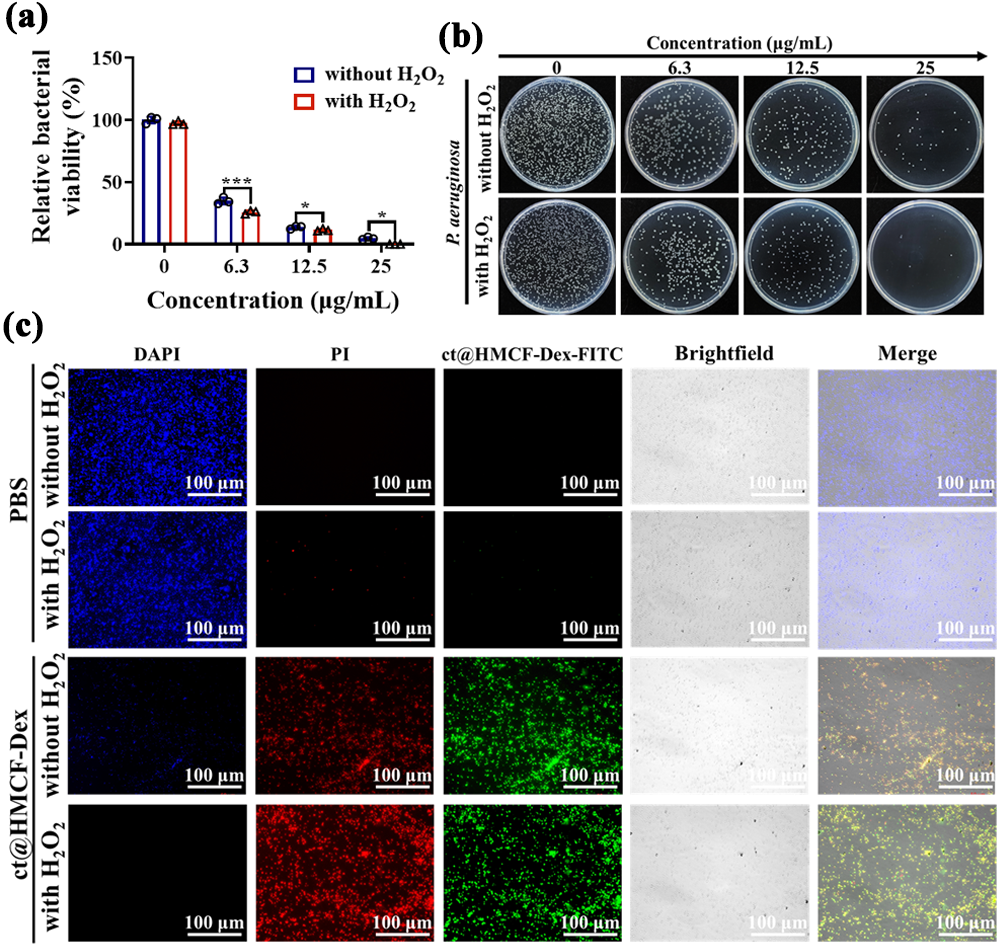


**Figure S6.** (a) Survival rate of *P. aeruginosa* after treatment of different concentration ct@HMCF-Dex (pH = 6.5) (*n*=3). (b) Agar plate photos of concentration-dependent killing of *P. aeruginosa* (pH = 6.5). (c) Fluorescence photos of *P. aeruginosa* stained with DAPI, PI and FITC after different treatments (pH = 6.5). Error bars represent means ± SD. Differences between groups were tested using one-way ANOVA followed by Tukey's multiple comparisons test. **p* < 0.05, ^***^*p* < 0.001.


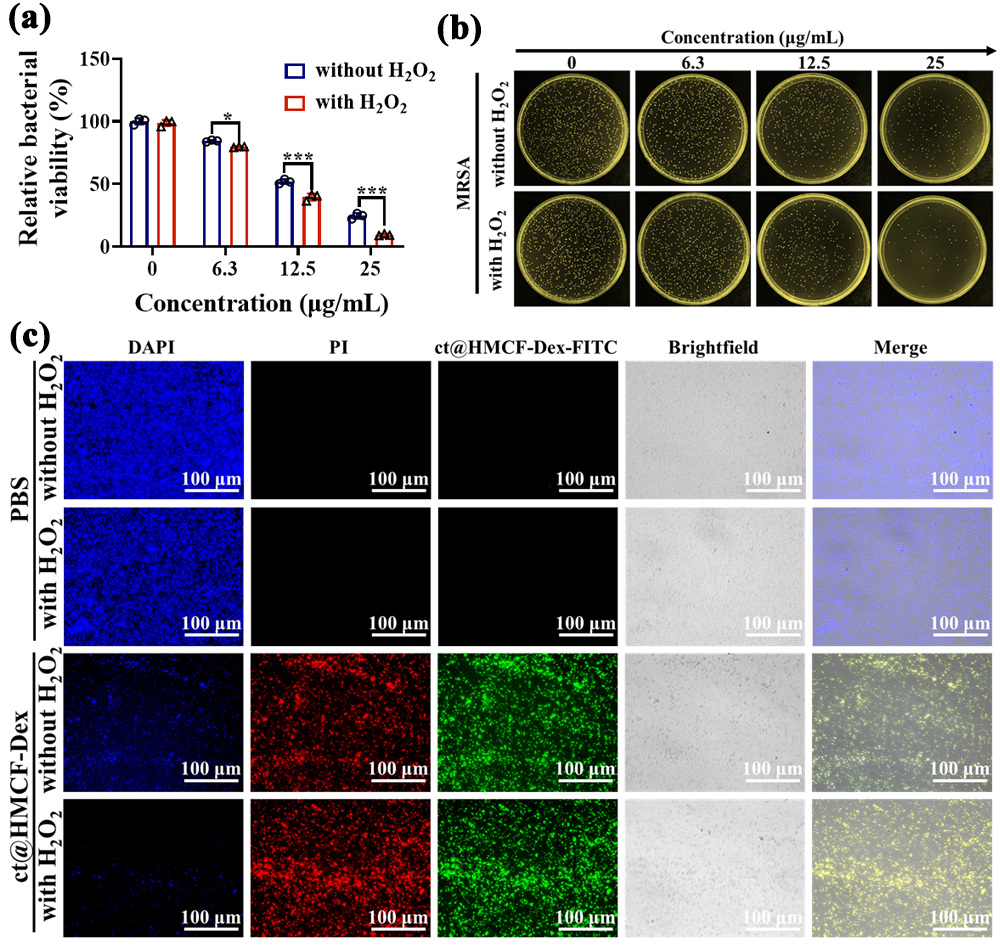


**Figure S7.** (a) Survival rate of MRSA after treatment of different concentration ct@HMCF-Dex (pH = 7.4) (*n*=3). (b) Agar plate photos of concentration-dependent killing of MRSA (pH = 7.4). (c) Fluorescence photos of MRSA stained with DAPI, PI and FITC after different treatments (pH = 7.4). Error bars represent means ± SD. Differences between groups were tested using one-way ANOVA followed by Tukey's multiple comparisons test. ^*^*p* < 0.05, ^***^*p* < 0.001.


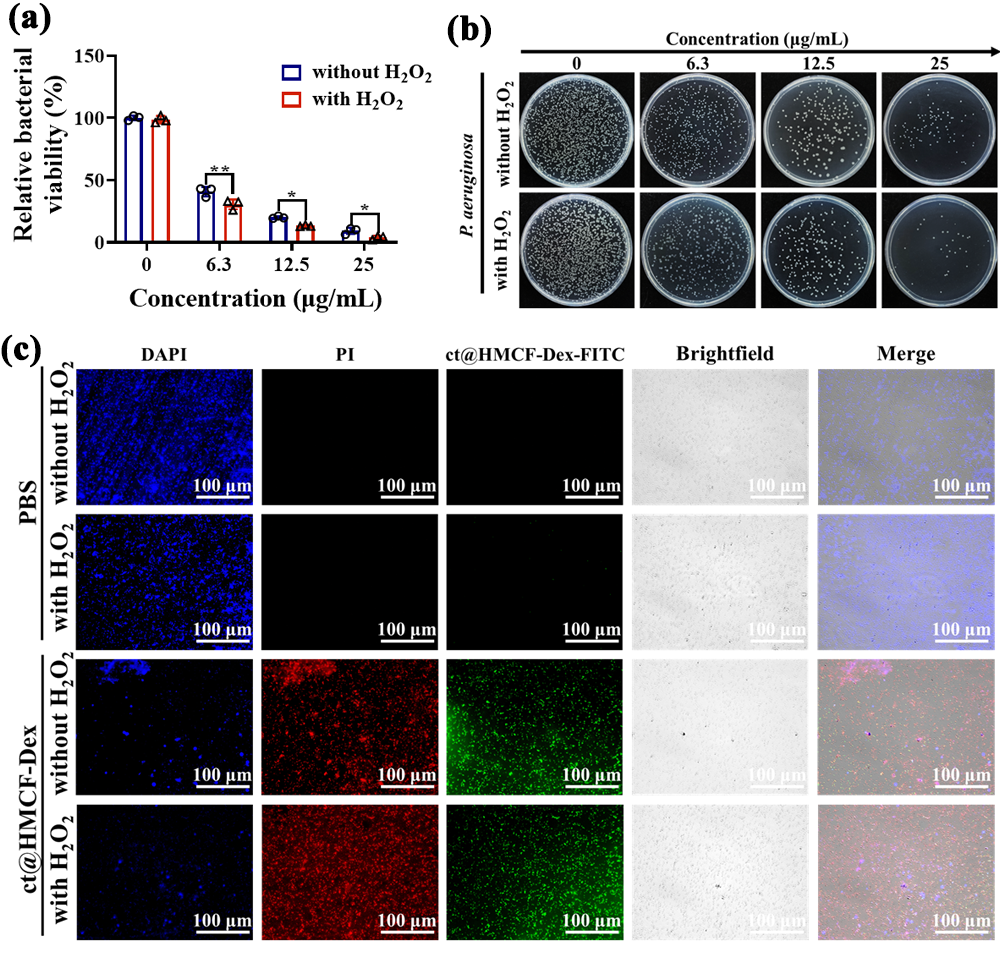


**Figure S8.** (a) Survival rate of *P. aeruginosa* after treatment of different concentration ct@HMCF-Dex (pH = 7.4) (*n*=3). (b) Agar plate photos of concentration-dependent killing of *P. aeruginosa* (pH = 7.4). (c) Fluorescence photos of *P. aeruginosa* stained with DAPI, PI and FITC after different treatments (pH = 7.4). Error bars represent means ± SD. Differences between groups were tested using one-way ANOVA followed by Tukey's multiple comparisons test. ^*^*p* < 0.05, ^**^*p* < 0.01.


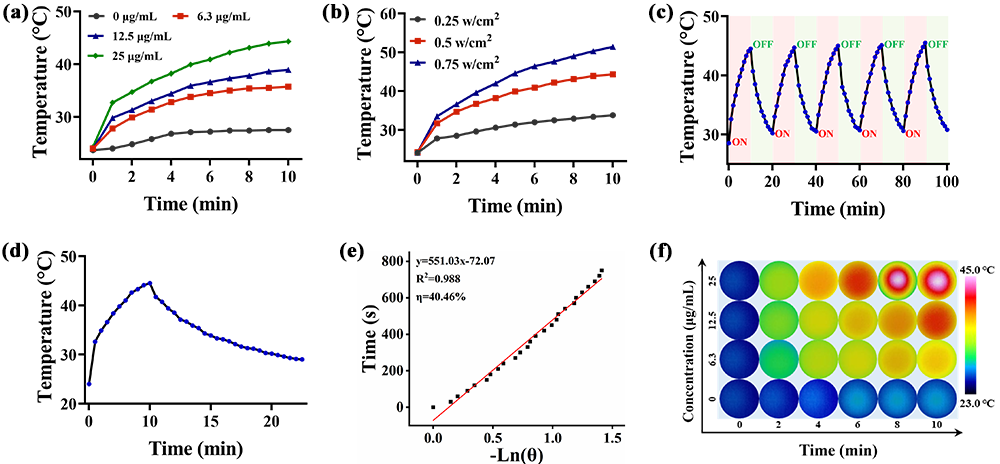


**Figure S9.** (a) Temperature changes of ct@HMCF-Dex at different concentrations under NIR irradiation (0.5 W/cm^2^). (b) Temperature curves of ct@HMCF-Dex under NIR irradiation with different powers. (c) Photothermal stability of ct@HMCF-Dex (5 laser on-off cycles). (d) Temperature variation with irradiation time and cooling time during a single on-off cycle. (e) Linear relationship between cooling period time and the negative natural logarithm of temperature. (f) Thermograms of the temperature variation of different concentrations of ct@HMCF-Dex under NIR irradiation.


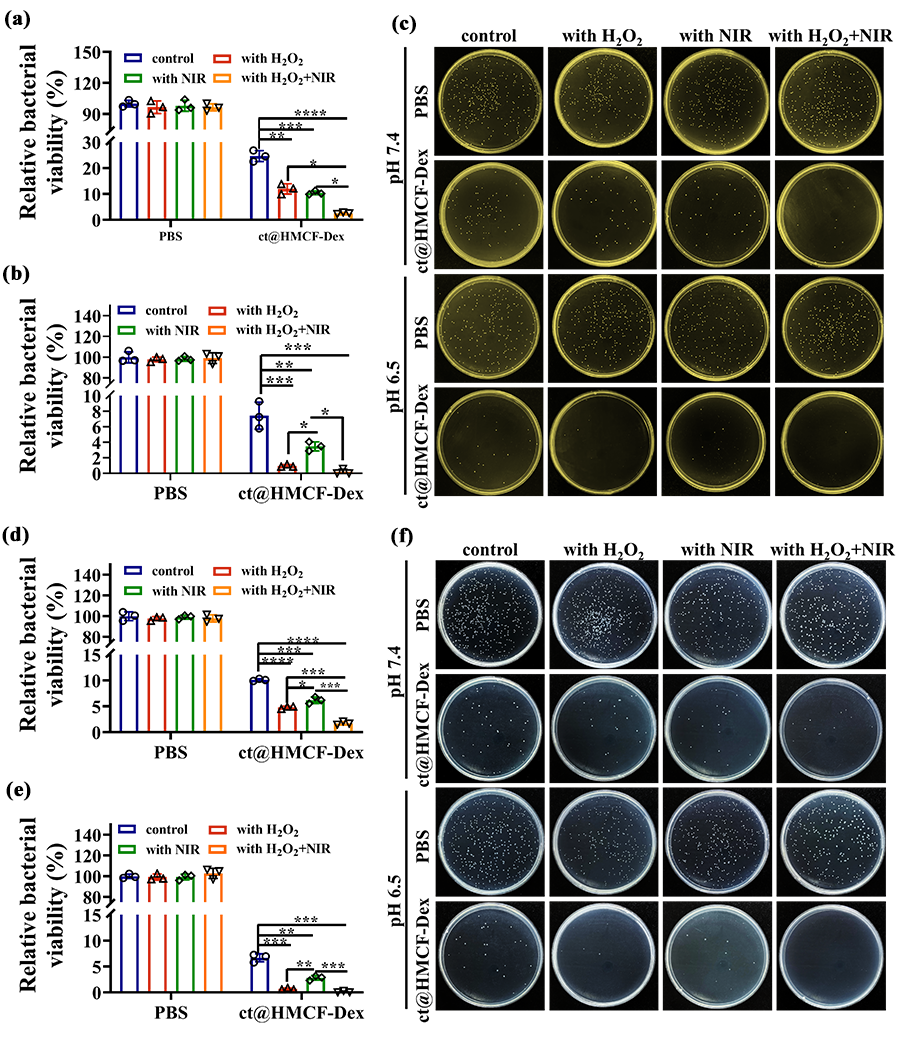


**Figure S10.** Survival rate of MRSA after different treatment (ct@HMCF-Dex, with/without NIR) under pH 7.4 (a) and pH 6.5 (b) (*n*=3). (c) Agar plate photos after different treatments of MRSA. Survival rate of *P. aeruginosa* after different treatment (ct@HMCF-Dex, with/without NIR) under pH 7.4 (d) and pH 6.5 (e) (*n*=3). (f) Agar plate photos after different treatments of *P. aeruginosa*. Error bars represent means ± SD. Differences between groups were tested using one-way ANOVA followed by Tukey's multiple comparisons test. ^*^*p* < 0.05, ^**^*p* < 0.01, ^***^*p* < 0.001, ^****^*p* < 0.0001.


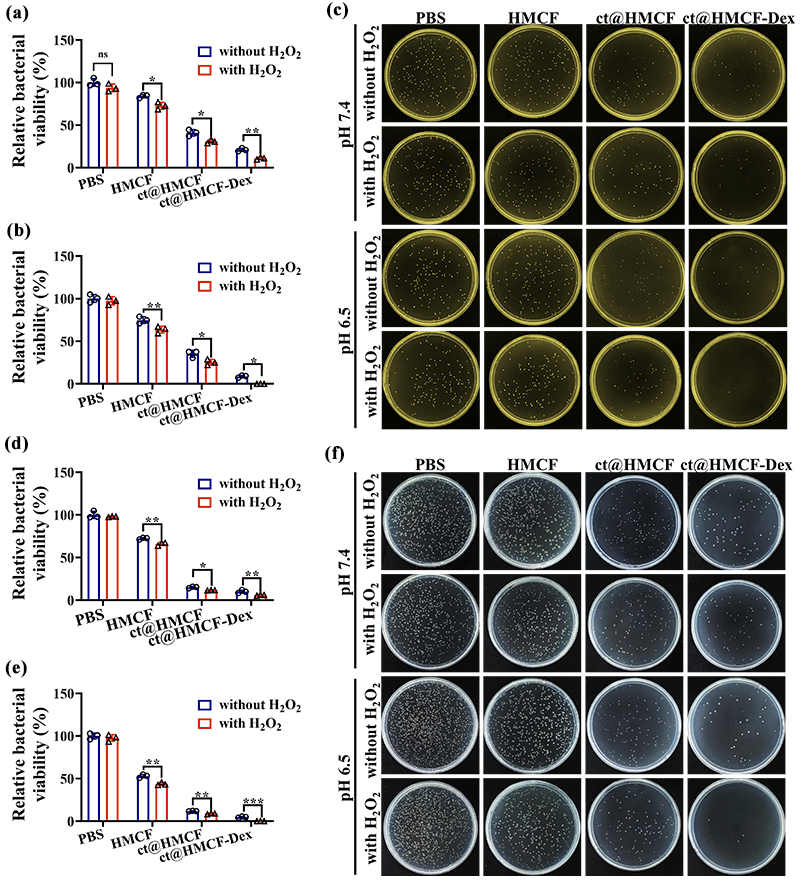


**Figure S11.** Survival rate of MRSA after different treatment (PBS, HMCF, ct@HMCF, ct@HMCF-Dex) under pH 7.4 (a) and pH 6.5 (b) (*n*=3). (c) Agar plate photos after different treatment (PBS, HMCF, ct@HMCF, ct@HMCF-Dex) of MRSA. Survival rate of *P. aeruginosa* after different treatments (PBS, HMCF, ct@HMCF, ct@HMCF-Dex) under pH 7.4 (d) and pH 6.5 (e) (*n*=3). (f) Agar plate photos after different treatments (PBS, HMCF, ct@HMCF, ct@HMCF-Dex) of *P. aeruginosa*. Error bars represent means ± SD. Differences between groups were tested using one-way ANOVA followed by Tukey's multiple comparisons test. ^*^*p* < 0.05, ^**^*p* < 0.01, ^***^*p* < 0.001.


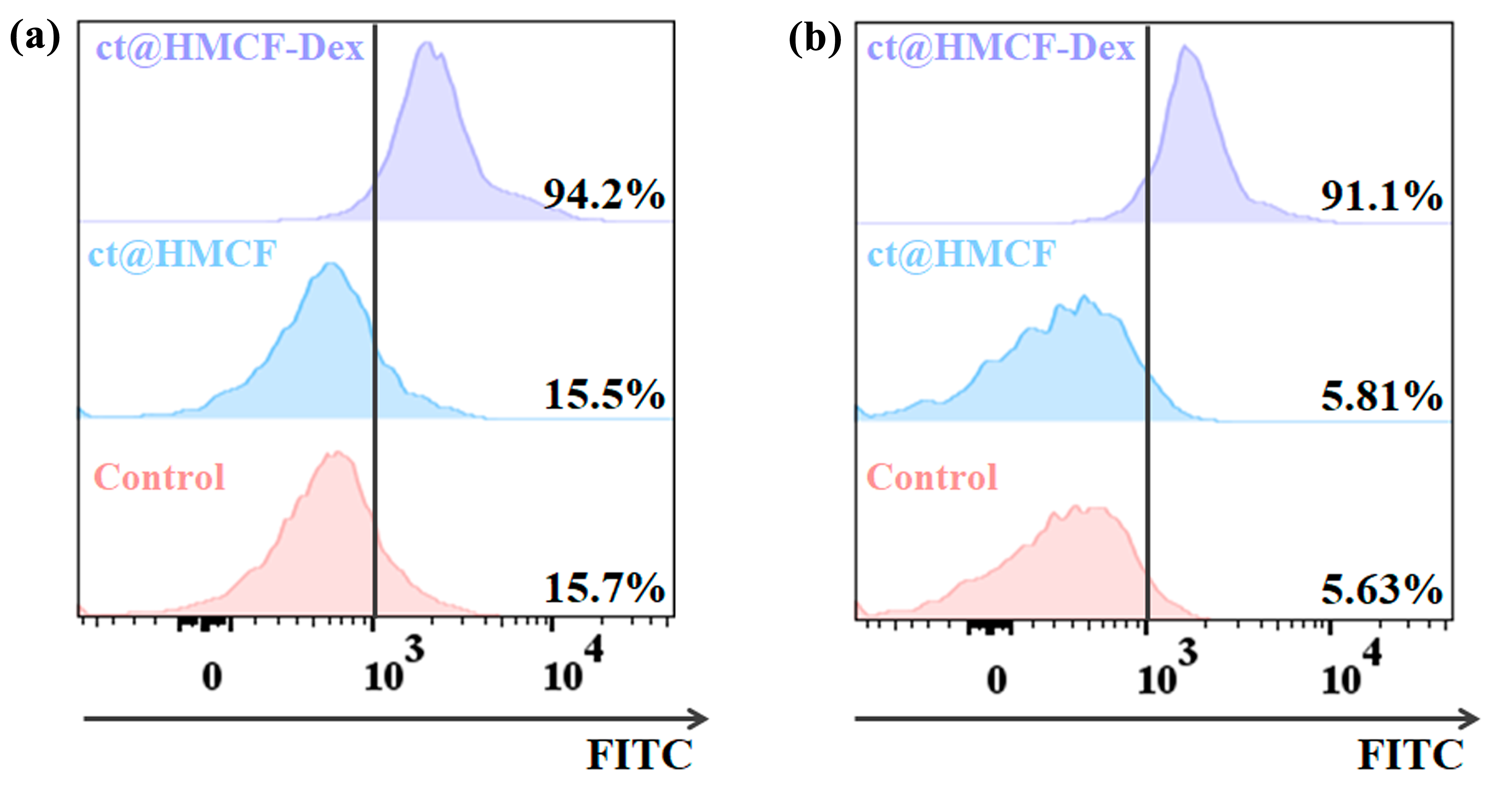


**Figure S12.** Flow cytometry analysis of fluorescence intensity after co-incubated ct@HMCF and ct@HMCF-Dex with MRSA (a) and *P. aeruginosa* treated (b).


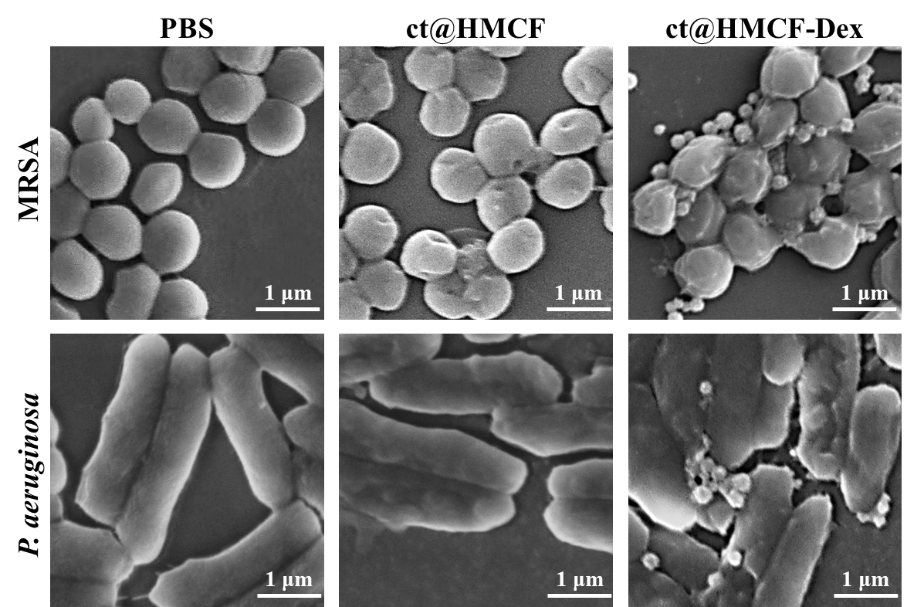


**Figure S13.** SEM images of MRSA and *P. aeruginosa* treated with ct@HMCF and ct@HMCF-Dex.


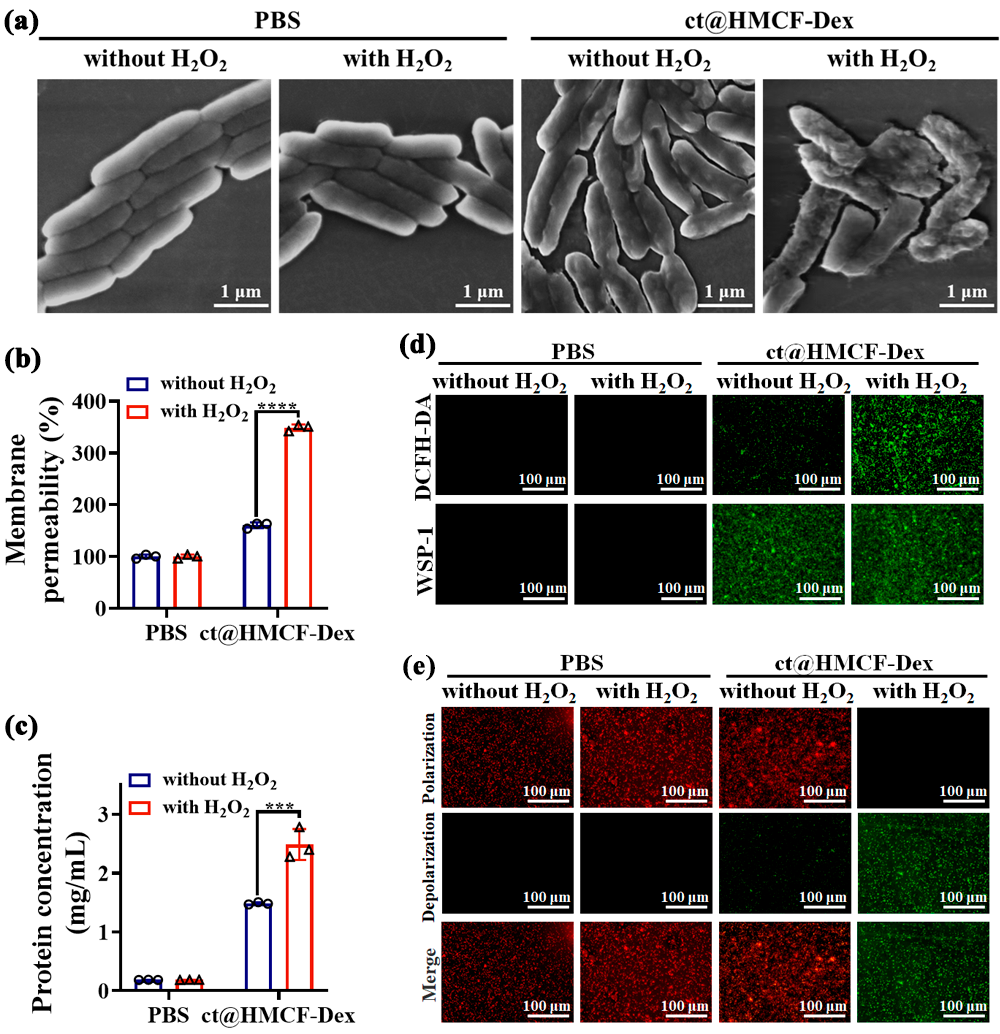


**Figure S14.** (a) Morphological observation of *P. aeruginosa* after coincubation with ct@HMCF-Dex. Membrane permeability (b) and protein leakage (c) of *P. aeruginosa* after different treatment (*n*=3). (d) Fluorescence images of ROS, H_2_S in *P. aeruginosa* after different treatments. (e) The membrane potential of *P. aeruginosa* after different treatments. Error bars represent means ± SD. Differences between groups were tested using one-way ANOVA followed by Tukey's multiple comparisons test. ^***^*p* < 0.001, ^****^*p* < 0.0001.


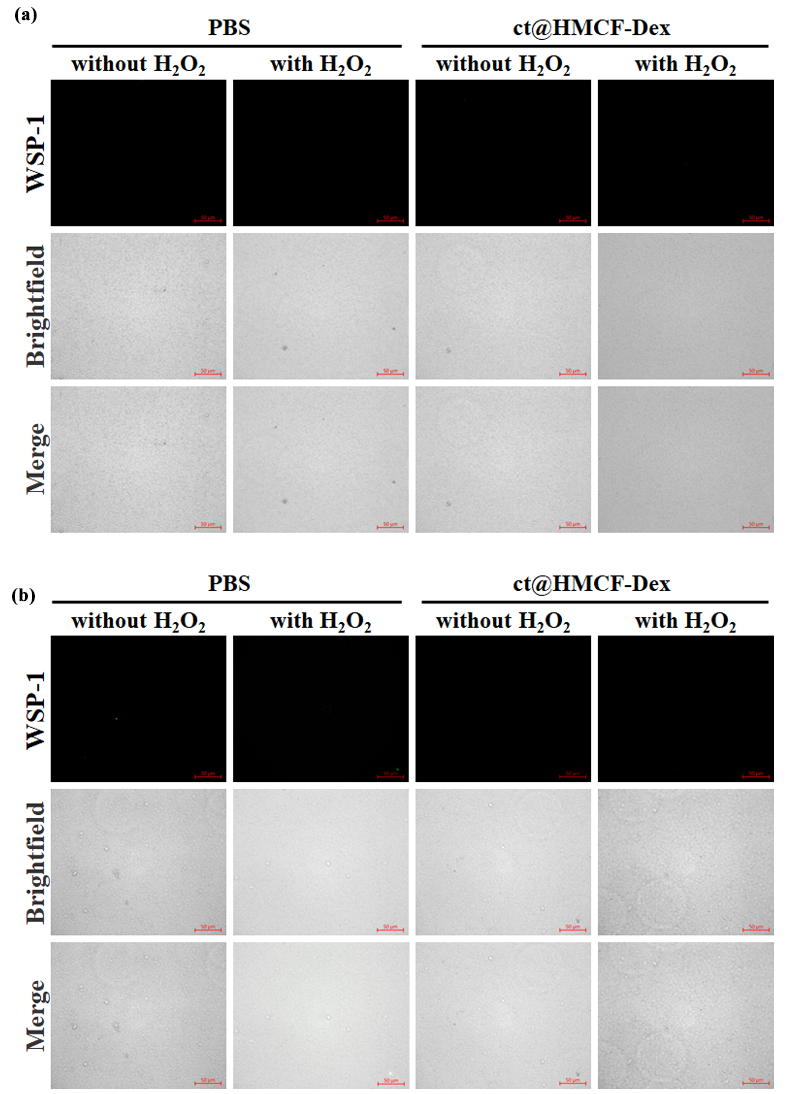


**Figure S15.** LSCM image of WSP-1-stained MRSA (a) and *P. aeruginosa* (b) at pH 7.4 showing intracellular H_2_S.


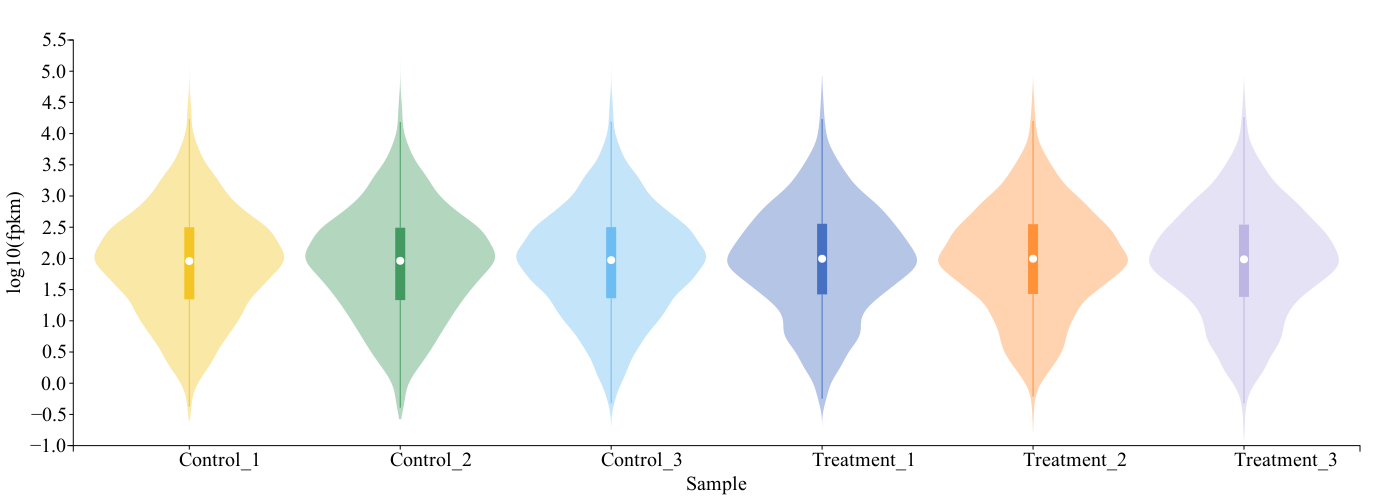


**Figure S16.** Violin plot of gene expression. The horizontal line in the middle of the box is the median, the upper and lower edges of the box are 75%, and the upper and lower limits are 90%. The outer shape is the kernel density estimate.


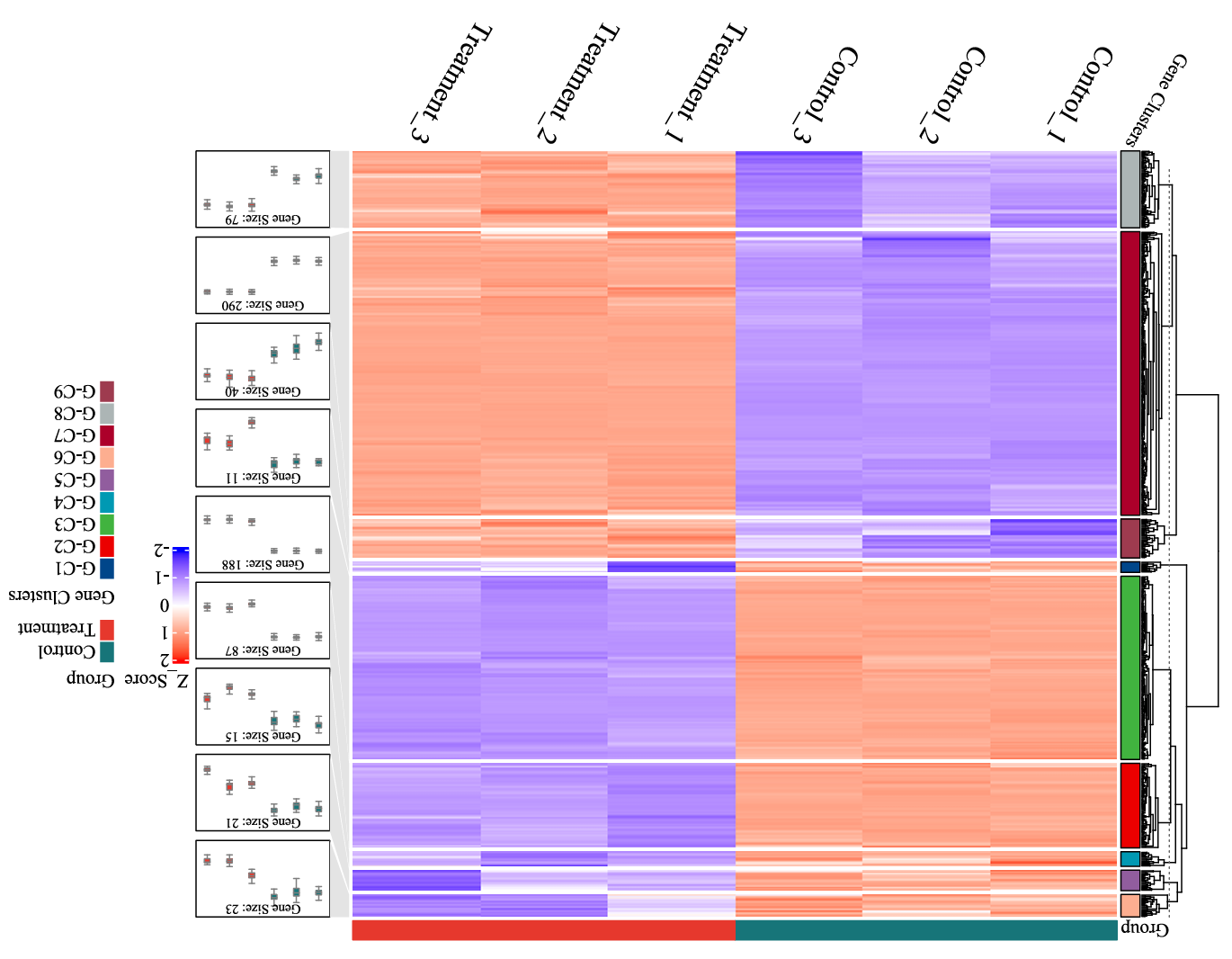


**Figure S17.** Heatmap of differential genes. Horizontal representation of genes, each column is a sample; the redder the color, the higher the expression of the gene in that sample, and the bluer the color, the lower the expression of the gene in that sample (the plot data is the expression of the gene after Zscore normalization calculation); left sample clustering tree: sample clustering situation, the samples with similar expression patterns are clustered together and divided into 1 cluster; right side color block: samples clustered together are divided using staining, different colors represent different clusters; box-and-line plot: represents the expression pattern of genes in each sample under each cluster; box represents the quartiles of the data, i.e., the median and the upper and lower quartiles of the dataset. The median represents the central tendency of the data and the upper and lower quartiles represent the range of distribution of the data.


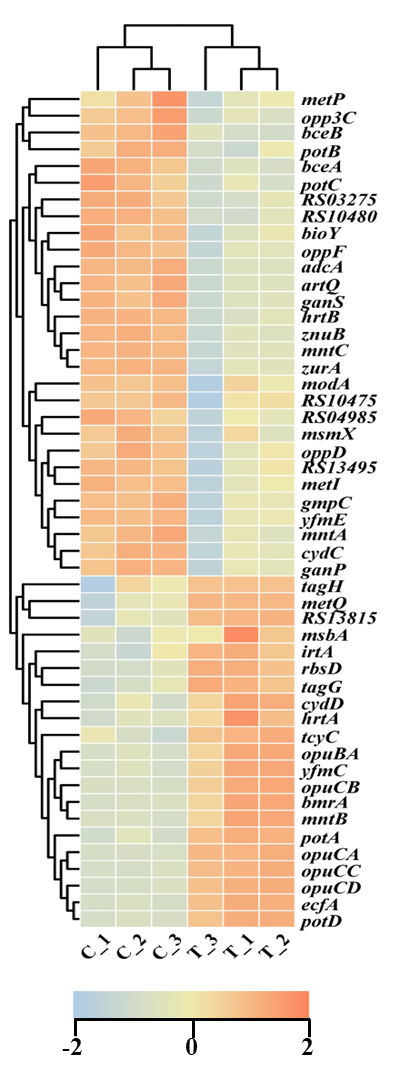


**Figure S18.** Heat map of genes associated with ABC transporters (*n*=3).


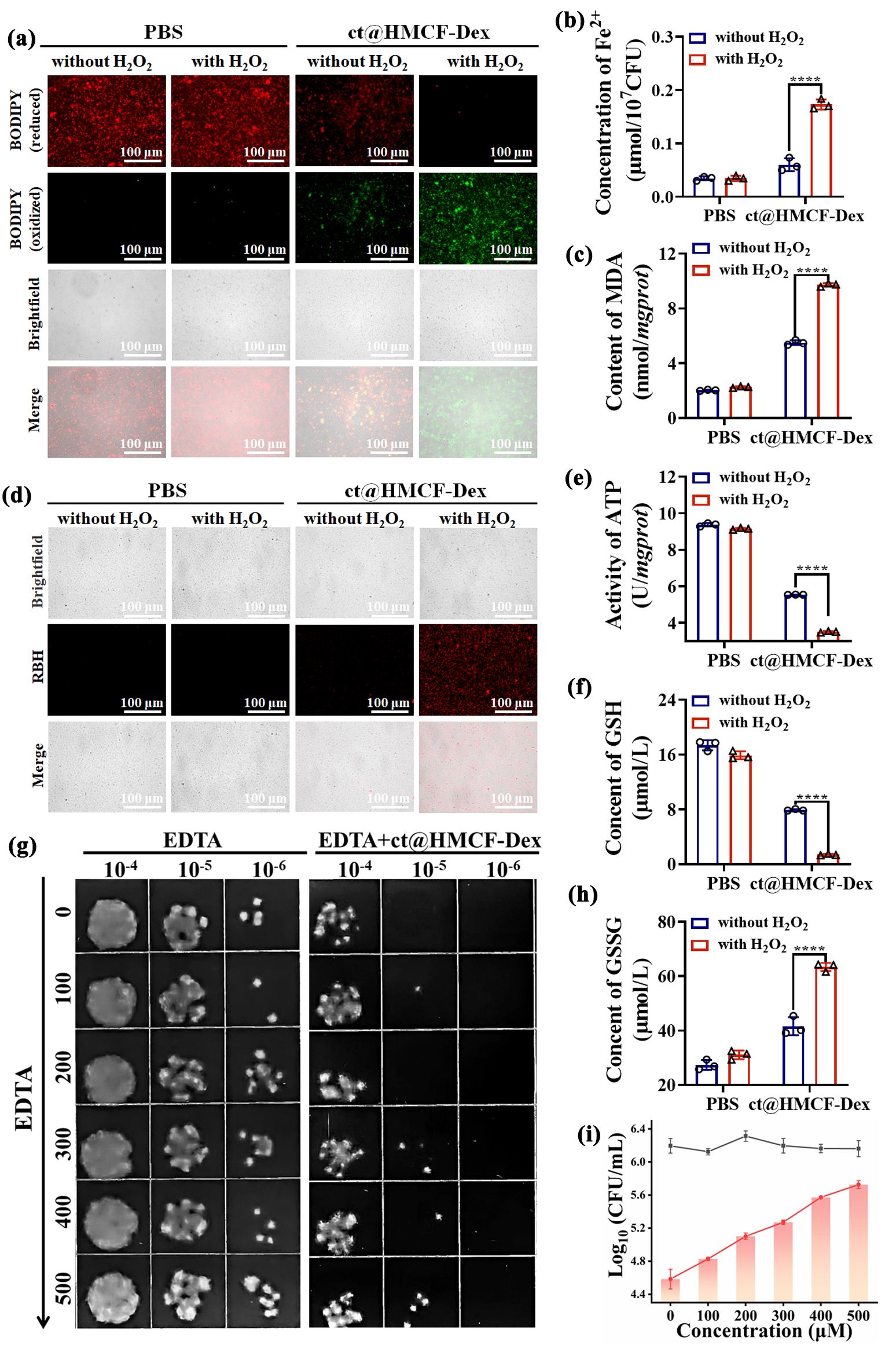


**Figure S19.** (a) Fluorescence images of lipid peroxidation levels after different treatments of *P. aeruginosa*. Changes of Fe^2+^ (b), MDA (c), ATP (e), GSH (f) and GSSG (h) contents in *P. aeruginosa* after different treatments (*n*=3). (d) Fluorescence images of copper ion levels after different treatments of *P. aeruginosa*. Colonization (g) and corresponding survival (i) of *P. aeruginosa* after co-incubation of different concentrations of EDTA with ct@HMCF-Dex (*n*=3). Error bars represent means ± SD. Differences between groups were tested using one-way ANOVA followed by Tukey's multiple comparisons test. ^****^*p* < 0.0001.


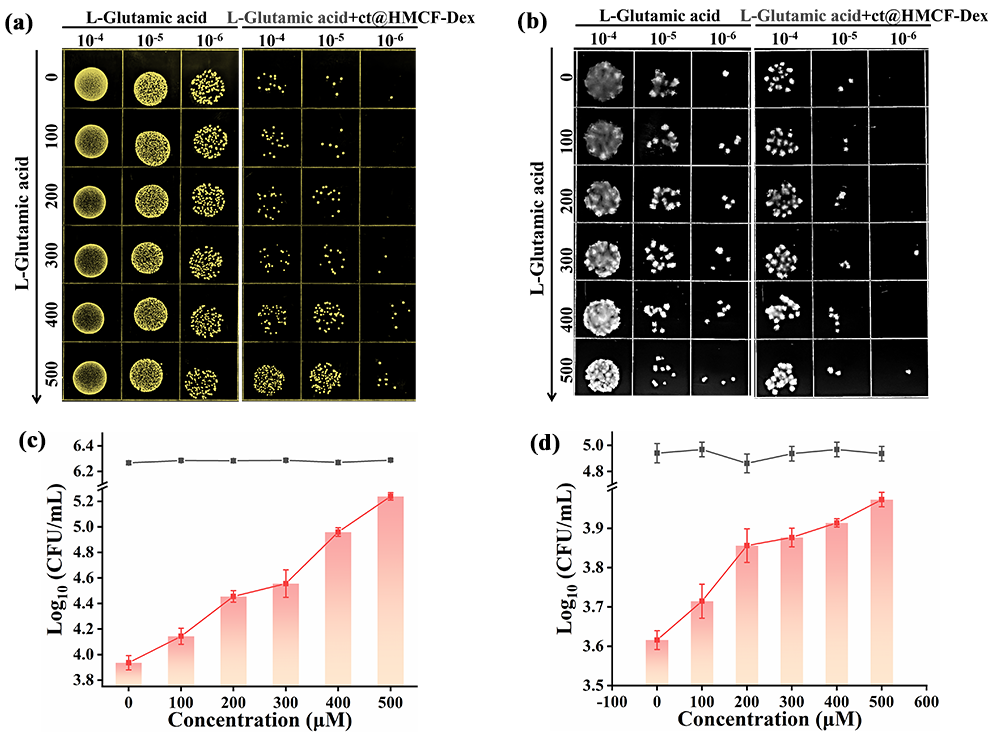


**Figure S20.** L-glutamate inhibition of ct@HMCF-Dex induced MRSA (a) and *P. aeruginosa* (b) death. Quantitative analysis of MRSA (c) and *P. aeruginosa* (d) after different treatments (*n*=3). Error bars represent means ± SD.


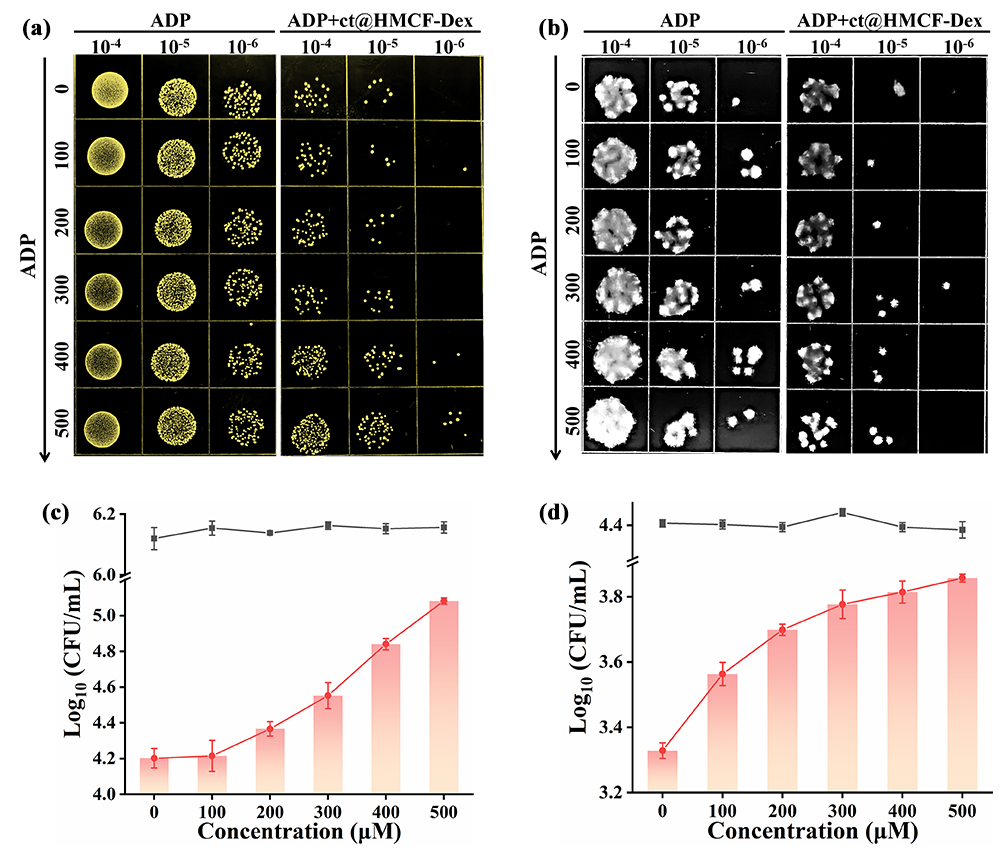


**Figure S21.** ADP inhibition of ct@HMCF-Dex induced MRSA (a) and *P. aeruginosa* (b) death. Quantitative analysis of MRSA (c) and *P. aeruginosa* (d) after different treatments (*n*=3). Error bars represent means ± SD.


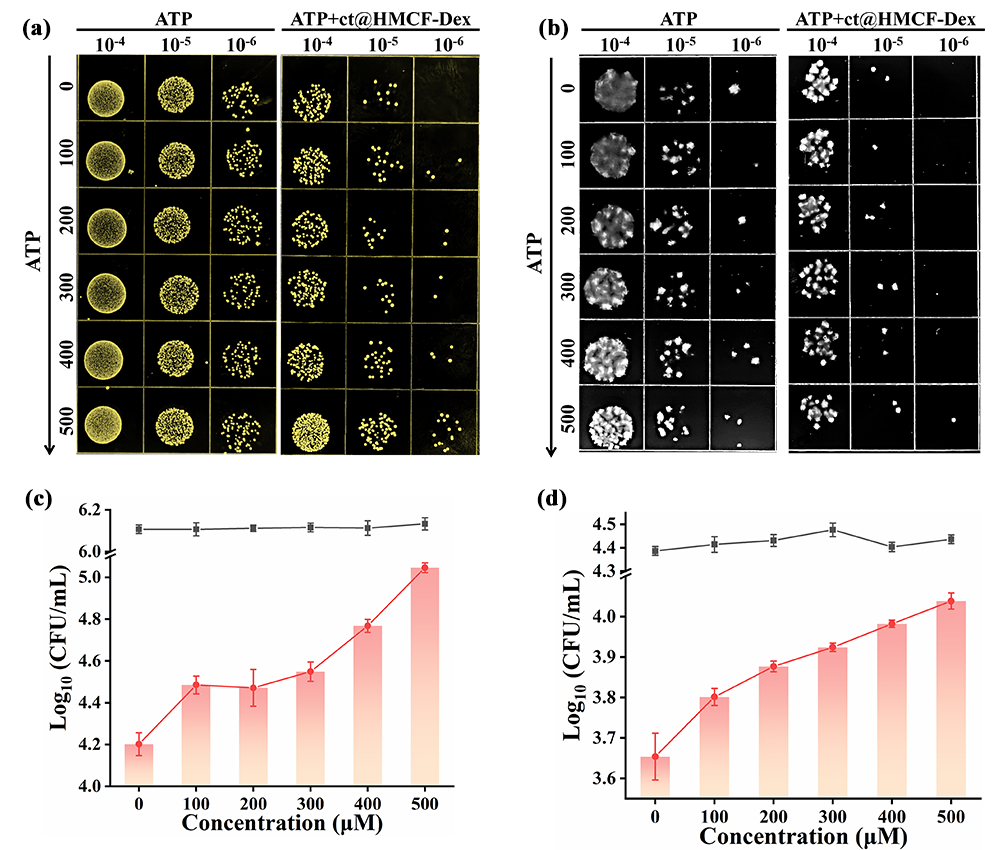


**Figure S22.** ATP inhibition of ct@HMCF-Dex-induced MRSA (a) and *P. aeruginosa* (b) death. Quantitative analysis of MRSA (c) and *P. aeruginosa* (d) after different treatments (*n*=3). Error bars represent means ± SD.


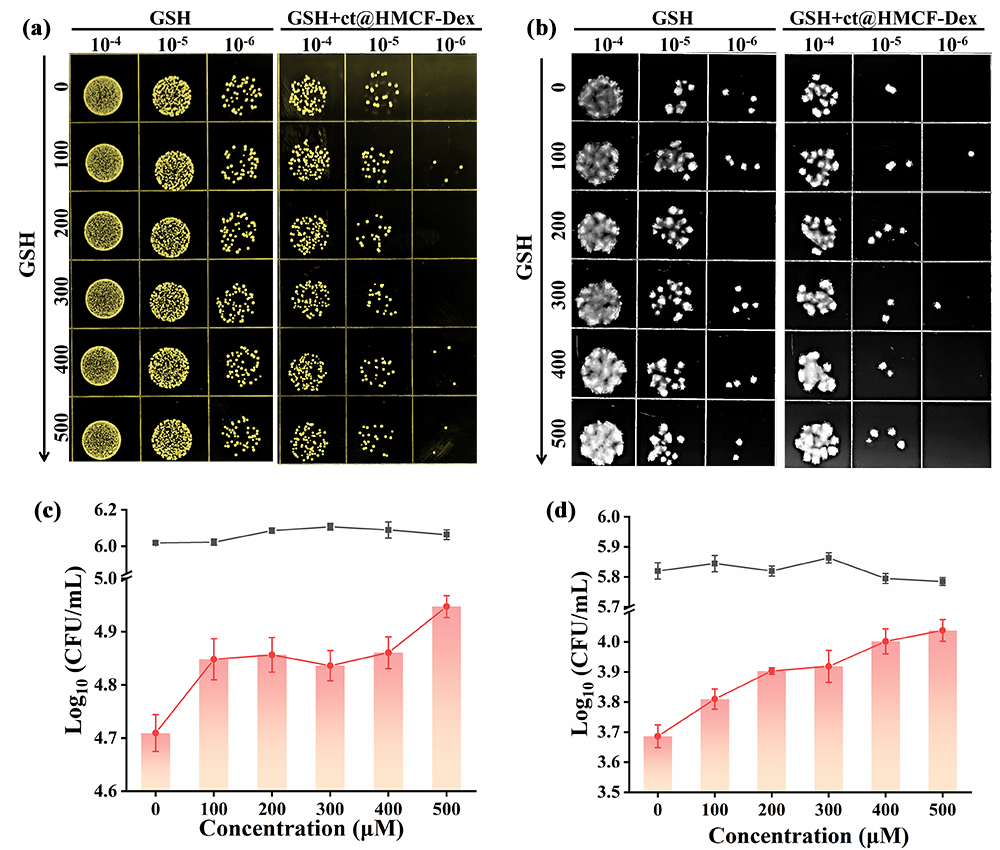


**Figure S23.** GSH inhibition of ct@HMCF-Dex-induced MRSA (a) and *P. aeruginosa* (b) death. Quantitative analysis of MRSA (c) and *P. aeruginosa* (d) after different treatments (*n*=3). Error bars represent means ± SD.


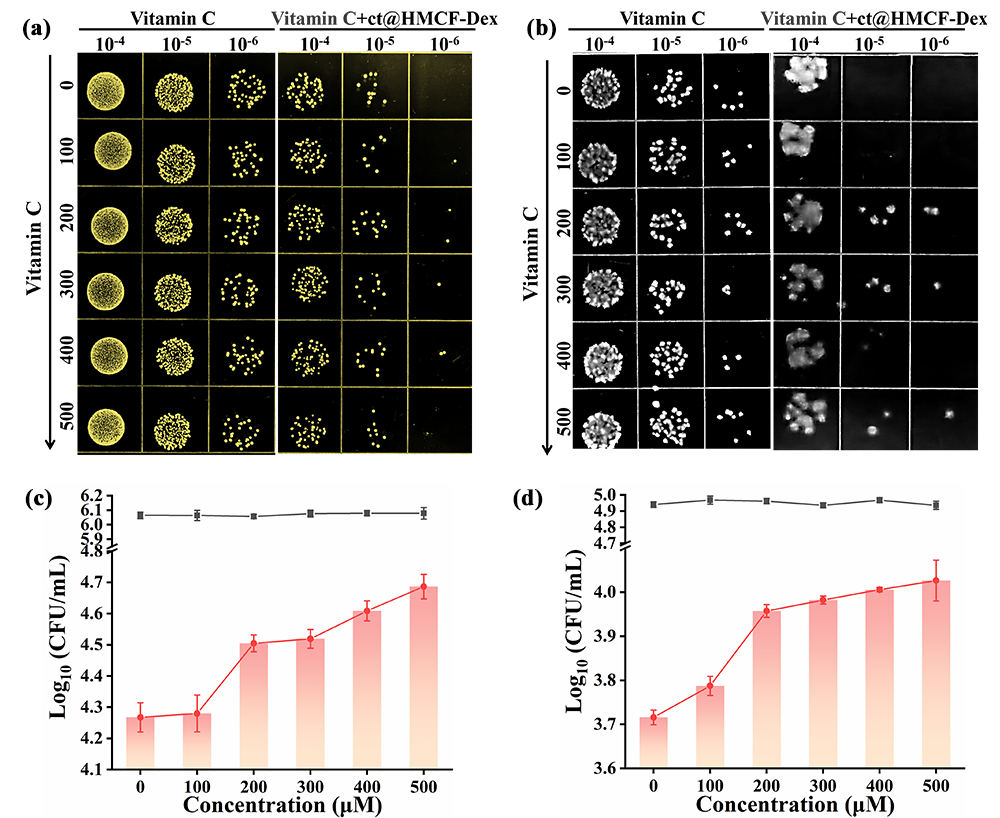


**Figure S24.** Vitamin C inhibition of ct@HMCF-Dex-induced MRSA (a) and *P. aeruginosa* (b) death. Quantitative analysis of MRSA (c) and *P. aeruginosa* (d) after different treatments (*n*=3). Error bars represent means ± SD.


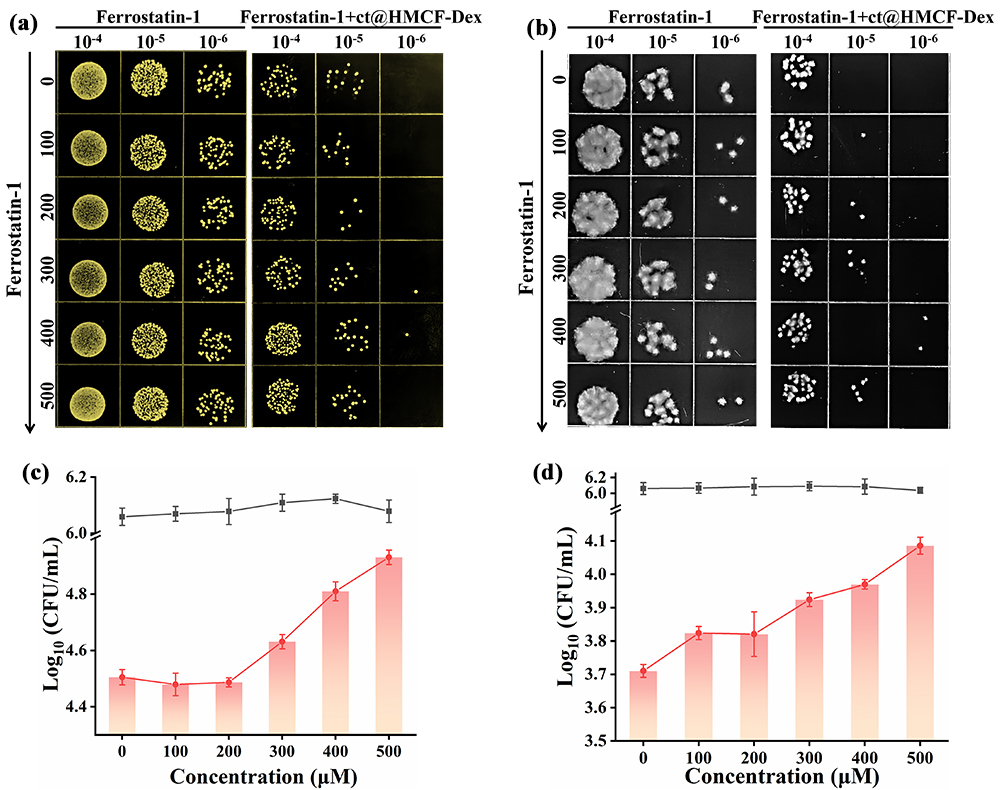


**Figure S25.** Ferrostatin-1 inhibition of ct@HMCF-Dex induced MRSA (a) and *P. aeruginosa* (b) death. Quantitative analysis of MRSA (c) and *P. aeruginosa* (d) after different treatments (*n*=3). Error bars represent means ± SD.

**
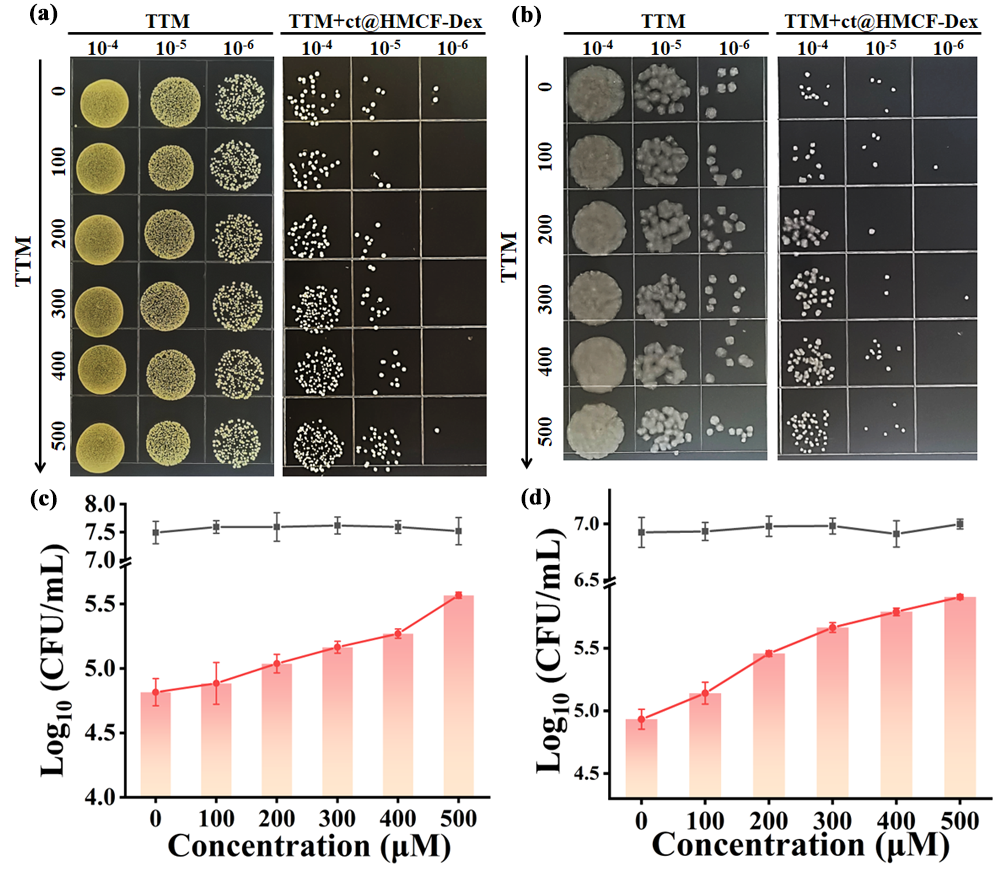
**

**Figure S26.** Tetrathiomolybdate (TTM) inhibition of ct@HMCF-Dex induced MRSA (a) and *P. aeruginosa* (b) death. Quantitative analysis of MRSA (c) and *P. aeruginosa* (d) after different treatments (*n*=3). Error bars represent means ± SD.


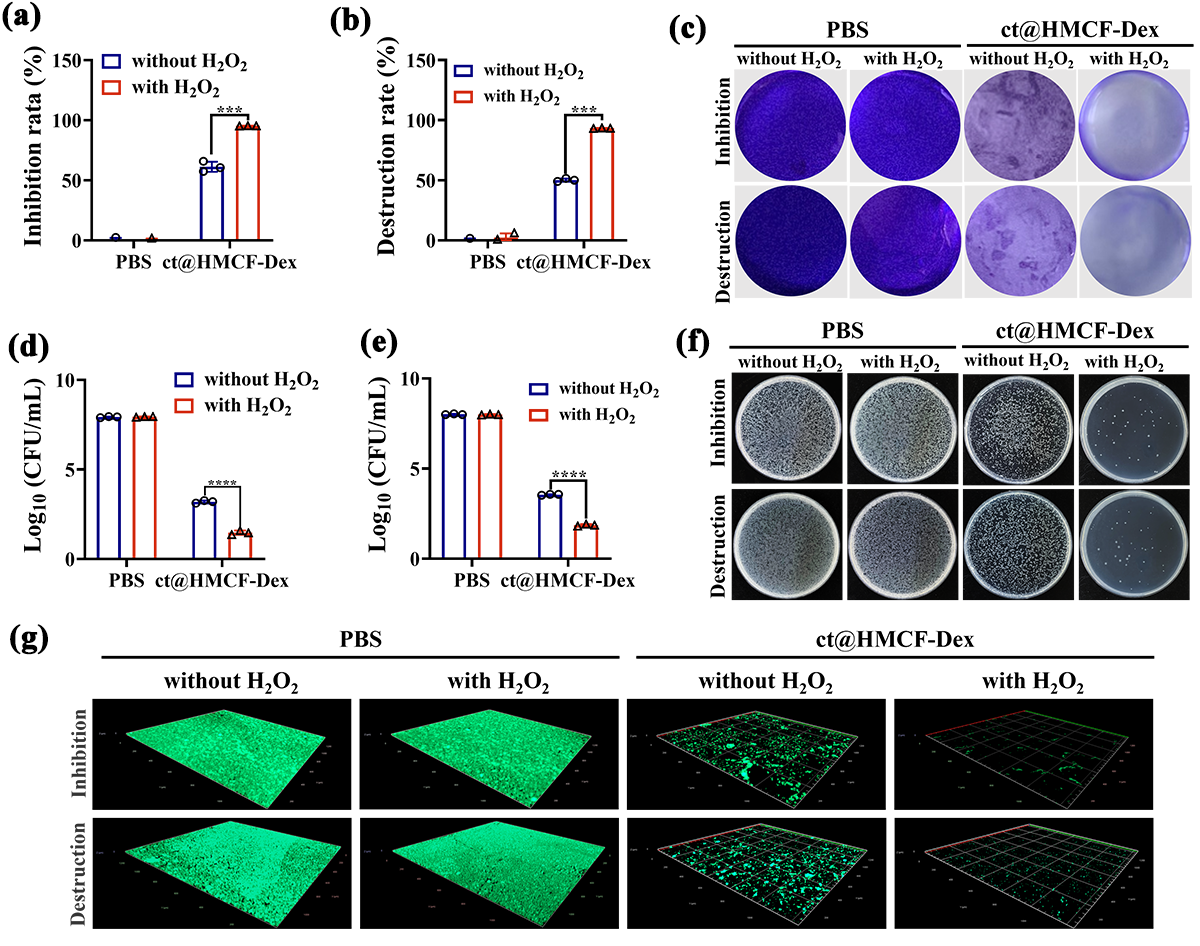


**Figure S27.** Inhibition rate (a) and destruction rate (b) of ct@HMCF-Dex against *P. aeruginosa* biofilms and corresponding crystal violet stained photographs (c) (*n*=3). CFU counts of *P. aeruginosa* biofilm inhibition (d) and disruption (e) after different treatments (*n*=3). Bacterial colonies (f) and fluorescence images (g) of *P. aeruginosa* biofilm inhibition and disruption after different treatments. Error bars represent means ± SD. Differences between groups were tested using one-way ANOVA followed by Tukey's multiple comparisons test. ^***^*p* < 0.001, ^****^*p* < 0.0001.


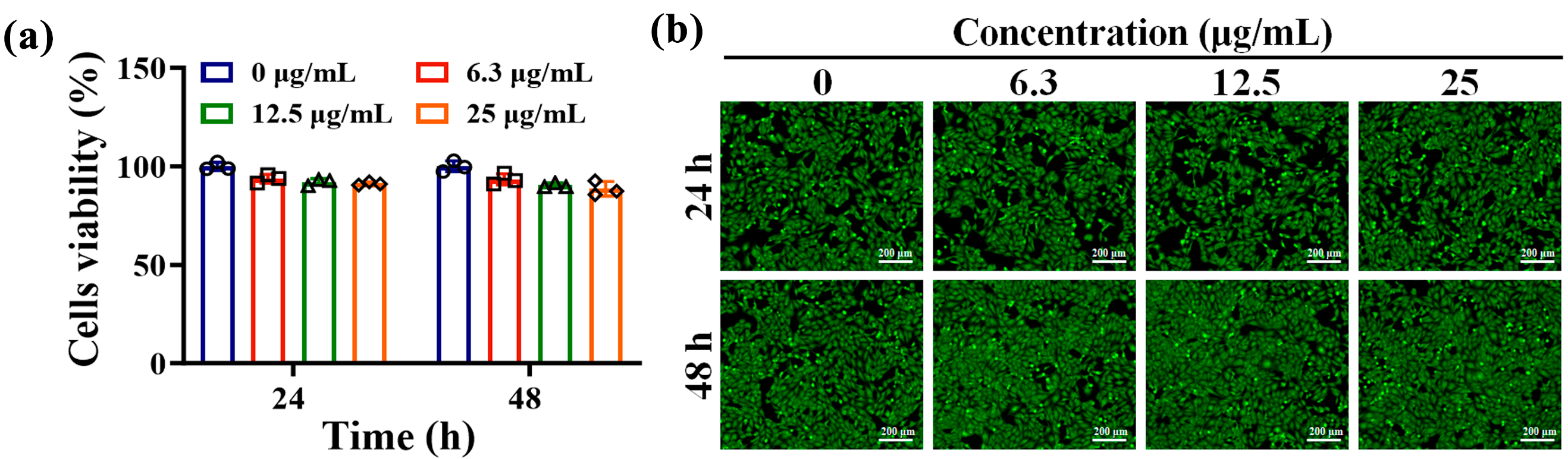


**Figure S28.** Cytotoxicity (a) of different concentrations of ct@HMCF-Dex on BEAS-2B cells and corresponding images of cell viability staining (b). (*n*=3). Error bars represent means ± SD.


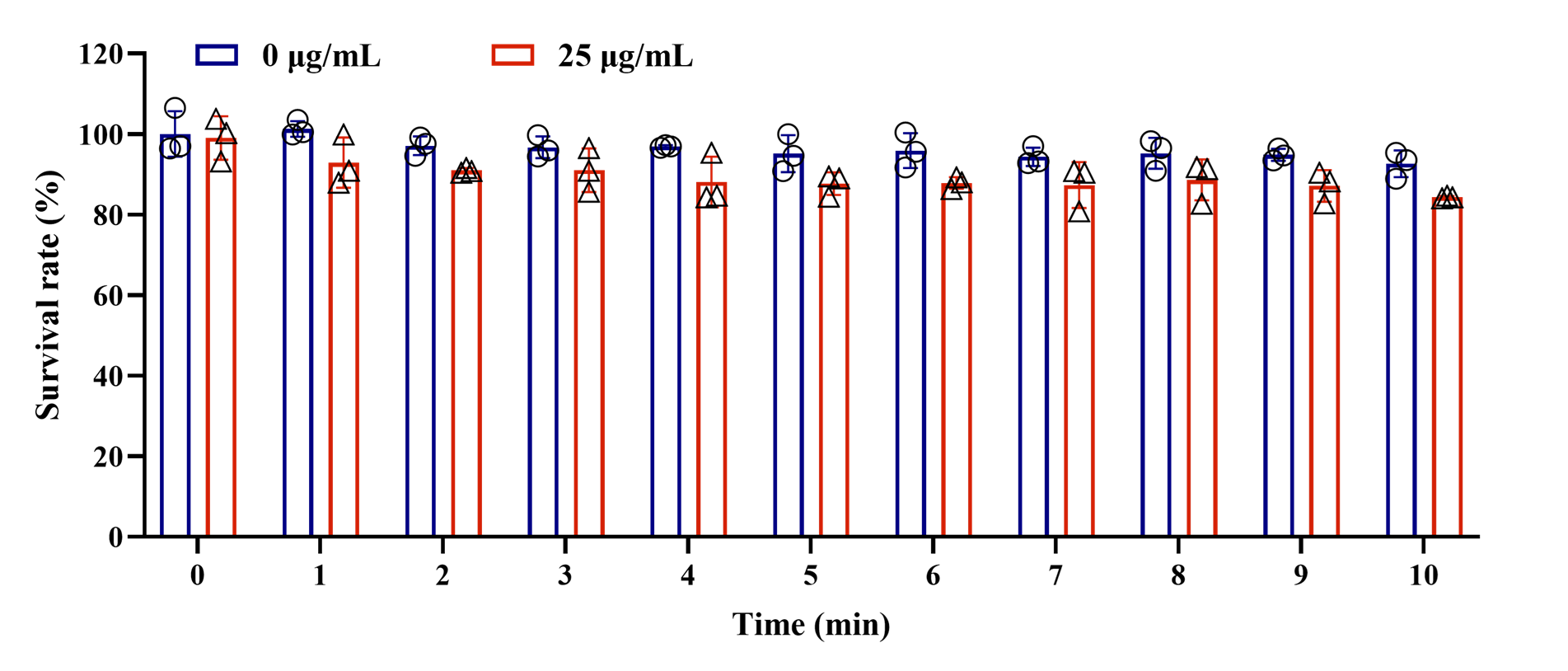


**Figure S2****9.** Toxic effects of ct@HMCF-Dex on NIH/3T3 at different exposure times (*n*=3). Error bars represent means ± SD.


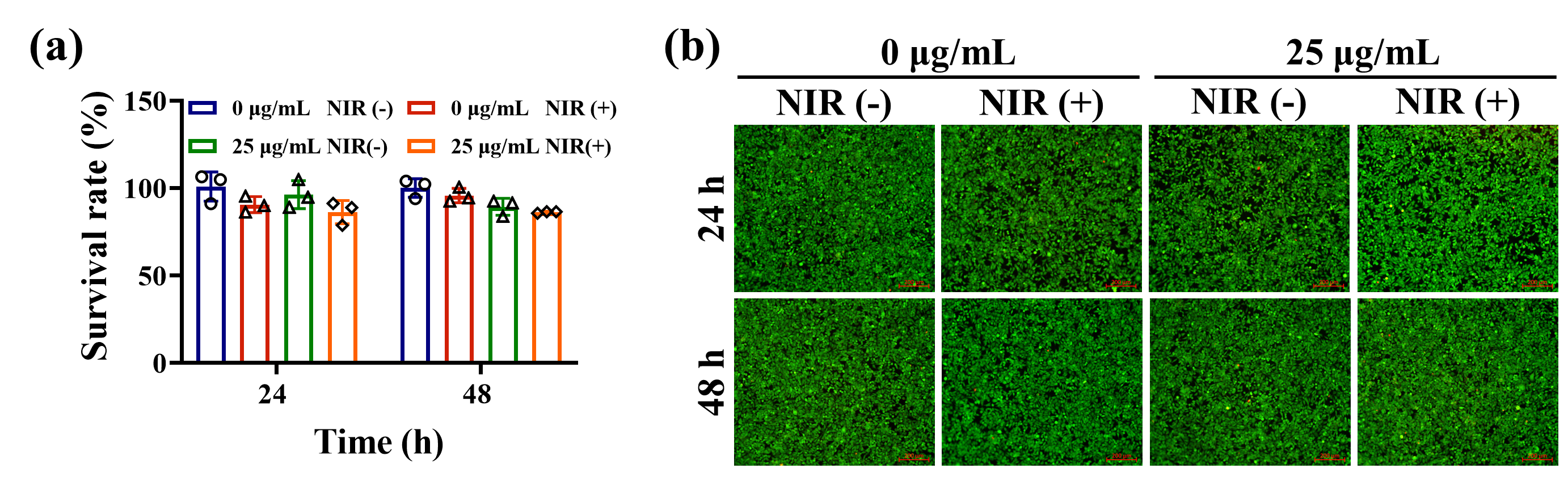


**Figure S30.** (a) The cytotoxicity effect of ct@HMCF-Dex (25 μg/mL) on NIH/3T3 cells under NIR light and no-light conditions and (b) corresponding images of bacterial live-dead staining (*n*=3). Error bars represent means ± SD.


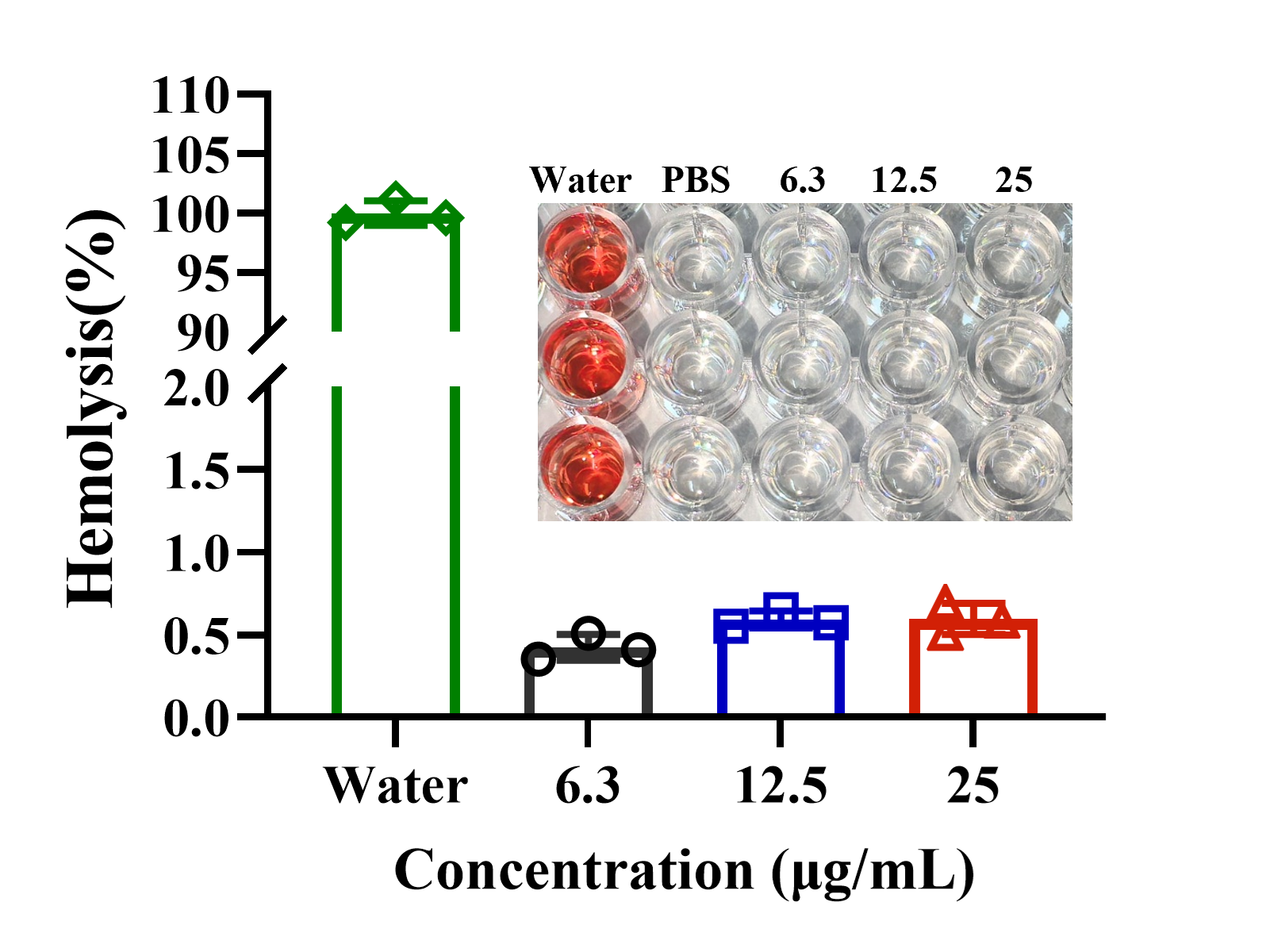


**Figure S31.** Hemolytic activity assay of ct@HMCF-Dex, inset is the corresponding photograph (*n*=3). Error bars represent means ± SD.


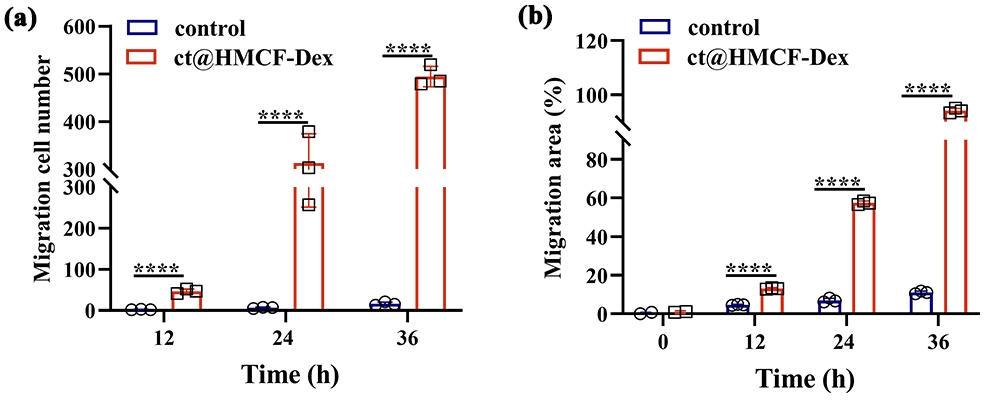


**Figure S32.** Quantitative calculation of the number of cells migrating in Transwell assay (a) and cell scratch assay (b) (*n*=3). Error bars represent means ± SD. Differences between groups were tested using one-way ANOVA followed by Tukey's multiple comparisons test. ^****^*p* < 0.0001.


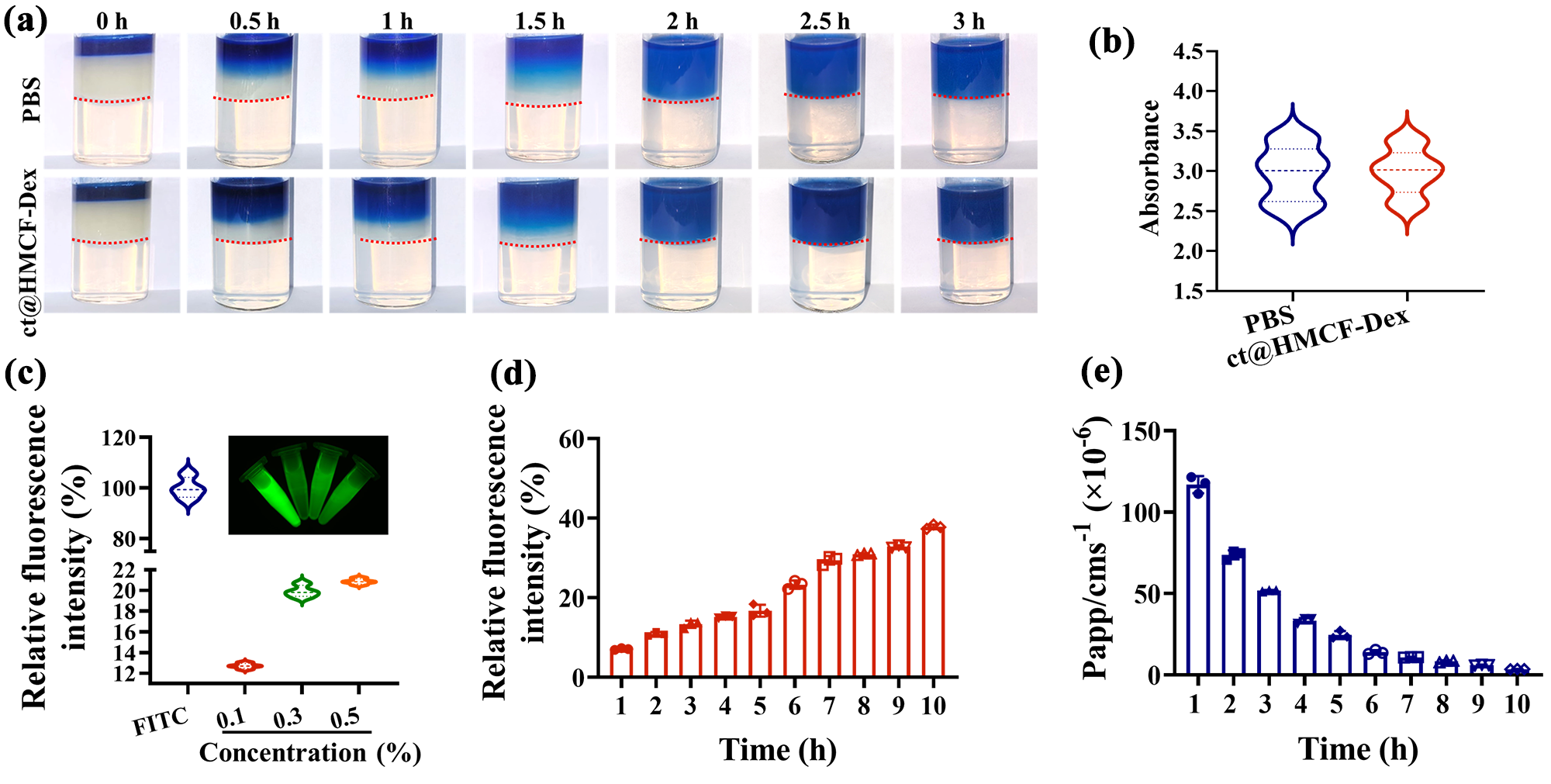


**Figure S33.** (a) Photographs of PBS and ct@HMCF-Dex penetrating the artificial mucus at different time points. (b) The absorbance of the 595 nm gelatin layer after 3 h of incubation (*n*=3). (c) Percentage of ct@HMCF-Dex-FITC forming aggregates with different concentrations of mucin and total fluorescence intensity of ct@HMCF-Dex-FITC as 100%, insets are corresponding fluorescence images (*n*=3). (d) Relative fluorescence intensity of FITC in Transwell receiver plate solution after incubating ct@HMCF-Dex-FITC for different times (*n*=3). (e) Osmotic index of ct@HMCF-Dex-FITC after incubation with ct@HMCF-Dex-FITC for different times (*n*=3). Error bars represent means ± SD.


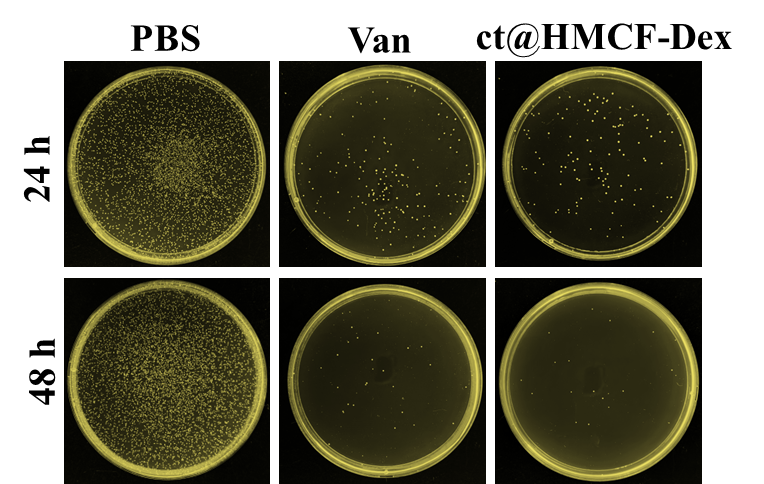


**Figure S34.** Bacterial colonization in lung tissue of mice after different treatments.


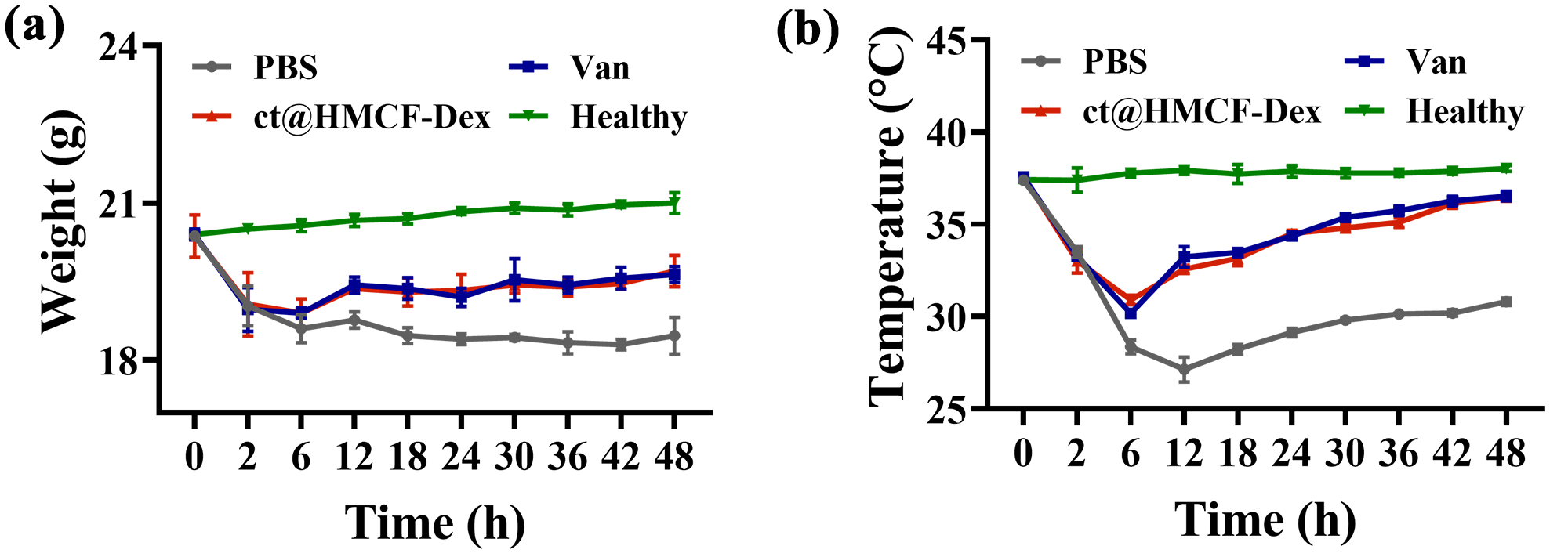


**Figure S35.**Changes in body weight (a) and body temperature (b) of mice after different treatments (*n*=5). Error bars represent means ± SD.


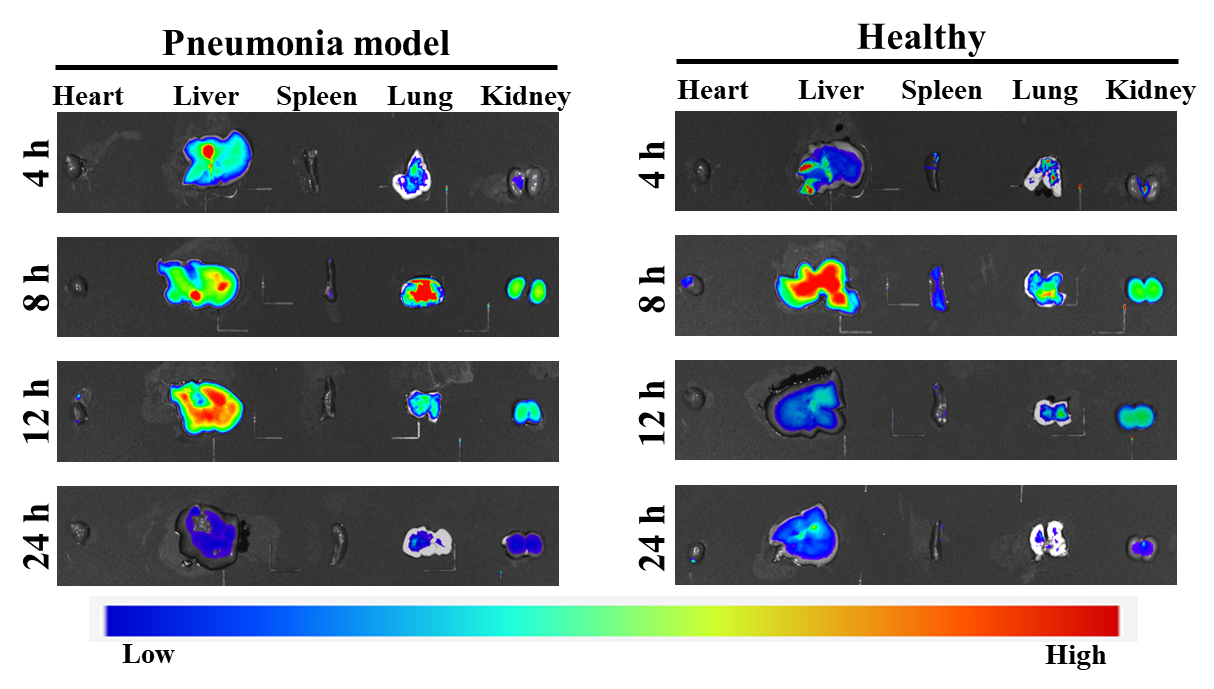


**Figure S36.** Fluorescence images of major organs at 4 h, 8 h, 12 h and 24 h post-injection.


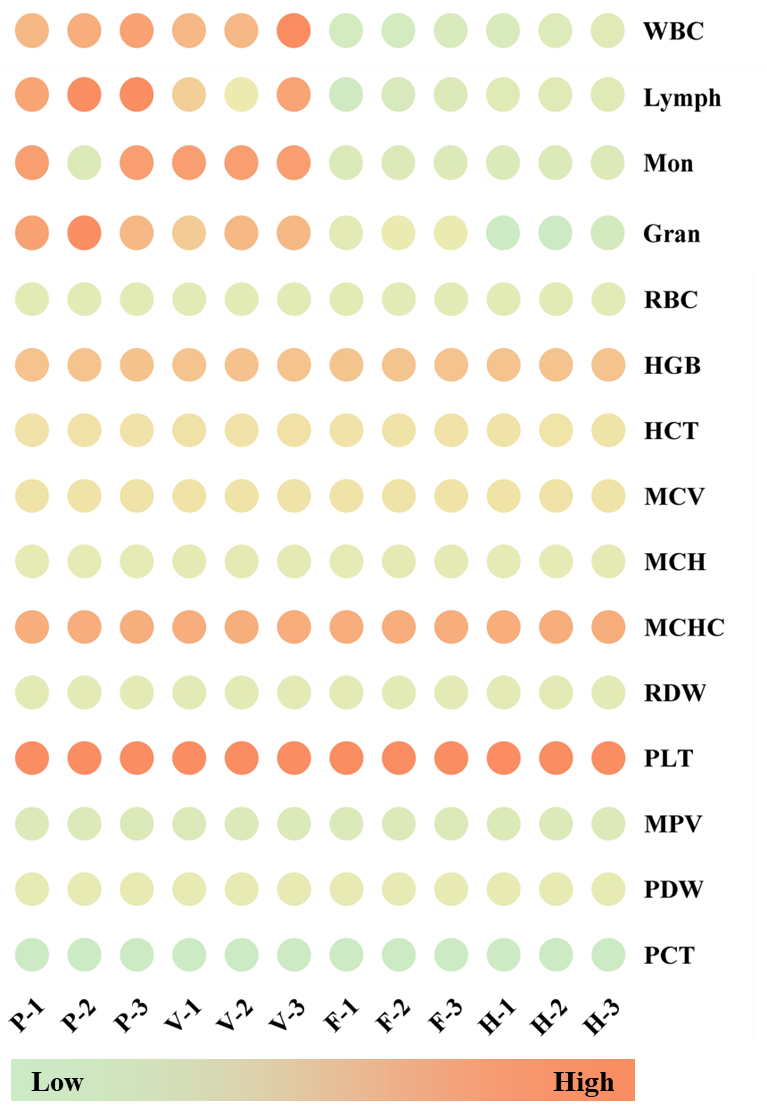


**Figure S37.** Blood routine at the 48th hour of mice with various treatment groups of pneumonia models (*n*=3). P-1-P-3: treatment with PBS; V-1-V-3: treatment with 0.9% normal saline; F-1-F-3: treatment with ct@HMCF-Dex; H-1-H-3: Healthy.


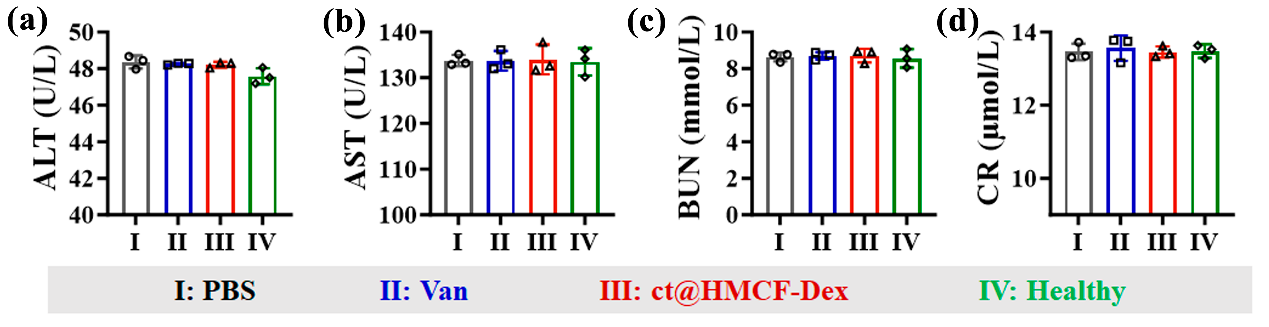


**Figure S38.** Changes in serum levels of alanine aminotransferase (ALT), aspartate aminotransferase (AST), blood urea nitrogen (BUN), and creatinine (CR) in mice of pneumonia model after different treatments (*n*=3). Error bars represent means ± SD.


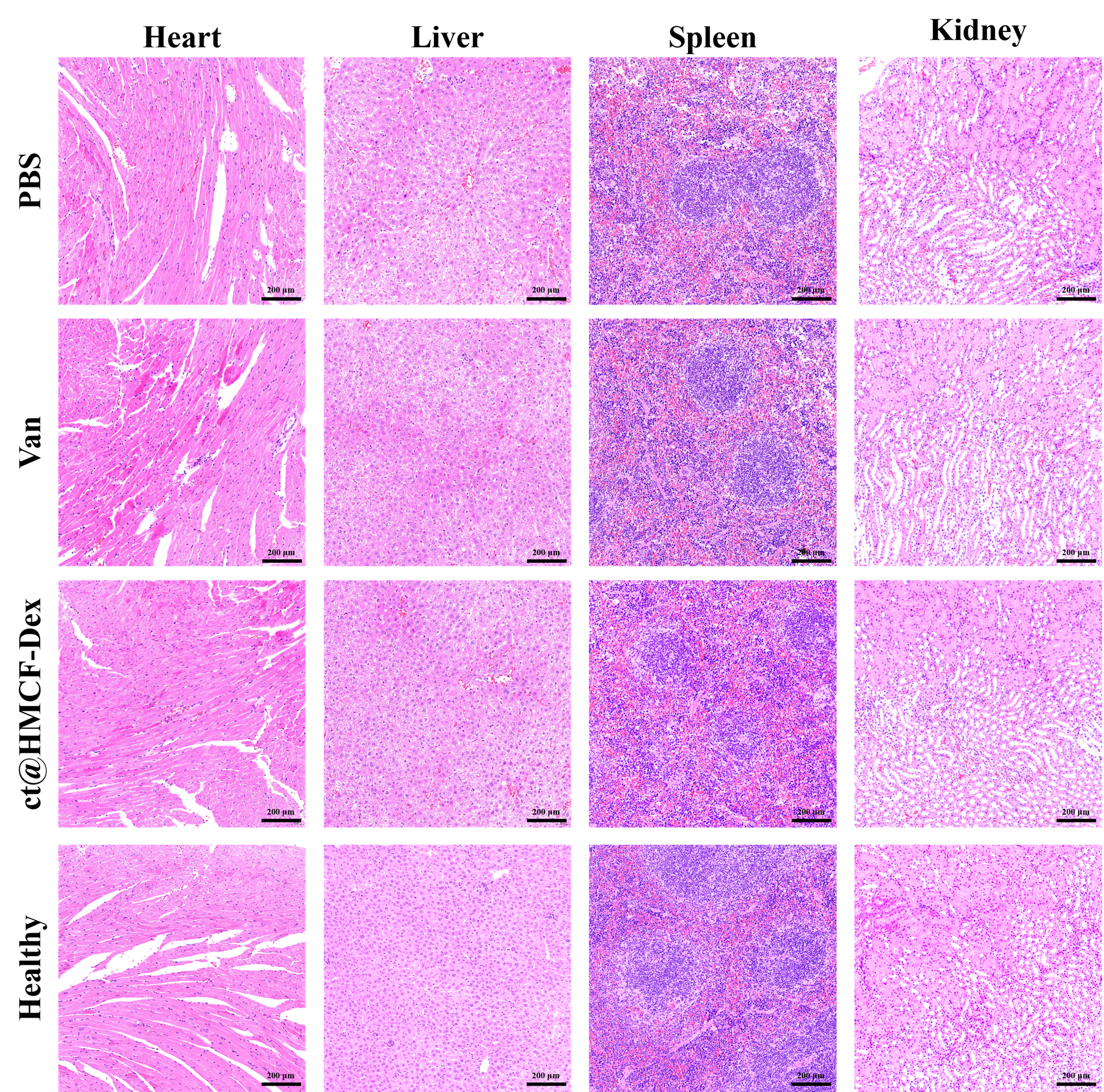


**Figure S39.** H&E staining photographs of tissue sections from major internal organs (heart, liver, spleen, kidney) of different treatment after 48 h.


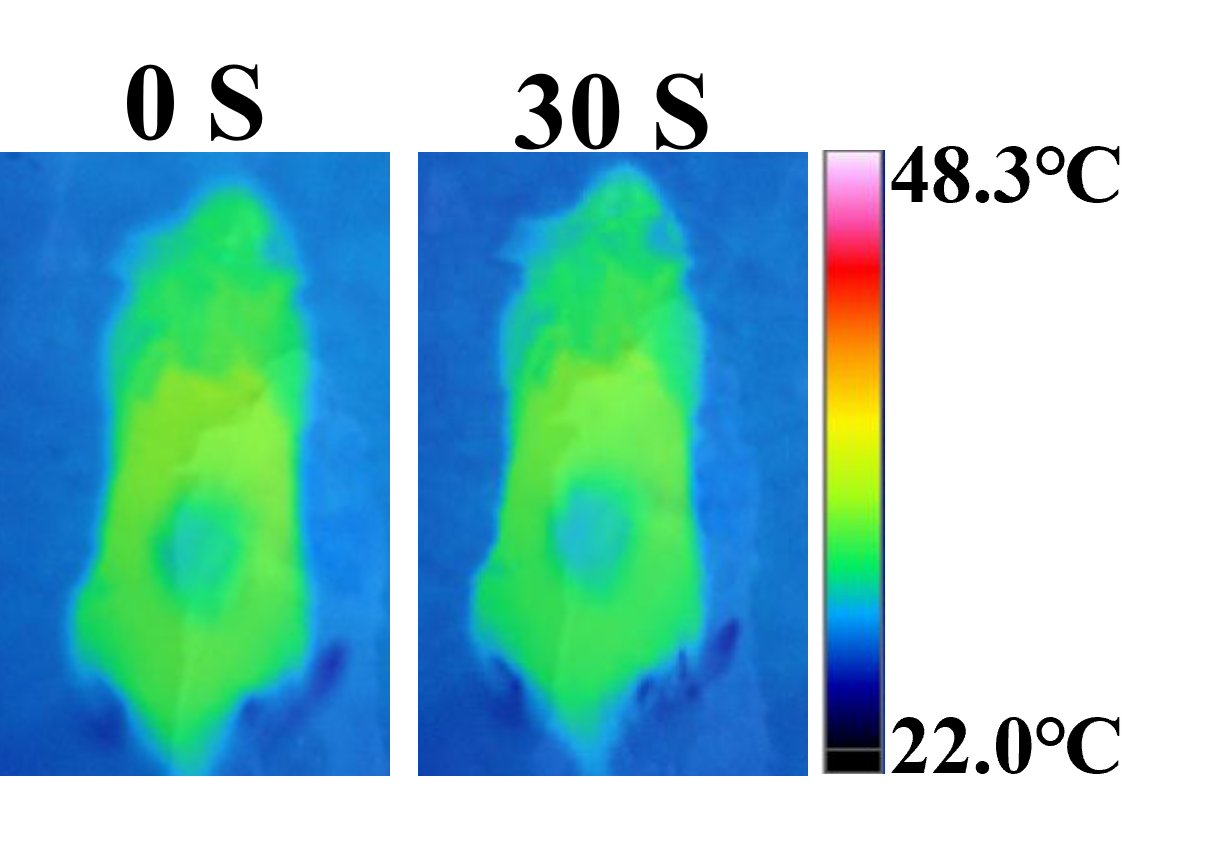


**Figure S40.** *In vivo* thermal infrared image of mice in the PBS group.


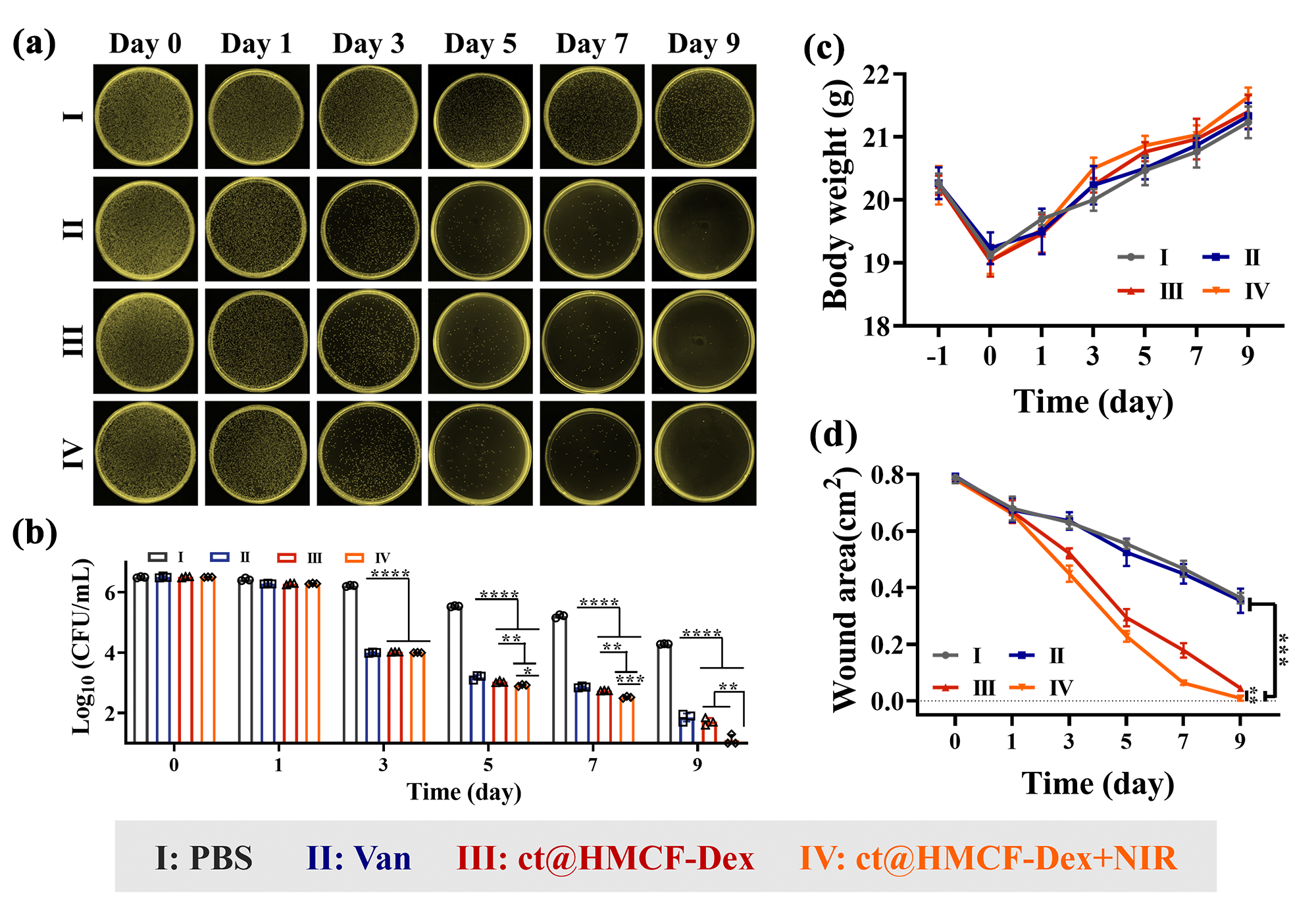


**Figure S41.** (a) Plates of wound colonies in mice after different days of treatment. (b) The number of bacteria surviving in the skin tissue was counted after treatment (*n*=3). Changes in body weight (c) and wound area (d) of mice after different treatments (*n*=3). Error bars represent means ± SD. Differences between groups were tested using one-way ANOVA followed by Tukey's multiple comparisons test. ^*^*p* < 0.05, ^**^*p* < 0.01, ^***^*p* < 0.001, ^****^*p* < 0.0001.


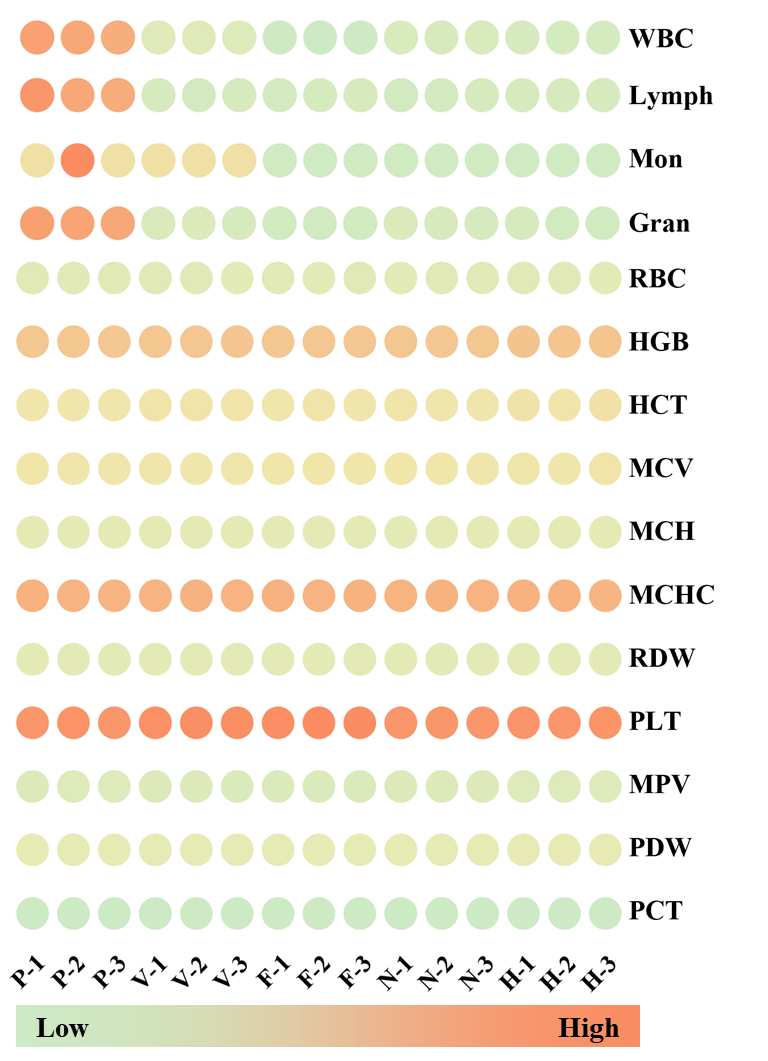


**Figure S42.** Blood routine of the 9th day of mice with various treatment groups of MRSA-infected wound models (*n*=3). P-1-P-3: treatment with PBS; V-1-V-3: treatment with 0.9% normal saline; F-1-F-3: treatment with ct@HMCF-Dex; N-1-N-3: treatment with ct@HMCF-Dex+NIR, H-1-H-3: Healthy.


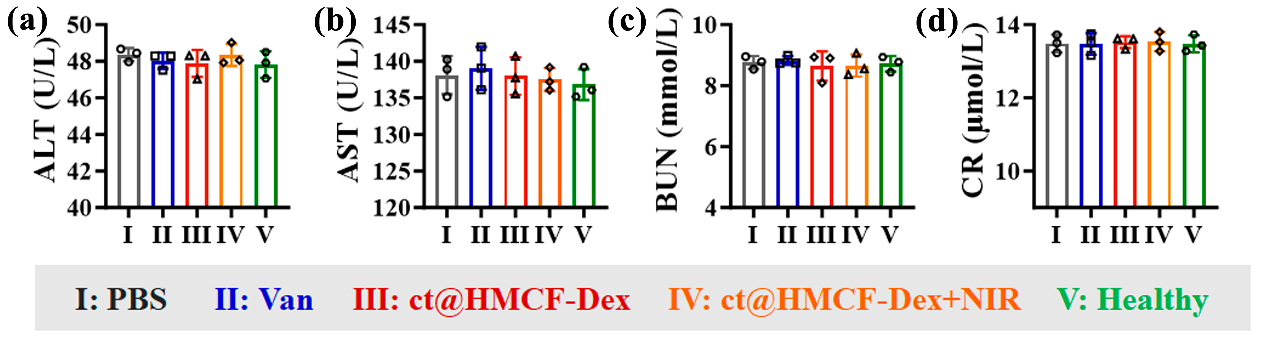


**Figure S43.** Changes in serum levels of alanine aminotransferase (ALT), aspartate aminotransferase (AST), blood urea nitrogen (BUN), and creatinine (CR) in mice of wound model after different treatments (*n*=3). Error bars represent means ± SD.


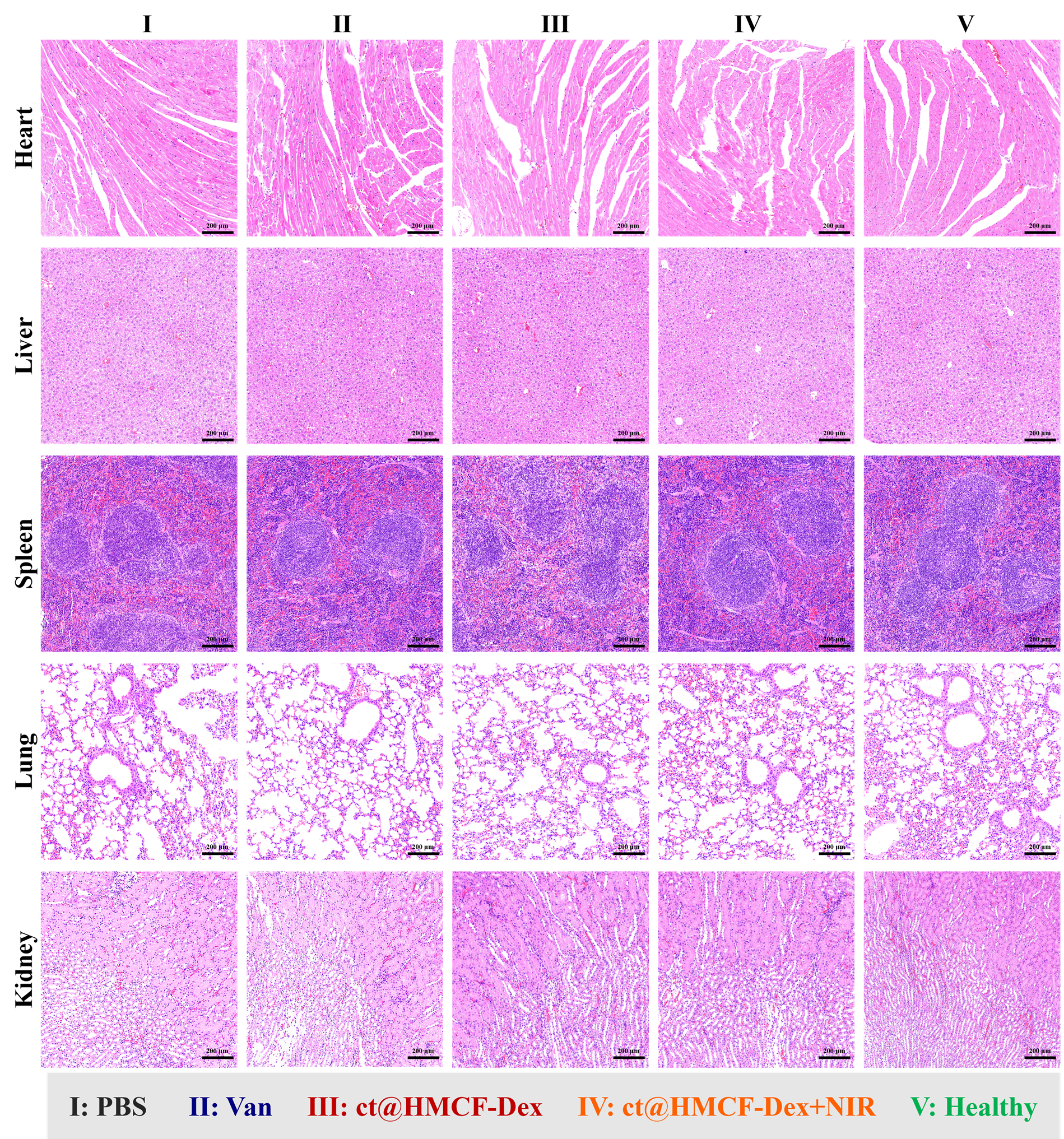


**Figure S44.** H&E staining photographs of tissue sections from major internal organs (heart, liver, spleen, lung, kidney) on day 9 of treatment. I: treatmet with 0.9% normal saline; II: treatment with Van; Ⅲ: treatment with ct@HMCF-Dex; Ⅳ: treatment with ct@HMCF-Dex+NIR, V: healthy.

1. Z. Xiao , J. Cao, J. Liu, T. Du, X. Du

   State Key Laboratory of Food Nutrition and Safety, College of Food Science and Engineering, Tianjin University of Science and Technology, Tianjin 300457, PR China

   E-mail: [tingdu@tust.edu.cn](mailto:tingdu@tust.edu.cn), xjdu@tust.edu.cn

   Z. Song

   College of Chemistry, Huazhong Agricultural University, Wuhan 430070, PR China [↑](#footnote-ref-1)
